# DNA damage response in breast cancer and its significant role in guiding novel precise therapies

**DOI:** 10.1186/s40364-024-00653-2

**Published:** 2024-09-27

**Authors:** Jiayi Li, Ziqi Jia, Lin Dong, Heng Cao, Yansong Huang, Hengyi Xu, Zhixuan Xie, Yiwen Jiang, Xiang Wang, Jiaqi Liu

**Affiliations:** 1 Department of Breast Surgery, National Cancer Center/National Clinical Research Center for Cancer/Cancer Hospital, Chinese Academy of Medical Sciences and Peking Union Medical College, Beijing, 100021 China; 2 School of Clinical Medicine, Chinese Academy of Medical Sciences and Peking Union Medical College, Beijing, 100005 China; 3 Department of Pathology, National Cancer Center/National Clinical Research Center for Cancer/Cancer Hospital, Chinese Academy of Medical Sciences and Peking Union Medical College, Beijing, 100021 China; 4 State Key Laboratory of Molecular Oncology, National Cancer Center/National Clinical Research Center for Cancer/Cancer Hospital, Chinese Academy of Medical Sciences and Peking Union Medical College, Beijing, 100021 China

**Keywords:** DNA damage response, Breast cancer, Homogenous recombination deficiency, Poly (ADP-ribose) polymerase (PARP) inhibitor

## Abstract

DNA damage response (DDR) deficiency has been one of the emerging targets in treating breast cancer in recent years. On the one hand, DDR coordinates cell cycle and signal transduction, whose dysfunction may lead to cell apoptosis, genomic instability, and tumor development. Conversely, DDR deficiency is an intrinsic feature of tumors that underlies their response to treatments that inflict DNA damage. In this review, we systematically explore various mechanisms of DDR, the rationale and research advances in DDR-targeted drugs in breast cancer, and discuss the challenges in its clinical applications. Notably, poly (ADP-ribose) polymerase (PARP) inhibitors have demonstrated favorable efficacy and safety in breast cancer with high homogenous recombination deficiency (HRD) status in a series of clinical trials. Moreover, several studies on novel DDR-related molecules are actively exploring to target tumors that become resistant to PARP inhibition. Before further clinical application of new regimens or drugs, novel and standardized biomarkers are needed to develop for accurately characterizing the benefit population and predicting efficacy. Despite the promising efficacy of DDR-related treatments, challenges of off-target toxicity and drug resistance need to be addressed. Strategies to overcome drug resistance await further exploration on DDR mechanisms, and combined targeted drugs or immunotherapy will hopefully provide more precise or combined strategies and expand potential responsive populations.

**Supplementary Information:**

The online version contains supplementary material available at 10.1186/s40364-024-00653-2.

## Introduction

Breast cancer is the second most common malignant tumors and is the 4th leading cause of cancer-related deaths [1]. Systemic treatment is crucial for improving the prognosis of both early-stage and metastatic breast cancer [2]. DNA damage response (DDR) pathways are important factors in the pathogenesis of breast cancer [3], with about 10% of breast cancers being caused by pathogenic germline mutations in homogenous recombination (HR)-related genes such as *BRCA1/2 * [4–6]. Breast cancer phenotypes also exist in Lynch syndrome (LS) related to microsatellite instability (MSI) and Li-Fraumeni syndrome caused by *TP53* mutations [3]. Moreover, drugs targeting the DDR pathway and related therapies (such as radiotherapy) have been emerging in recent years [7]. DNA repair defects, subsequent increased replication stress, and the arrest of the cell cycle facilitate cancer cells more susceptible to DDR inhibition than normal cells [8]. Due to endogenous or exogenous pressures cell DNA suffers from during replication, targeting DDR can induce DNA damage, resulting in cell cycle arrest and tumor cell death [9]. It is worth our while to thoroughly summarize and analyze the current research progress in order to find suitable populations and optimal combination strategies for each therapy.

In parallel with the advances in introducing DDR as a potential therapeutic target, a range of inhibitors targeting DDR components have emerged. More importantly, cancers with a high rate of cell division, such as breast cancer, are more responsive to the DDR treatments [10]. Among them, poly-ADP-ribose polymerase (PARP) inhibitors are the most commonly used and related to the HR DNA repair mechanism [11]. PARP inhibitors have first obtained their indications in ovarian cancer and *BRCA1/2*-mutated breast cancer [12, 13]. However, due to the low incidence of *BRCA1/2* mutations in breast cancer patients, relying solely on genetic mutations to identify responsive populations results in a limited potential population [14]. To enable more patients with refractory tumors to benefit from DDR-related therapies, a deeper and broader understanding of the biological functions of DNA repair and related biomarkers for breast cancer stratification is needed.

**Fig. 1:**
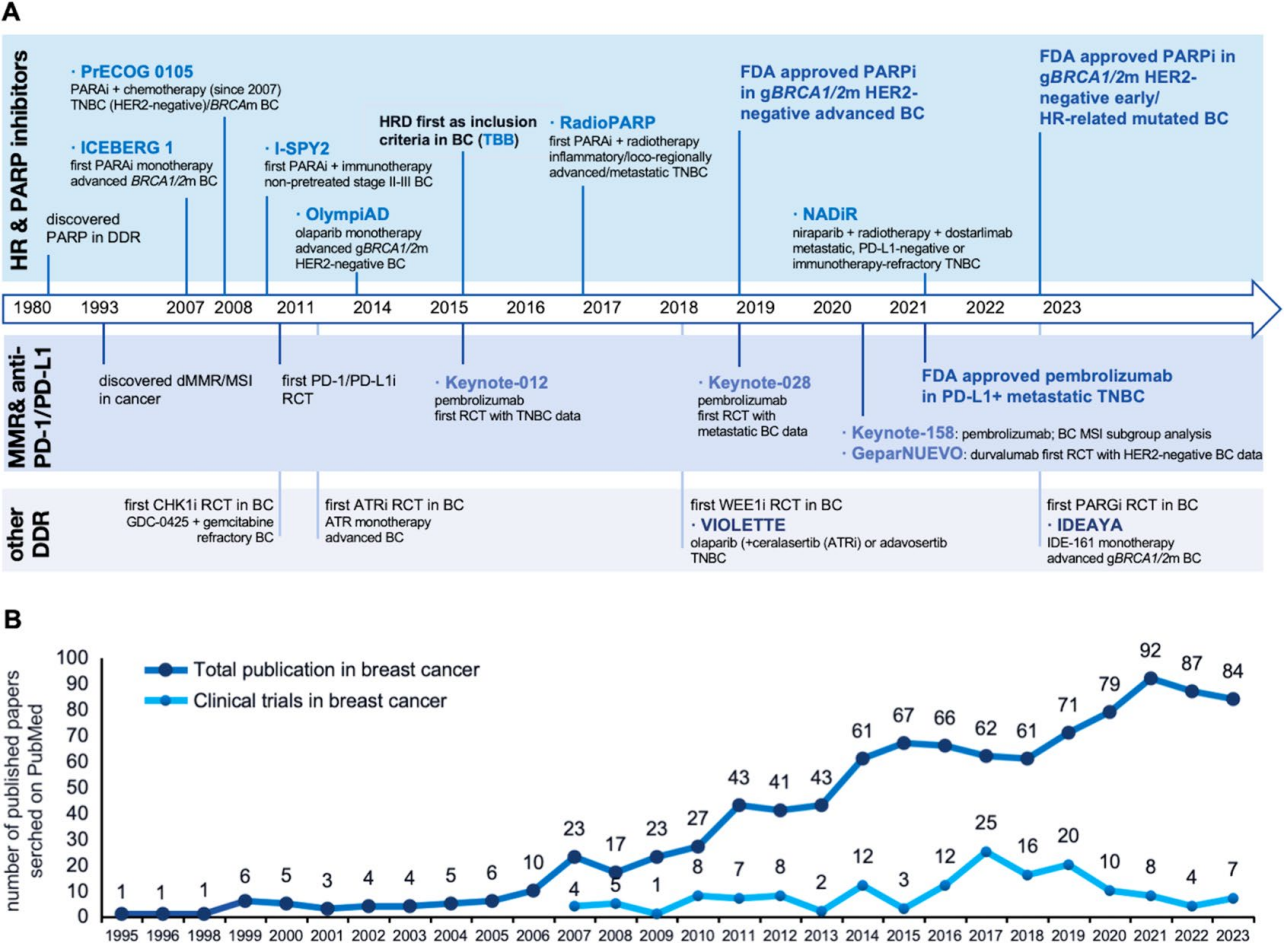
Summary of (**A**) history of studying DNA damage response (HR, MMR, and other DDR mechanisms), targeted therapies, and their clinical trials in breast cancer (**B**) total publication papers of DDR and DDR-related clinical trials in in breast cancer. **A** DDR in breast cancer has drawn much attention in targeted treatment since 1980 when PARP has been discovered in SSB repairing. The timeline in the upper part has summarized the history of DDR discovery, important mechanisms HR and MMR in breast cancer, representative clinical trials, and the evolution of indications of DDR-related therapies. **B** The line chart in the bottom has demonstrated publications and clinical trials on DDR in breast cancer since 1980. In 2007, olaparib was the first DDR-related drug under clinical trials, thus the total published papers increased. Clinical trials have increased since breast cancer had the first indications of PARP inhibitors approved by FDA in 2017. DDR, DNA damage response; SSB, DNA single-strand break; HRD, homogenous recombination deficiency; MMR, mismatch repair; MSI-H, high microsatellite instability; dMMR, deficient mismatch repair; PD-1, programmed cell death-1 protein; PD-L1, programmed cell death-1 ligand protein; RCT, randomized clinical trial; BC, breast cancer; TNBC, triple-negative breast cancer; MSI, microsatellite instability; CRC, colorectal cancer; PARP, poly-ADP-ribose polymerase; CHEK1, checkpoint kinase 1; ATR, ataxia telangiectasia and Rad3-related; PARG, poly (ADP-ribose) glycohydrolase

In this review, we first focus on various DNA repair pathways and discuss the underlying mechanisms for the application of DDR inhibitors. With the past preclinical evidence and ongoing clinical trials in breast cancer (history of studying DDR in breast cancer (Fig. 1), we have summarized the DDR inhibitor practice and strategies for drug resistance, with a special concentration on the combined therapies with chemotherapy, radiotherapy, immunotherapies, and other DDR inhibitors. This summarization helps to target specific DDR-related pathways with tailored therapies and precise predictive biomarkers, further developing potential comprehensive strategies for treating refractory breast malignancy.

### Diverse mechanisms of DNA damage repair

**Fig. 2:**
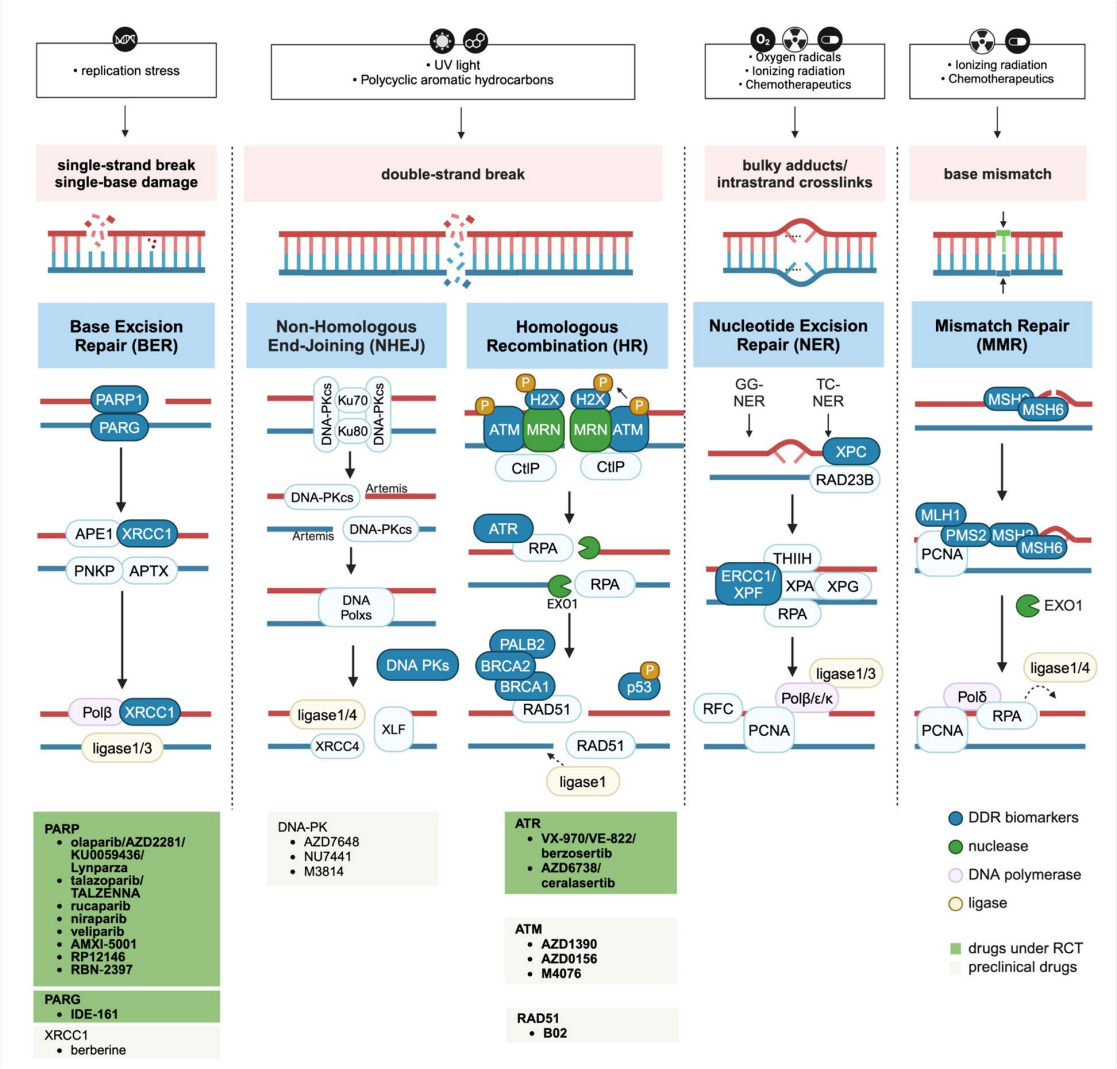
Summary of DNA damage response mechanisms and targeted therapies in breast cancer. BER is the primary pathway to repair SBSs. PARP binds to damaged SBSs to recruit DNA repair effectors. First, aberrant bases are identified and cleaved by DNA glycosylation enzymes. Cleavage of damaged N-glycosidic bond of the base results in an AP site creation and AP spots can be recognized by AP endonucleases. APE cleaves to the AP site to generate 3′-OH and 5′-dR termini. Intrinsic dRP lyase activity of DNA Pol β cleaves dRP residue to produce 5′-phosphates. DSBs are caused by UV light and aromatic chemicals and repaired by NHEJ or HR. In NHEJ, 53BP1 and RIF1 localize to DSBs, resulting in BRCA1 recruitment inhibition, blocking DNA excision, and promoting NHEJ repair pathway. The essential components of NHEJ are heterodimers consisting of Ku70/Ku80 and catalytic subunits of DNA-PKcs, identifying DSB and activating downstream signaling factors, including XRCC4, XLF, and DNA ligase IV. HR is initiated by MRN binds to DSB terminal, and MRN recruits and activates ATM and CHEK2 and phosphorylates downstream substrates. CtIP binds to BRCA1 and nucleases EXO1, further interacts with MRN and excises DSB ends together. Excised DNA ends are coated with hyperphosphorylated single-stranded RPA to form a nucleoprotein filament. γH2AX is activated and phosphorylated by apical kinases (ATM and ATR). Diffusion of γH2AX along chromosomes recruits and accumulates additional DDR proteins, including 53BP1 and BRCA1. Under PALB2 localization, BRCA2 binds to BRCA1, promotes recombinant enzyme RAD51 loading on ssDNA, and finally RAD51 prevents secondary structure formation and shields DNA ends from degradation. NER removes bulky adducts and crosslinks lesions, especially those induced by UV light. NER employs TC-NER and GG-NER, both utilizing XPC and RAD23 to excise the damage. DNA-binding proteins XPA and RPA enhance translocation and damage-verification activity of TFIIH and stabilize and orient endonucleases XPF/ERCC1 and XPG. Replication factors involved include PCNA and RFC, DNA polymerases δ, ε, and κ, and ligases I and III. MMR corrects base match errors. First, MutS (MSH2/MSH6) determines base pair error. MutS/MutL complex binds to MutH (MLH1/PMS2). MutH is then activated to cleave the unmethylated strands. Exonuclease removes the nascent strand from the cut point to error base-pair portion. Other DDR pathways including DNA damage tolerance, interstrand crosslink repair and direct damage reversal are not demonstrated. Among proteins involved in DDR, PARP, PARG, and XRCC1 in BER, DNA-PK in NHEJ, and ATR, ATM, and RAD51 in HR can be targeted in DDR-related therapies. Drugs under RCT and in preclinical stage are summarized below (searching results on https://clinicaltrials.gov by 31st August 2024). BER, base excision repair; SSBs, DNA single-strand breaks; AP, apyrimidine; dRP, deoxyribose phosphate; DSBs, DNA double-strand breaks; UV, ultraviolet; NHEJ, non-homogenous end joint; HR, homogenous recombination; 53BP1, p53-binding protein; DNA-PKcs, DNA-dependent protein kinases; XLF, XRCC4-like factor; MRN, MRE11-RAD50-NBS1; ATM, ataxic telangiectasia mutation; CHEK, checkpoint kinase; CtIP, CtBP-interacting protein; EXO1, exonuclease 1; TC-NER, transcription-coupled NER; GG-NER, global genome NER; XRCC, X-ray repair cross-complementing protein; TFIIH, transcription factor II H; PCNA; proliferating cell nuclear antigen; MMR, mismatch repair; PARP, poly-ADP-ribose polymerase; PARG, poly (ADP-ribose) glycohydrolase; PNKP, polynucleotide kinase 3'-phosphatase; APTX: aprataxin; pol, polymerase; ATRIP, ATR-interacting protein; TOPBP1: topoisomerase 2-binding protein 1; CDK: cyclin-dependent kinase; MDC1, mediator of DNA damage checkpoint protein 1; CIP2A, cellular inhibitor of PP2A; CK, casein kinase; ATR, ataxia telangiectasia and Rad3-related; RPA, Replication protein A; Me, dimethylation; P, phosphorylation; Ub, ubiquitylation; RCT, randomized clinical trial

DNA damage lesions occur in several forms, including single-strand breaks (SSBs), double-strand breaks (DSBs), insertion or deletion, mismatches, and so on [15]. Contingent upon persistent DNA damage, normal cells undergo either apoptosis or senescence as an outcome of DNA damage [16]. DNA lesions that cannot be immediately repaired generate miscellaneous mutations [17]. These mutations or the absence of adequate DDR can lead to an increased occurrence of genomic instability, impairing DNA repair capabilities and promoting the development of cancer [18]. Considering that every cell is constantly exposed to various carcinogens endogenously and exogenously that induce DNA damage, cells have developed numerous DDR pathways that enable for their survival. These various DNA damage repair mechanisms address different patterns of DNA damage in tumors as described below (Fig. 2).

### Base excision repair

SSB is the most common DNA damage that happens more than 20,000 events per cell per day caused by endogenous reactive oxygen species (ROS) or external ionizing radiation and alkylating agents [19]. SSBs are primarily repaired by the base excision repair (BER) pathway, and BER is the most adaptable excision repair mechanism overseeing the fixation of most endogenous lesions [19]. Cancer cells rely on high BER activity to combat oxidative stress [20]. BER is initially triggered by a damaged base, which is then excised and replaced with newly synthesized DNA. Apurine/apyrimidine (AP) endonuclease (APE) then cuts the AP site to form a 3’-OH terminal at the injury site [21]. Finally, DNA polymerases and DNA ligases are recruited to the nucleotide gap created by removing the damaged base, thereby sealing the gap [20]. PARP1, PARP2, and PARP3 are the key enzymes of BER [22]. As DNA damage sensors and signal transducers, PARP proteins can bind to damaged DNA at single-strand break sites to recruit DNA repair effectors [23].

BER-related proteins are crucial targets for cancer therapy. Not only PARP inhibitors have gained much attention in treating breast cancer, but also other enzymes including Polβ, APE1, and DNA ligases are potential pharmacological targets by altering the amounts of BER proteins to eliminate cancer cells [24].

### Homologous recombination

SSB belongs to ‘small’ DNA damage, while the larger damage DSB is the most cytotoxic mode among all types of DNA damage owing to challenges of precise chromosome segregations during cell division [15]. DSBs originate from exogenous sources, including chemotherapy and radiotherapy. DSBs deform the DNA’s helical structure and need to be repaired by means beyond BER, including HR, non-homologous end joining repair (NHEJ), and so on. HR, the major mechanism targeting DSBs with high fidelity, has drawn attention due to its potential therapeutic strategies [25].

**Fig. 3:**
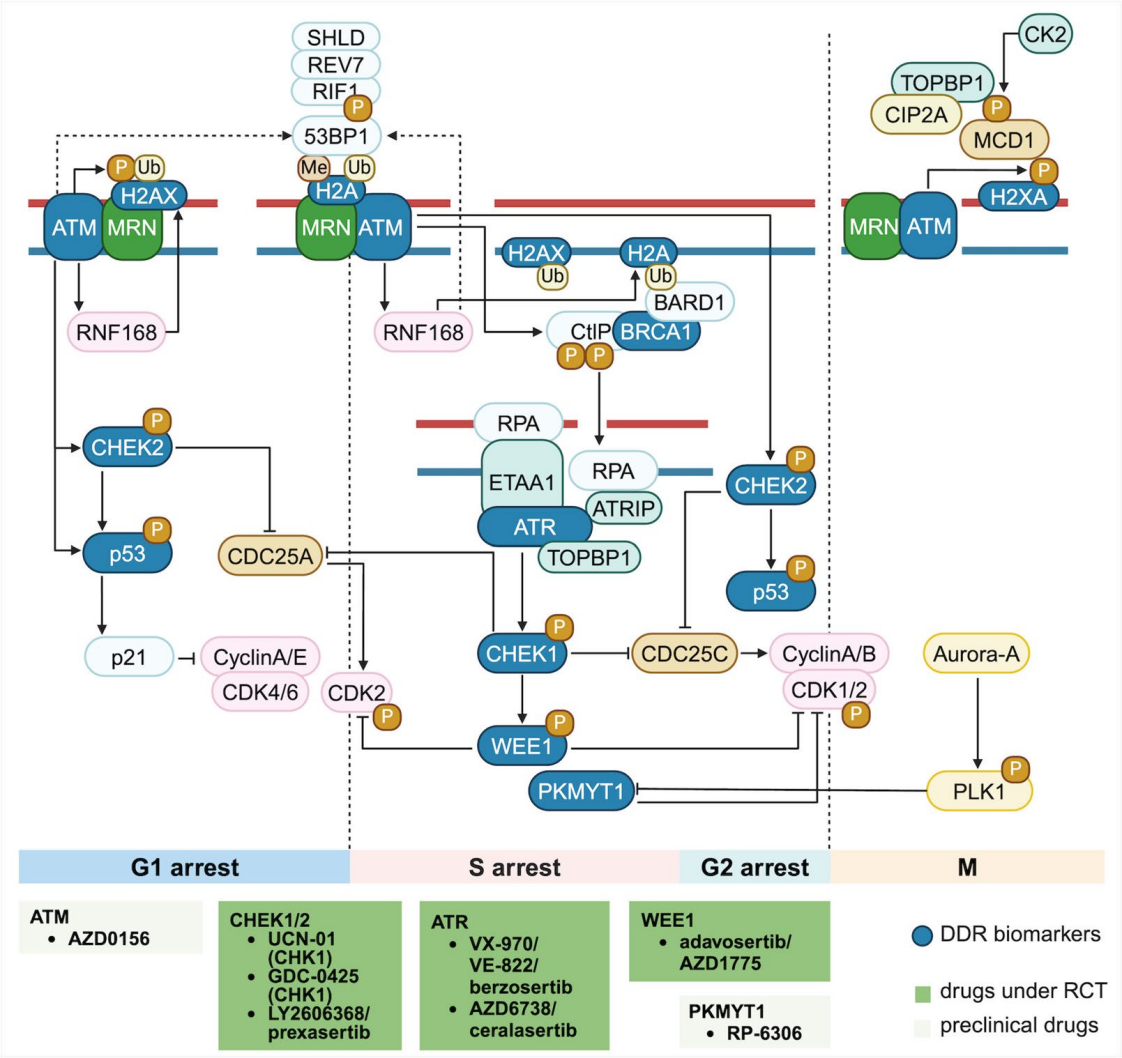
Mechanisms of cell cycle-dependent DNA damage repair activation. DNA damage checkpoints would be activated to by the presence of DNA damage, leading to cell cycle arrest to allow for DNA repair. ATM-CHEK2-p53 axis and ATR-CHEK1-WEE1 axis will be activated in response to DSBs and SSBs exposure, respectively. First, the MRN complex senses DSBs and activates ATM at break sites to orchestrate DDR signaling. During G1, ATM promotes activation of RNF168, which ubiquitylates histone H2A. This modification, together with the histone H4 Lys20 dimethylation mark results in recruitment of 53BP1 and ATM-mediated phosphorylation of 53BP1, which promotes its interaction with RIF1, REV7 and SHLD. ATM kinase activity also controls p53 stability, which triggers G1 arrest in response to DNA damage. During S phase, the BRCA1-BARD1 complex is recruited to DSBs and counteracts 53BP1 via recognition of unmethylated H4 Lys20 and H2A Lys15 ubiquitylation to promote RAD51 to load onto resected DNA ends. RPA-coated single-stranded DNA also recruits ATR via interaction with ATRIP. ETAA1 binding to RPA-coated single-stranded DNA or TOPBP1 binding to double-stranded DNA and single-stranded DNA junctions activates ATR, phosphorylating CHEK1 to promote CDC25A degradation, thus preventing activation of CDK1 and CDK2. Kinases WEE1 and PKMYT1 mediate inhibitory phosphorylation of CDK1 and CDK2, which prevents cells progressing into mitosis. In mitosis, ATM-dependent H2AX phosphorylation recruits MDC1, which binds TOPBP1 and CIP2A via its CK2-mediated phosphorylation. Aurora-A and PLK1 are implicated in mitotic entry partially through phosphorylation on WEE1 and PKMYT1 that result in their degradation. Among proteins involved in cell cycle-dependent DNA damage repair activation, ATM in G1 phase, CHEK1/2 in G1, G2 and S phase, ATR in S phase, and WEE1 and PKMYT1 in S and G2 phase can be targeted in DDR-related therapies. Drugs under RCT and in the preclinical stage are summarized in the column below (searching results on https://clinicaltrials.gov by 31st August 2024)

The process of HR is complicated and requires numerous key proteins. Initially, the Mre11-Rad50-Nbs1 (MRN) complex senses the break and binds to the DSB terminal [26]. The MRN complex recruits and activates the checkpoint protein kinases ATM (ataxia–telangiectasia mutation) and CHEK2 (checkpoint kinase 2) and phosphorylates many downstream substrates [26]. Then, the c-terminal binding protein interaction protein (CtIP) binds to BRCA1 and nucleases EXO1, further interacts with MRN and excises the DSB ends together, resulting in the formation of single-stranded DNA (ssDNA). The excised DNA ends are coated with hyperphosphorylated ssDNA -binding protein A (RPA) to form a nucleoprotein filament. The variant H2AX (γH2AX) is activated and phosphorylated by apical kinases, such as ATM and ATM- and RAD3-associated (ATR) [27]. The diffusion of γH2AX along chromosomes contributes to the recruitment and accumulation of additional DDR proteins, including p53-binding protein (53BP1) and BRCA1 to DDR lesions. Under the localization of PALB2, BRCA2 binds to BRCA1, promotes the loading of the recombinant enzyme RAD51 on ssDNA, and generates the RAD51-ssDNA-nuclear protein filament complex. RAD51 accomplishes HR by preventing the formation of secondary structures and shielding DNA ends from degradation. It mediates the invasion of homologous sequences and the formation of nuclear protein filaments and D-rings [28]. Among them, BRCA1 is involved in the initial stage of repair to initiate the removal of the 5’-end DNA strand at the damaged site and expose the 3’-end DNA single strand. BRCA2 further acts on the 3’-end single strand, promoting the formation of linking molecules between broken DNA and homologous templates, and replacing damaged or excised DNA [29]. Also, many HR-related proteins are involved in cell cycle regulation, including cell-cycle checkpoint kinase ATM and its substrate CHEK2, and cyclin-dependent kinases (CDKs) to halt cell-cycle progression in response to genotoxic stress in the G1 phase; active CHEK2 also phosphorylates p53 as well as CDC25 phosphatases resulting in S and G2 arrest [27, 30]. ATR and its substrate CHEK1 in the S and G2 phases for removing phosphorylation deposited by the kinase WEE1 and WEE1-like PKMYT1 [31] (see more details in Fig. 3).

HRD makes tumor cells highly sensitive to platinum-based drugs that induce DNA cross-linking, and the simultaneous presence of HRD and inhibition targeting other DDR pathways can lead to synthetic lethality in tumor cells [32]. Breast cancers with HRD characteristics were initially identified in more than a dozen genetic mutations associated with HRD. In addition to the most studied germline or somatic *BRCA1/2* mutations, there are also HR-related aberrations or dysfunction in “BRCAness” genes (*RAD51, CDK12, PALB2, ATM, CHEK2*, etc.), reduced epigenetic regulation of protein expression levels (*BRCA1* promoter methylation, *CDK12* promotes transcription, homozygous loss of *PTEN* and amplification of *EMSY*), and analogous microRNA miR-182 overexpression targeting *BRCA1* [14, 33–35]. All these mutations can lead to decrease and loss of function of HR protein and cause HRD.

The incidence of HRD in breast cancer could be up to 30%, among which HRD caused by HR-related genetic mutations accounts for 12 to 24% [4, 5]. Germline *BRCA1/2* mutations (g*BRCA1/2* m) are the most common and could contribute to over 20% of the HRD features in breast cancer patients [6, 36]. HRD is found in up to 50% of TNBC cases, with g*BRCA1/2* m causing 10–22% of these instances [5, 6, 36–40]. *BRCA1* mutations are more prevalent than *BRCA2* and over 40% of TNBC cases with somatic *BRCA1/2* mutations exhibit HRD positivity [11]. HRD positivity in HER2-positive breast cancer is primarily linked to *BRCA2* deletion and *BRCA1* promoter methylation [41]. Luminal breast cancer has the lowest percentage of HRD tumors [36, 42–44], and is related to *BRCA2*, *PALB2*, *ATM*, *CHEK2* mutations, inactivation of *BRCA1* by promoter methylation, and *RAD51C* promoter methylation [41].

### Non-homologous end joining repair

When tumors are HRD, DSB will be repaired in alternative error-free mechanisms, including NHEJ [33, 45]. NHEJ is simpler and quicker than HR but error-prone by directly reconnecting DSB broken ends [46]. Since homologous sister chromatids are the templates required for new DNA synthesis, HR mainly repairs DSBs in the S/G2 phase, while NHEJ operates throughout the cell cycle, especially in G0/G1, but not in the M phase [47]. In G phase, 53BP1 and RIF1 proteins localize to DSB sites, resulting in inhibition of BRCA1 recruitment, blocking DNA excision, and promoting the NHEJ repair pathway. Otherwise, the S/G2 phase stimulates DNA terminal excision and promotes HR repair. The essential components needed for NHEJ are heterodimers consisting of Ku70/Ku80 and catalytic subunits of DNA-dependent protein kinases (DNA-PKcs) [46]. These components identify DSB and facilitate the activation of downstream signaling factors of NHEJ, including XRCC4, XLF, and DNA ligase IV [48].

In breast cancer, there are NHEJ genes *XRCC5*/Ku80 and *XRCC6*/Ku70 polymorphisms associated with breast cancer risk and chromosomal radiosensitivity [49]. Nuclear KU70/KU80 expression is proven to be correlated with poor disease-free interval and prognosis, higher histological grade, lymph-vascular invasion, negative estrogen receptor expression, basal-like phenotype, P53 and CHK1 positivity [50]. Also, CD8 + lymphocyte infiltration, cancer progression, and poor survival have been linked to increased expression of DNA-PKcs, and their functions in breast cancer await further investigation [51].

### Nucleotide excision repair

NER is another important mechanism that can predominantly repair bulky adducts or intrastrand crosslinks mainly caused by UV-induced lesions or chemical alterations like cisplatin [3, 52]. The first step of NER is to recognize the damaged site. The recognition is divided into TC-NER, which repairs lesions encountered during transcription, and GG-NER regardless of transcription. Then, the TC-NER and GG-NER pathways converge, leading to nucleotide excision from the damaged strand and gap fill-in synthesis [53]. This process involves the key protein excision repairs cross complementary protein 1 (ERCC1), which removes DNA near the breakpoint and then replaces it with normal DNA replication [54]. In breast cancer, the landscape of NER-related genetic mutations varies by races; increased risks of developing breast cancer in women are found to be correlated with different ERCC1, XPC, or XPD polymorphisms in postmenopausal Chinese and American women [54–56].

In addition to HR, NHEJ, and NER, the alternative end junction (A-EJ) pathway has a similar mechanism with the two major DSB repair pathways but is genetically distinct. The A-EJ pathway can either share a similar initiation process with HR or generate DNA end junction factors without a homologous template like NHEJ [46]. At present, more research has begun to focus on the A-EJ pathway (especially its component DNA polymerase-θ, POLQ) as a potential therapeutic target for cancer cells with impaired NHEJ or HR activity [57, 58].

### Mismatch repair

In addition to the damage from cells exposed to genotoxins, DNA damage can also result from abnormal DNA processing. Mismatch repair (MMR) is responsible for identifying and repairing base pair mismatches during DNA replication, and defects in MMR by genetic mutations or epigenetic silencing can lead to an increased frequency of mutations and genomic instability [59].

MMR has been underestimated in breast cancer [59]. Only a small percentage (less than 2%) of breast carcinomas are deficient in MMR proteins [60–63], while the frequency of MMR deficiency (dMMR) in TNBC varies from 0.04% to 6.9% [64]. Despite the low prevalence of MMR defects in breast cancer, their presence has been linked to distinct clinicopathological features and different responses to treatment. Breast cancers with high histological grades, elevated Ki-67 index, and enriched tumor-infiltrating lymphocytes (TILs) characteristics tend to consist of most of the dMMR breast cancers [65–67]. Hormone receptor-negative breast cancers are associated with enriched PD-L1 expression, higher tumor mutation burden (TMB), immune-related gene expression, and aberrant expression of MMR genes compared with HR-positive tumors [67–70]. Hormone receptor-negative dMMR breast cancer treated with chemotherapy could demonstrate a better prognosis [71, 72], while Hormone receptor-positive breast cancer with dMMR status tend to possess poor outcomes in comparison of Hormone receptor-positive breast cancer with MMR sufficiency [73].

Several DNA mismatch repair gene variations may influence breast cancer susceptibility, but larger studies are needed to confirm and establish these findings. Germline mutations in *MSH6* and *PMS2* genes occur more frequently in Lynch syndrome-related breast cancer [74–78], and their polymorphisms are related to breast cancer occurrence and progression. In contrast, *MLH1* and *MSH2* mutations are more associated with sporadic breast cancer risks [79–81]. Polymorphisms in *MSH2* gene, *MSH4*, and *MUTYH* variant allele had a significantly higher risk of developing breast cancer [82–85]. However, other genotypes of the *MLH3*, *MSH3*, and *MSH6* could be linked with decreased risks [83–85]. These findings suggest that deficiencies in DNA repair pathway MMR contribute to breast cancer development.

### DNA damage tolerance

DNA damage tolerance (DDT) is a bypass mechanism for repairing replicating stalled DNA damage, allowing DNA replication to pass through obstructing elements and to complete DNA replication in the presence of DNA damage [86]. Translesion synthesis (TLS) is one of the distinct DDT modes that depend on the function of a specialized TLS DNA polymerase (such as POLK) rather than the DNA polymerase. The TLS mechanism is error-prone and increases mutation risks due to inadequate proofreading by TLS polymerase [87].

Another mode of DDT, template switching (TS), involves the recombination with homologous DNA templates on sister chromatids. This process is similar with the HR process but less accurate than TLS [87]. The repair activity of TLS and TS begins after the replication fork, indicating they occur during or after DNA replication. TS begins early in the S phase, while TLS starts late in the S phase [88]. Further research with extensive and diverse cohorts is needed to investigate the role of DDT pathway polymorphisms in breast cancer.

### Interstrand crosslink repair

DNA interstrand crosslinking (ICL) is caused by a biallelic mutation of *FANCA*, Fanconi anemia has been identified as a DNA repair pathway that removes a barrier that prevents DNA replication and transcription [89]. ICL can be formed by aldehydes during various metabolic reactions (such as lipid peroxidation and ethanol metabolism) and chemotherapy (such as platinum). Intra-strand crosslinking is repaired by the NER pathway, while ICL is mainly repaired by the FA pathway. After detecting ICL by the UHRF1 protein and the FANCM-MHF1-MHF2 complex, the FA core complex is recruited into chromatin and mono-ubiquitinated substrates FANCI and FANCD2. Ubiquitination FANCD2-I recruits scaffold proteins for various DNA endonuclides, thus completing the identification of cross-linking sites and nucleotides and obtaining DNA substrates suitable for recombinational repair [90].

Mutations in the *FANCA* gene have been proven to be linked to a higher possibility of carrying *BRCA1*/2 mutations and less response to DNA damage, which increase the risk of breast cancer [58]. *FANCA* mRNA is a potential biomarker for diagnosing breast cancer, and its overexpression is associated with the TNBC subtype, and its low expression is associated with low tumor grades [3].

#### Direct damage reversal

Direct damage reversal is a highly specialized DNA repair mechanism that directly removes DNA damage without excising or synthesizing nucleotides. It uses a special enzyme O6-methylguanine-DNA methyltransferase (MGMT) to recognize and repair DNA damage [91]. MGMT efficiently and quickly repairs specific DNA damage types, such as base mismatches and point mutations from UV radiation or alkylating agents [91]. And the MGMT gene is vital in its pathway where transferring methyl groups from damaged guanine O6 sites to cysteine residues to protect DNA from alkylating agents [92].

In breast cancer, non-conserved amino acid changes encoded by *MGMT* SNPs could significantly increase breast cancer risk [93]. In glioblastoma, methylation of the MGMT promoter, a potential predictor of therapeutic response of alkyl agents, can hinder transcription and thus be utilized to enhance cell sensitivity to alkylating agents [94]. Genes and proteins related to direct damage reversal need further demonstration on how they affect breast cancer development and treatment responses.

To sum up, there are various DDR pathways according to different types of DNA damage. SSBs can be repaired by BER, and DSBs can mainly be repaired via two mechanisms, error-free HR and error-prone NHEJ. Other damage types, such as DNA adducts, crosslinks, and oxidized bases, can be repaired using NER. When a DNA mutation, such as an insertion, deletion, or base mismatch occurs, MMR and other less common pathways including DDT are activated. In breast cancer, HR is the most important for repairing the most genotoxic damage DSB and the most studied DDR mechanism due to its indications of PARP inhibitors. Other DDR pathways, including BER, NHEJ, NER, *BRCA*-related ICL and so on, have been proven to function in breast cancer and supplement HR, but they have limited evidence on relevant interventions and clinical trials.

### Treatments targeting DDR pathways

Benefiting from diverse mechanisms of DDRs, therapies based on DNA repair pathways in breast cancer has emerged as a promising approach for providing new targets to enhance the efficacy of cancer therapy (summarized in Table 1, and the DDR relevant genetic mutation frequency in breast cancer [95–97]). In addition to the most commonly used PARP inhibitors, several drugs targeting DDR based on a synthetic lethality mechanism are in preclinical and clinical studies [9]. These targets include the protein kinases mainly responsible for DNA damage recognition and signal transduction, and participate in cell cycle regulation, such as ATM (HR) [98], ATR (HR, SSB) [99], DNA-PKcs (NHEJ) [100], CHEK1/2 (HR, SSB) [101] and WEE1 (cell cycle) [102]. The other are molecules directly involved in repairing, such as RAD51 (HR, NHEJ) [103] and POLQ (MMEJ) [104]. The inhibitors of PARP, ATR, PARG, ATR, RAD51, CHEK1, and WEE1 are studied in clinical setting, and ATG, DNA-PK, and others emerging targets are under preclinical investigations (summarized in Table 2).

**Table 1:** DDR mechanisms, their relevant genes, targeted therapies and mutation frequency in breast cancer

Mechanism	DDR-related genes	DDR-related therapies (direct inhibition or synthetic lethality)	Drugs^a^	Breast cancer frequency
**BER**	*XRCC1,2,3,4* (NHEJ)			~ 2–5% [105]
	*MUTYH*			~ 1–2% [85, 91]
**HR**	*BRCA1/2*	PARP inhibitors	olaparib, talazoparib, niraparib, rucaparib, veliparib, pamiparib, etc	5–10% [4–6, 36, 40]
	*PALB2*	PARP inhibitors		< 1% [91, 106]
	*RAD51B, RAD51C, RAD51D* (NHEJ)	RAD51 inhibitors; PARP inhibitors	B02, etc	~ 1–5% [91, 103]
	*TP53*			~ 20–50% [36, 40]
	*CHEK1, CHEK2*	CHEK1/2 inhibitors; PARP inhibitors	prexasertib (LY2606368); UCN-01; GDC-0425, etc	~ 1–4% [36, 40, 101]
	*ATM*	ATM inhibitors; PARP inhibitors	AZD0156, etc	2 ~ 5% [36, 40, 98]
	*ATR*	ATR inhibitors; PARP inhibitors	VX-970 (VE-822 or berzosertib); ceralasertib (AZD6738); ART6043, etc	~ 5% [36, 40, 99, 101]
	*WRN*	PD-1/PD-L1 inhibitors		< 1% [36, 40]
**NHEJ**	*KU70, XRCC4*			< 1% [105]
**MMR**	*MLH1, MSH2, MSH6, PMS2*	PD-1/PD-L1 inhibitors	pembrolizumab; atezolizumab; durvalumab, etc	< 2% [60–63, 85, 91]
**ICL**	*FANC* genes			~ 1–2% [58, 91]
**Direct damage reversal**	*MGMT*			< 1% [91, 93]
**Cell cycle regulations**	*WEE1*	WEE1 inhibitors; PARP inhibitors	adavosertib (AZD1775), etc	< 1% [91, 102]
**PI3K pathway**	*PTEN*	PARP inhibitors; RAD51 inhibitors		~ 5–15% [36, 40, 91]

*DDR* DNA damage response, *BER* base excision repair, *HR* homologous recombination, *ICL* interstrand crosslinking, *NHEJ* non-homologous end junction, *MMR* mismatch repair

^a^Drugs searched on https://clinicaltrials.gov by 31st August 2024

**Table 2:** DDR-related therapies^a^ in addition to PARP inhibitors in breast cancer

Target	DDR mechanisms	Agent	Regimens in clinical trials	Breast cancer indications
**ATM**	**HR**: H2AX phosphorylation, checkpoint signaling	AZD0156	monotherapy	NA
			combined with olaparib	NA
			combined with chemotherapy	NA
**ATR**	**SSB, DSB**: stabilize replication folk; with downstream CHK1 molecules, cell cycle capture	VX-970 (VE-822 or berzosertib)	monotherapy	metastatic TNBC
			combined with chemotherapy	metastatic TNBC; advanced breast cancer;
			combined with radiotherapy	chemotherapy-resistant TNBC; HER2-negative breast cancer
		ceralasertib (AZD6738)	monotherapy (neoadjuvant)	(metastatic) TNBC
			combined with olaparib	advanced/metastatic; chemotherapy-resistant; with(out) HR-related gene mutations TNBC
			combined with immunotherapy	advanced/metastatic TNBC
			combined with chemotherapy	advanced/metastatic TNBC
		ART6043	monotherapy	advanced/metastatic HER2-negative *BRCA*m breast cancer
**CHEK1/2**	**SSB, DSB**: ATR downstream kinase	prexasertib (LY2606368)	monotherapy	*BRCA1/2* m advanced breast cancer; non-*BRCA1/2* m advanced TNBC
			combined with olaparib	advanced breast cancer
			combined with chemotherapy	advanced/metastatic TNBC
			combined with radiotherapy	chemotherapy-resistant TNBC
			combined with targeted therapy	NA
			combined with immunotherapy	NA
			combined with DNA-PK inhibitor	metastatic TNBC
		UCN-01	monotherapy	advanced/metastatic TNBC
		GDC-0425	monotherapy	advanced/metastatic
**DNA-PK**	**NHEJ**: bind DNA end to ATR, ATM; activate checkpoints phosphorylation			NA
**PARG**	**BER**: reverses PARP action	IDE-161	monotherapy	advanced g*BRCA1/2* m breast cancer
**RAD51**	**HR, NHEJ**: signal strand invasion and subsequent homologous strand exchange			NA
**WEE**	tyrosine kinase of G2/M/S phase checkpoint; inhibit CDK1 phosphorylation	adavosertib (AZD1775)	monotherapy	advanced/metastatic TNBC
			combined with olaparib	metastatic stage II-III TNBC or (with HR-related gene mutations)
			combined with chemotherapy	metastatic stage II-III TNBC or (with HR-related gene mutations)

*DDR* DNA damage response, *SSB* single-strand DNA break, *DSB* double-strand DNA break, *HR* homogenous recombination, *NHEJ* non-homologous end junction, *BER* base excision repair, *TNBC* triple negative breast cancer, *HER2* Human Epidermal Growth Factor Receptor 2, *BRCAm BRCA1/2* mutation, *gBRCA1/2 m* germline *BRCA1/2* mutation, *NA* not available

^a^Drugs searched on https://clinicaltrials.gov by 31st August 2024

### PARP inhibitors

PARP inhibitors are the first agents designed to interfere with the ability of DNA repair [107]. Given the critical role of PARP inhibitors in selectively killing tumor cells with HRD features, they have emerged as a promising therapeutic strategy whose indications have been expanded from germline *BRCA1/2*-mutated to HRD breast cancer [108]. PARP is a well-recognized DNA damage sensor that is mainly responsible for detecting SSBs [109]. When PARP is inhibited, SSB accumulates and transforms DSBs. Cells with wild-type *BRCA1/2* repair DSBs through HR, whereas in cells with *BRCA1/2* mutations or HRD, accessory DNA repair pathways are also inhibited by trapping PARP1 and PARP2 at the DNA damage location. This synthetic lethality makes HRD tumors vulnerable to PARP inhibitors, resulting in tumor cell death [110].

There are several PARP inhibitors undergoing clinical trials since the first admission of olaparib in 2014. Olaparib was the first approved by the FDA for treating g*BRCA1/2* m, HER2-negative metastatic breast cancers [111]. PARP inhibitors usually follow the completion of chemotherapy and radiation therapy, and can also be administered along with hormone therapy, or adapts to the neoadjuvant setting.

### PARP inhibitor monotherapy

PARP inhibitors alone function well in breast cancer with DDR deficiency, especially with a focus on the refractory subtype TNBC (summarized in Supplementary Table 1). A phase 2 study in which patients with advanced g*BRCA1/2* m breast cancer were administered various doses of olaparib monotherapy revealed that TNBC appeared to derive greater clinical benefits compared to non-TNBC, and higher doses were associated with higher objective response rates (ORR) [12]. In another non-randomized phase 2 study, objective responses were not observed in advanced TNBC patients without g*BRCA1/2* m receiving olaparib alone. Although the lack of response might be related to the limited sample size and heavy pretreatment, lack of *BRCA1/2* mutations might be the primary reason [13]. The OlympiAD study, a phase 3 trial comparing olaparib alone to treatment chosen by physicians in *BRCA1/2* m metastatic TNBC, demonstrated that patients receiving olaparib had a significantly longer median progression-free survival (mPFS) with an increase of 3 months. However, there was only a numerical improvement in median overall survival (mOS) without statistical significance [112]. These findings were consistent with results seen in the broader population of g*BRCA1/2* m and HER2-negative metastatic breast cancer [113].

Talazoparib, a second-generation PARP inhibitor, is the second inhibitor approved by the FDA for g*BRCA1/*2 m, HER2-negative, advanced breast cancer [114]. Talazoparib can help to slow the growth of cancer cells and increase survival rates for individuals with metastasis or recurrences [115–117]. The TBB phase 2 trial has expanded its monotherapy indication beyond *BRCA1/2* mutation to breast cancer with other HR-related genetic mutations [118]. The EMBRACA trial, a phase 3 study involving advanced TNBC with g*BRCA1/2* m suggested that talazoparib monotherapy favored longer PFS than single-agent chemotherapy [119]. In addition to the advanced setting, studies investigating PARP inhibitors as neoadjuvant treatments have been ongoing. A recent pilot trial suggested that neoadjuvant single-agent talazoparib induced residual cancer burden of 63% in g*BRCA1*-mutated breast cancer patients and 57% in g*BRCA1*m TNBC patients [120].

Other PARP inhibitors are under research but with limited clinical evidence. Rucaparib may benefit a limited subset of patients with high LOH scores regardless of inherited *BRCA1/2* mutations [121]. The RUBY trial on advanced *BRCA*m breast cancer highlights the importance of developing new biomarkers to identify potential responders more accurately [121]. Niraparib experienced termination of recruitment due to a significant discrepancy between improved local PFS and OS, ORR [122], and lack of improvement in central PFS when combined with pembrolizumab therapy in advanced or metastatic TNBC [123].

The clinical efficacy of PARP inhibitor monotherapy in advanced and metastatic breast cancer with g*BRCA1/2* m is well-established, and promising results are also observed in HR-positive tumors. Whether early TNBC or other subtypes will benefit from these treatments is worth investigating further. PARP inhibitors have an acceptable safety profile, whose common grade 3/4 adverse events are anemia, neutropenia, fatigue, and nausea, and studies have shown tolerate safety profiles [113, 120].

However, the emergence of resistance to PARP inhibitors due to drug target-related effects limits their clinical efficacy [31]. Because PARP trapping mediates the anti-tumor activity of PARP inhibitors and acquired resistance [124, 125], novel PARP inhibitors that are not substrates for the former drug efflux with increased PARP trapping capacity, such as the recently developed veliparib derivative, are promising clinical candidates, especially for patients who have undergone chemotherapy [126, 127]. Veliparib was first studied in ovarian cancer and targets PARP1 and PARP2 enzymes. It is under clinical trials on g*BRCA1/2* m metastatic breast cancer and compared with platinum-based chemotherapy [128, 129]. Preclinical studies on pamiparib have also shown antitumor activity in cell lines, and it is also under research on breast cancer, especially in patients with *BRCA* mutations or other DDR pathway defects [130, 131].

Despite PARP inhibitors’ remarkable success in treating breast cancer, not all breast cancer patients with DDR deficiency do not respond to PARP inhibitor treatment alone [3]. In addition, patients who initially responded to the treatment could acquire PARP inhibitor resistance after prolonged use [132]. Mechanisms of resistance include drug target-related effects including the upregulation of drug efflux pumps [125], decrease of PARP capture ability [133], *BRCA1* demethylation [134] and *P53B1* silencing [135], BRCA1/2 function recovery or non-BRCA1-dependent HR function recovery [136, 137], and restoration of stability in DNA end protection or replication forks [138]. Therefore, the synergistic effect of PARP inhibitors combined with other therapies has encouraged the design of DDR inhibitor-based combination treatments in view of its drug resistance [132].

### PARP inhibitors with chemotherapy

Current promising drug combinations include PARP inhibitors in combination with chemotherapy, radiotherapy, and immunotherapy. Clinical trials on PARP inhibitor in combination with other targeted or DDR-related drugs are also ongoing, including anti-angiogenic agents, PI3K/AKT pathway inhibitors, the RAS/RAF/MEK/MAPK pathway inhibitors, ATR/WEE1 inhibitor and epigenetic modification [3, 15].

The combination strategies of PARP inhibitors with (neo)adjuvant chemotherapy regimens in breast cancer have been also investigated for decades (summarized in Supplementary Table 2). The genomic instability induced by mutations in HR-related genes potentially renders cancer cells susceptible to chemotherapeutic drugs, particularly those that directly damage DNA, such as anthracycline or platinum-based drugs. Platinum-induced DNA interstrand crosslinks require functional HR for repair, and therefore, breast cancer with HRD is likely to be sensitive to platinum salts [139]. This has been proven in the GeparSixto study, where HRD-positive breast cancer showed a higher pathological complete response (pCR) rate in the paclitaxel combined with liposomal adriamycin group versus paclitaxel combined with liposomal adriamycin plus carboplatin group [140].

In the PARP inhibitors and chemotherapy combined settings, it seems that the benefits of PARP inhibitors in combination with platinum salts for breast cancer are relatively limited. In the HRD-positive, HER2-negative breast cancer in the GeparOLA study, olaparib with platinum performed better outcomes compared to standard neoadjuvant platinum chemotherapy in pCR with ORR of 80% and tolerable safety [141]. The phase 2 I-SPY 2 trail showed the addition of the veliparib-carboplatin regimen to standard neoadjuvant chemotherapy among stage II/III TNBC patients increased the estimated pCR from 51 to 26% [142]. Further investigation demonstrated that the *BRCA1* mutation was significantly associated with the response to combined therapy of veliparib and carboplatin [143]. However, the phase 3 BrighTNess in which veliparib was added to paclitaxel and carboplatin followed by doxorubicin and cyclophosphamide in operable stage II/III TNBC patients, showed no additional benefit of pCR adding PARP inhibition [144]. The phase 2 BROCADE study, which involved adding veliparib to carboplatin and paclitaxel for g*BRCA1/2* m breast cancer, showed no improved mPFS in the TNBC subtype [145]. The results of a subsequent phase 3 BROCADE3 demonstrated a numerical increase in mPFS in g*BRCA1/2* m advanced TNBC, but this increase was not statistically significant [146]. The Hoosier Oncology BRE09-146 phase 2 trial also demonstrated that adding another PARP1 inhibitor rucaparib to cisplatin did not improve the 2-year DFS benefits in TNBC or g*BRCA1/2* m breast cancer with residual disease post-neoadjuvant therapy [147].

In addition to platinum salts, exploratory studies have investigated the combination treatments of PARP inhibitors with other chemotherapeutic drugs, showing improved responses in HRD-positive patients. Combined treatment with olaparib and paclitaxel or eribulin showed antitumor activity in metastatic TNBC patients in phase 1/2 clinical trials, but serious neutropenia might hinder their applications in clinical practice [148, 149]. Another phase 2 trial demonstrated that a low dose of veliparib combined with cyclophosphamide treatment did not improve the clinical response in refractory TNBC [150].

### Targeting HRD to enhance breast *cancer* immunogenicity

HRD is associated with tumor immunity. On the one hand, tumors with deficient DDR were inclined to have higher genomic instability, and therefore, were associated with a higher neoantigen enrichment or TMB, which might increase their sensitivity to immune checkpoint inhibitors [151]. There is a synergistic effect of PARP inhibitors and PD-L1/PD-1 blockade against breast cancer [152]. On the other hand, HRD features are also associated with up-regulation of chemokines and immune checkpoint genes and create an inflammatory but immunosuppressive tumor microenvironment.

**Fig. 4:**
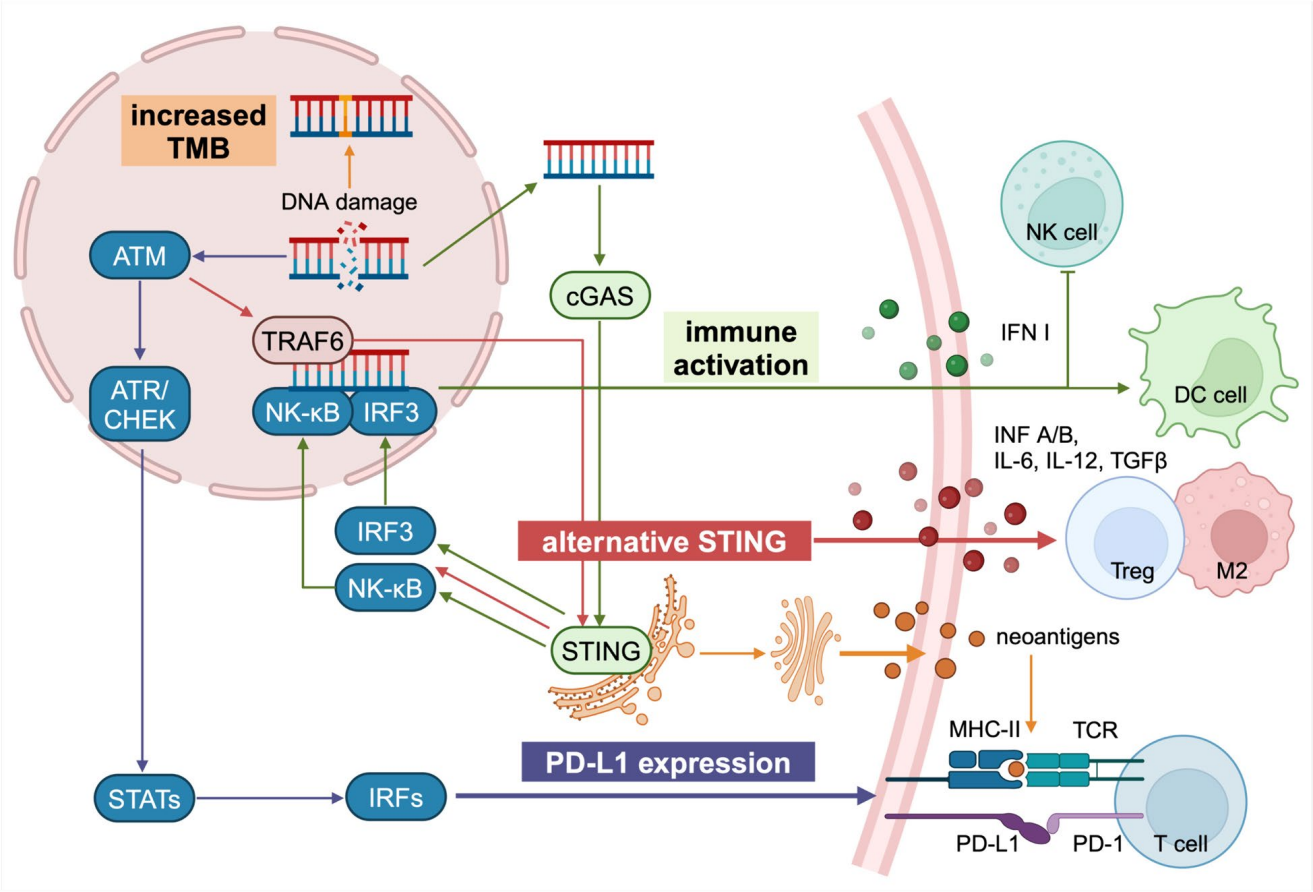
The impact of DNA damage on immune tumor microenvironment. Immune tumor microenvironment can adapt to DNA damage in various manners. The STING pathway can be activated by increased TMB, the upstream cytosolic DNA sensor cGAS or ATM and TRAF6 after perceiving DNA damaging agents or DDR alterations, leading to immune activation. This activation makes a conformation change of STING, which leads to an endoplasmic reticulum to perinuclear endosome shuttling. The activated STING stimulates IRF3, which promotes the production of type I IFNs. Consequently, NK cells are inactivated, while T cells and dendrite cells are activated. STING also activates NK-κB by ATM-TRAF6, induces IL, TGF-β, and other cytokine production, further recruits Tregs and M2-like macrophages. DDR deficiencies also improve tumor recognition through generating neoantigens. Additionally, DNA damage signaling and DDR deficiencies role as important regulators in upregulating PD-L1 expression. ATM-TRAF6 activates NF-kB and induces tumor cell upregulation of PD-L1 that may elicit immune escape. Besides this mechanism, IFN I itself (secreted upon STING activation) is the main factor inducing transcription and expression of PD-L1. DDR, DNA damage response; STING, stimulator of interferon genes; TMB, tumor mutation burden; cGAS, cyclic GMPeAMP synthase; IRF3, interferon regulatory factor 3; NK, natural kill; NK-κB, transcription factor nuclear factor κB; TGF-β, transforming growth factor (TGF)-β; IFN, interferon; Treg, regulatory T cells; PD-L1, programmed cell death-ligand 1

The use of PARP inhibitors will cause DNA damage, and a large amount of dsDNA will be produced in HRD status. dsDNA in the cytoplasm can bind with cyclic GMP-AMP synthase (cGAS) to form cyclic GMP-AMP (cGAMP). Subsequently, Stimulator of Interferon Genes (STING) binds with cGAMP to facilitate IRF3 phosphorylation and nuclear translocation, leading to the expression of interferons, particularly type I interferon. At the same time, it can kill tumor cells under the action of cytokines and recruited T cells. The entry of dsDNA into the cytoplasm activates the cGAS-STING pathway, and the up-regulation of PD-L1 through the STING pathway leads to T cell exhaustion. Moreover, stromal TILs are associated with DNA damage immune response (DDIR), and a high DDIR score is associated with a good prognosis for early TNBC [153]. To conclude, PARP inhibitors single strand break repair and increases DNA damage, inducing accumulation of DSBs that HRD cells cannot repair thus possibly leading to a higher TMB and neo-antigens expression [107]. This mechanism suggests the rationality of PARP inhibitors combined with immune checkpoint inhibitors (summarized in Fig. 4).

Combining immunotherapy with PARP inhibitors provides a new perspective for treating TNBC and clinical studies of PARP inhibitors combined with immunotherapy (mostly PD-1/PD-L1 inhibitors) have achieved initial results (summarized in Supplementary Table 3) [108]. TOPACIO/KEYNOTE-162, the first study evaluating the safety and efficacy of PARP inhibitors with immunotherapy, showed that niraparib and pembrolizumab in metastatic TNBC patients irrespective of *BRCA1/2* mutation status or PD-L1 expression yielded an ORR of 21% and well-tolerable safety profile. Patients with *BRCA1/2* mutations had a superior response of ORR and mPFS [123]. Another recent phase 1/2 study, the MEDIOLA trial, explored the efficacy and safety of olaparib combined with the PD-L1 inhibitor durvalumab in HER2-negative g*BRCA1/2* m breast cancer population. The survival benefits for TNBC were comparable to other breast cancer subtypes [154]. The phase 2 MEDIOLA study is underway, targeting different populations (*BRCA1/2* mutations, other HR-related gene mutations, and TNBC) to further investigate and expand the advantages of PARP inhibitor combined immunotherapy. KEYLYNK-009 for the *BRCA* somatic mutation population, after induction chemotherapy, olaparib combined immunotherapy group compared with chemotherapy combined immunotherapy group extended the mPFS [155]. In addition to the benefits of combining immunotherapy with PARP inhibitors, more effective therapies are still required for *BRCA1/2*-negative and PD-1/L1-negative breast cancer, as well as non-TNBC subtypes and in the neoadjuvant setting.

### Radiosensitization effects of PARP inhibitors

PARP inhibitors in combination with radiotherapy are being evaluated in clinical trials, especially in TNBC (summarized in Supplementary Table 4). The underlying mechanism could be PARP inhibition or knockdown can reverse the radioresistance phenotype, suggesting that PARP could be a promising therapeutic target [156]. The preliminary results of the completed RADIOPARP phase 1 trial demonstrated that the recommended dose was olaparib 200 mg twice a day combined with locoregional radiotherapy [157]. Other trials with various PARP inhibitors in combination with radiotherapy are ongoing or recruiting, and clinical studies are needed to further validate the radiosensitization effects of PARP inhibitors. It is important to note that ATM or ATR inhibition also has similar radiosensitization effects [158, 159], and a related clinical trial has begun (NCT04052555).

### PARP inhibitors with other targeted treatments

Numerous studies have observed that targeted treatment strategies could expand the potency and applicability of PARP1 inhibitors. Vascular endothelial growth factor receptor (VEGFR) inhibitors (cediranib, apatinib), PI3K inhibitors (BKM120, BYL719, CYH33), mTOR inhibitors (vistusertib), CHEK1/2 inhibitors (prexasertib or LY2606368), WEE1 inhibitors (adavosertib), and so on were reported to suppress HR and lead to synergistic effects against TNBC cells by downregulating HR-related proteins or impeding the recruitment of HR protein to the DSB site [34, 160–165]. Several of these agents are being investigated on the synergistic efficacy in combination with PARP inhibitors in clinical trials (summarized in Supplementary Table 5).

### ATM inhibitors

ATM kinase senses DSBs and phosphorylates histone H2AX on serine 139, yielding γ-H2AX foci, and recruits DNA damage mediator proteins to the DSB sites [7]. Loss of the ATM signal delays terminal excision and repairs the single-ended DSB via NHEJ, resulting in abnormal chromosome fusion and further death of tumor cells [166]. Germline *ATM* mutations predispose patients to familial breast cancer and are associated with HRD in *BRCA1/2*-wild type breast cancer patients [167, 168]. AZD0156 is a potent and selective inhibitor of ATM with strongly improved pharmacokinetic properties, allowing for its in vivo application. Patient-derived TNBC xenograft models showed that AZD0156 enhanced the response to olaparib treatment by inhibiting the repair of olaparib-induced DNA damage [169].

Moreover, ATM inhibitors help enhance the sensitivity of radiotherapy, and radioresistant breast cancer has enhanced DDR efficiency and increased expression of ATM [159]. Increased ATM expression contributes to radioresistance development, and ATM inhibition or knockdown reverse this phenotype, making ATM a promising therapeutic target for treatment. It is also worth noting that long-term exposure to ATM inhibitors may lead to neoplastic formation [98]. This could explain their immature application in breast cancer and special concerns should be given to ensure that the therapeutic benefits of ATM inhibitors outweigh the therapeutic risks.

### ATR inhibitors

ATR is activated and recruited by ssDNA wrapped in RPA. Intracellular ATR signaling involves phosphorylation of downstream molecules, triggering a wide range of responses including blocking cell cycle checkpoints, DDR, and apoptosis. In the S phase, ATR ensures timely and accurate DNA replication by regulating the initial ignition and bifurcation process. ATR inhibitors increase replication fork arrest and promote chromosome breakage [99]. And the most promising clinical applications of ATR inhibitors is the potential to treat PARP inhibitor-resistant tumors. *BRCA1* mutations can overcome the toxicity of PARP inhibitors by loading BRCA1-independent RAD51 on DSBs, leading to the development of resistance. ATR inhibitors block this BRCA1-independent function, making tumor cells sensitive to reactivation of PARP inhibition [170].

ATR protein levels are down-regulated in breast cancer and high ATR protein levels are associated with higher tumor stage and lymph vascular invasion [101, 171]. VX-970 (VE-822 or berzosertib) is a potent and selective ATR inhibitor and was the first ATR inhibitor tested in clinical trials [101, 172]. VX-970 monotherapy did not show significant activity in TNBC models, even those with p53 mutations or MYC and cyclin E2 amplification [158]. A phase I clinical trial evaluating the safety and efficacy of VX-970 monotherapy or in combination with chemotherapy is ongoing (NCT02157792), and partial response (PR) was observed in one breast cancer patients [173–175]. Preliminary data from another cohort in which 35 metastatic TNBC patients were given VX-970 and cisplatin showed that the ORR 38.9% and mPFS 4.1 months [176]. VX-970 also exhibited pronounced radiosensitization effects in TNBC PDX models, even in chemoresistant biopsy specimens [158]. A related clinical trial to test the addition of VX-970 to radiation therapy for chemotherapy-resistant TNBC is ongoing (NCT04052555). Another ongoing phase 1/2 trial also focuses on ATR inhibitors ART6043 monotherapy in advanced or metastatic HER2-negative *BRCA*m breast cancer (NCT05898399).

AZD6738 (ceralasertib), a selective small-molecule ATR inhibitor, was recently introduced as the second ATR inhibitor in clinical trials. Preliminary data showed that AZD6738 monotherapy had an IC50 of less than 1 μM in diverse cancer cell lines, including TNBC cells. AZD6738 alone showed a more significant anti-tumor effect in *BRCA1*m TNBC, suggesting *BRCA1*m as a potential predictive biomarker and PARP inhibitor as a possible synergistic drug [177, 178]. In addition, AZD6738 could potentiate the efficacy of the WEE1 inhibitor AZD1775 in TNBC cells by inhibiting AZD1775-induced DDR activation and causing DNA damage, replication stress, and mitotic catastrophe [177]. The initial result of a phase 1 clinical study in which AZD6738 was given with olaparib showed that two BRCA-mutated TNBC patients achieved a PR, but the exact clinical response rate requires updated data [179]. The VIOLETTE study, a multicenter ongoing phase 2 study (NCT03330847), olaparib given along with AZD6738 to advanced TNBC fail to reveal a superior efficacy of the combined regimens with no statistically significant difference in PFS and ORR [180].

Generally, ATR inhibitor monotherapy may be effective only in TNBC tumors with specific molecular backgrounds. The preliminary results suggest that ATR inhibitors combined with chemotherapy (NCT02157792, VX-970 + carboplatin), radiotherapy (NCT04052555, berzosertib + radiotherapy), and immunotherapy (NCT03740893, (neo)adjuvant olaparib + durvalumab + AZD6738) show excellent antitumor activity [158, 174, 175]. Their clinical applications still need to be investigated in large-scale clinical trials. Compared to other DDR proteins such as PARP, the development of ATR inhibitors is lagging behind due to large size of ATR molecules and lack of understanding of their crystal structure. In addition, its high homologous active sites in all PIKKs and the need for coactivated proteins further limit its drug design [99].

### CHEK1/2 inhibitors

Small molecule inhibitors in addition to PARP (with HRD) targeting DDR, such as CHEK1/2 (or CHK1/2), RAD51, and DNA-PK also function with the help of synthetic lethality with *BRCA* and other DDR pathway deficiency [181, 182]. Among them, CHEK1 is expressed at higher levels in breast tumors, especially in TNBC, and its deregulated level also surpasses that of its upstream kinases ATR and ATM [183, 184]. CHEK1 inhibitors selectively kill cancer cells with high levels of replication stress [185]. Similar to ATR inhibitors, CHEK1 inhibitors exacerbate DNA damage caused by PARP inhibitors [186]. High pCHEK1Ser345 levels are associated with DDR deficiency, such as low BRCA1 and DNA-PKcs expression [101]. Preclinical data have shown the efficacy of ATP-competitive dual CHEK1/2 inhibitors in TNBC, including AZD7762 [187] with enhanced sensitivity due to increased DNA replication stress induced by RB loss and V158411 [188]. UCN-01 and V158411 demonstrated robust potentiation when combined with gemcitabine or cisplatin compared to several other chemotherapeutic agents [188, 189].

UCN-01 is the first defined CHEK1 inhibitor, and the combination of UCN-01 and gemcitabine resulted in a synergistic inhibitory effect on TNBC because UCN-01 drove the cells arrested in the S-phase induced by gemcitabine through the G2/M checkpoint and caused increased DNA damage, leading to cell death [189]. Preclinical data also showed that UCN-01 enhanced the antitumor effects of irinotecan in P53-mutated TNBC. In a phase I clinical trial investigating UCN-01 and irinotecan in refractory advanced solid tumors, 2 of 5 TNBC patients achieved partial response (PR) [190]. However, the data from a phase 2 clinical trial investigating UCN-01 and irinotecan in metastatic TNBC were not optimistic; only 1 metastatic TNBC patient reached PR. The limited efficacy of UCN-01 might be related to the nonspecific inhibition of CHEK1 and the limited tissue bioavailability of UCN-01 [191].

Another phase 1 study of GDC-0425, a new selective small-molecule inhibitor of CHEK1, with gemcitabine demonstrated that 2 of 5 TNBC patients reached PR, and tumor biopsies showed that GDC-0425 administration decreased the pCDK1/2 expression induced by gemcitabine, in accordance with the checkpoint override caused by CHEK1 inhibitors [192].

LY2606368 (prexasertib) is a second-generation CHEK1/2 dual inhibitor preferentially inhibiting CHEK1. LY2606368 monotherapy was tolerable and clinically effective in patients with high-grade serous ovarian carcinoma [193], which shares common characteristics with TNBC. LY2606368 was extremely effective as a monotherapy against TNBC cells and PDX models [194]. Further biomarker analysis suggested that TNBC cells with a higher phosphorylation level of DNA-PKcs or RPA32 and TNBC PDX tumors with a higher mRNA expression level of cyclin E1, cyclin D1, and MYC showed higher sensitivity [194, 195]. Prexasertib has showed moderate clinical efficacy in a phase 2 trial on *BRCA1/2*-mutated breast cancer and sporadic TNBC is ongoing; 1 of 9 patients reached PR and 4 of 9 attained stable disease [196, 197]. Furthermore, LY2606368 was recently demonstrated to potentiate the cytotoxicity of the PARP inhibitor olaparib in *BRCA*-proficient TNBC by decreasing the expression of the HRR proteins BRCA1 and RAD51 in a proteasome-dependent manner [198]. A clinical trial of combination therapy of LY2606368 and olaparib is currently ongoing (NCT03057145). In addition, prexasertib was proven to activate type I interferon signaling and, in combination with anti-PD-1 immunotherapy, produced a synergistic anti-tumor response in a mouse model of small cell lung cancer, indicating its potential of combining immunotherapy [199].

In all, CHEK1 inhibitors have been extensively studied in TNBC. Phosphorylation levels of DNA-PKcs, RPA32, CHEK1 autophosphorylation, and so on are potential biomarkers of its efficacy but need further validation in clinical trials. More data about the potential role of CHEK1 inhibitor monotherapy and in combination with chemotherapy in TNBC patients is eagerly anticipated.

### DNA-PK inhibitors

DNA-PK, a key protein kinase in DNA repair, binds to the end of DNA to activate the repair process and participates in NHEJ [27]. DNA-PK works in conjunction with ATR and ATM to activate the phosphorylation of proteins involved in DNA damage checkpoints [200]. Upregulated DNA-PK expression has been observed in breast cancer and is associated with higher tumor grade and poor prognosis [201]. Cells lacking DNA-PK are sensitive to DSB inducers and medication of DNA-PK inhibitors in breast cancer is still in preclinical experiments [200]. The development of DNA-PK inhibitors has focused on the catalytic activity of DNA-PKcs. New anti-DNA-PKcs approaches, such as DNA-PKcs inhibitory microRNAs or inhibitors targeting Ku heterodimers, are based on homologous models of ATP-binding sites [15]. Currently, most studies on DNA-PK inhibitors focus on their effects when used in combination with cancer chemotherapy or radiation [16]. The combined inactivation of DNA-PK and BRCA1 also leads to synthetic lethality, indicating the possibility of their combined treatment [200]. Among them, NU7441 could reduce ionizing radiation-induced activity, as well as downregulate DSB repair and greatly improved the sensitivity of all cell lines to ionizing radiation and doxorubicin [15, 202]. This suggests that DNA-PK may be an effective target for chemo- and radio-potentiation in breast cancer. Another DNA-PK inhibitor, AZD7648, in combination with olaparib, also leads to breast cell death in vivo [201, 203]. ATM and DNA-PKcs are also synthetically lethal, so DNA-PKcs inhibitors may also be effective against tumors with *ATM* mutations [204]. More evidence in vitro is needed to further prove the efficacy of DNA-PK inhibitors in breast cancer.

### PARG inhibitors

PARG reverses the action of PARP enzymes by hydrolyzing PAR ribose bonds after DNA damage [205]. Similarly, the positive role of PARG in DNA replication and repair leads to increased sensitivity of PARG-deficient cells to DNA-damaging agents [206]. Loss of expression of PARG, the enzyme responsible for the degradation of poly(ADP-ribose) chains, stabilizes poly(ADP-ribosyl) ation and promotes PARP inhibitor resistance in BRCA1/2-deficient tumors [207].

Although numerous studies have shown a correlation between PARP inhibitors and synthetic mortality, research into the therapeutic mechanisms of PARG inhibitors has lagged behind. Medication of PARG inhibitor in breast cancer is mainly under preclinical investigation. Experiments have showed that loss of HR proteins such as BRCA1/2 in breast cancer cells stimulate the synthetic lethality of PARG-inhibiting cells, and the PARG inhibitor induces cell death of *BRCA* mutated or olaparib-resistant breast cancer cells [207]. The ongoing phase 1 clinical trials (NCT05787587) focuses on the efficacy of PARG inhibitor IDE-161 in advanced g*BRCA1/2* m BC.

### RAD51 inhibitors

RAD51 protein can form a nuclear focus required for functional homologous recombination in response to DNA damage. The presence of RAD51 in S-phase cycling cells indicates a functional HR pathway, whereas the absence of RAD51 foci reveals HRD [208].

RAD51 protein is an essential enzyme in the pathway repairing DSBs. At the DSB sites, RAD51 will eventually signal strand invasion and subsequent homologous strand exchange for successful damage repair [209]. RAD51 assembles nuclear protein filaments on ssDNA formed on the excised DSB, thereby catalyze the chain’s invasion into the homologous DNA template to initiate HR repair. Pathogenic RAD51C variants contribute to breast cancer risk, and elevated RAD51 levels in cell lines and primary tumors suggest that driving the HR pathway beyond physiological levels may also play a role in genomic instability and hence tumor progression [14, 210]. BRCA2 is the loader of RAD51 at the DSB site, and the removal of RAD51 and the inhibition of PARP form a synthetic lethal effect [209]. Furthermore, the tumor suppressor gene *PTEN* deficiency leads to RAD51 downregulation [211]. The PTEN protein promotes DNA repair through RAD51-dependent homologous recombination, and PTEN-deficient breast cancer cells are sensitive to olaparib, a RI-1 inhibitor. RI-1 inhibits RAD51-mediated DNA strand exchange and sensitizes cancer cells to DNA damage-inducing agents, thus increases cancer cell death [210, 212]. B02 is a small molecule that binds to the RAD51 protein and blocks its activity. Preclinical studies have demonstrated that combining B02 with cisplatin has the most potent effect on cancer cells, thereby resulting in enhanced anti-cancer activity compared to either drug alone. In vivo studies have also shown that B02 greatly improves the therapeutic efficacy of cisplatin on tumor cells, further supporting the potential of RAD51-specific small molecule inhibitors as a viable approach for combination anti-cancer therapy [213].

### WEE1 inhibitors

Tyrosine kinase WEE1 regulates the G2/M checkpoint and the replication origin firing in the S phase. WEE1 is also responsible for the inhibitory phosphorylation of CDK1, thereby preventing cells from entering mitosis [214]. WEE1 inhibition enhanced replication stress and DNA damage. Premature mitosis is its critical mechanism against tumors, inducing highly abnormal mitosis, with subsequent mitotic catastrophe, ultimately resulting in apoptosis or long-term cell cycle arrest [215].

AZD1775 (adavosertib) is the most frequently tested and is the only one in clinical trials to date [216]. In a recent study, the basal-like breast cancer subtype exhibited the highest sensitivity to AZD1775 monotherapy [217]. Global proteome profiling identified that low PTEN expression was strongly associated with sensitivity to AZD1775, and siRNA screen showed that DNA-PK inhibition sensitized BLBC cells to AZD1775, and cyclin E overexpression via genomic transcription analyses had high sensitivity to AZD1775 alone in vitro and in PDX models, making them potential biomarkers for predicting the response to WEE1 inhibition efficacy [215, 217, 218]. Numerous studies have evaluated AZD1775 in combination with DNA-damaging agents. AZD1775 reversed the cisplatin resistance of TNBC more significantly than ATR or CHEK1 inhibitors because AZD1775 induced premature mitotic entry more potently than ATR/CHEK1 inhibitors [219]. AZD1775 enhanced the sensitivity of TNBC to PARP inhibitors by diminishing the expression of the HRR proteins RAD51 and Mre11 [220]. The ATR inhibitor AZD6738 sensitized TNBC cells to the antitumor effect of AZD1775 by causing DNA damage and mitotic catastrophe. Moreover, the WEE1-like kinase PKMYT1 was also discovered. In cancer cells that overexpress cyclin E, the unexpected activation of CDK1 is triggered by using RP-6306 or PKMYT1 inhibitors alone or combined with gemcitabine have shown powerful in vivo antitumor activity in xenografts or PDX models [221].

To conclude, the WEE1 inhibitor AZD1775 monotherapy has potential in HRD TNBC, and the combination of AZD1775 with chemotherapy or drugs targeting DDR pathways was effective in the preclinical setting. WEE1 inhibitors are being increasingly studied in TNBC, but their potential response biomarkers are under research, and their clinical efficacy still needs to be demonstrated in clinical trials.

#### Emerging DDR target options and therapies

POLQ inhibitors could target HRD tumors. POLQ interacts with BRCA1, BRCA2, and ATM proteins and produces synthetic lethality [222]. POLQ repairs excised DNA ends via internal microhomology-mediated end joining in the alternative end-joining pathway [57]. POLQ contains a helicase-like ATPase domain that requires the replacement of RPA from excised ssDNA ends and the unravelling of homologous sequences. After alignment and annealing, the polymerase of POLQ fills the gap [223]. Moreover, POLQ inhibitors could exhibit radiosensitization in vitro in a range of different tumor cell lines, reflecting their critical role in DNA repair [224].

USP1 inhibitors could target *BRCA1/2* m tumors. Deubiquitylating enzyme USP1 is a negative regulator of transcription and synthesis by reversing proliferating cell nuclear antigen (PCNA) ubiquitination. PCNA during USP1 inactivation makes the replication fork unstable and USP1 is dependent on *BRCA1* [225]. Thus, in cancer cells, USP1 inactivation and *BRCA1* deletion are synthetically lethal. In TNBC cells, USP1 could enhances epithelial-mesenchymal transition to target tumor development [226].

Targeting WRN has potential to treat MSI tumors, and helicase WRN is a synthetic lethal target in MSI cells [227]. In the absence of WRN, the SLX4-MUS81 endonuclease complex cuts non-B-DNA secondary structures and causes chromosome fragmentation and apoptosis [228]. Therefore, inhibition of WRN helicase activity may be effective in MSI cancers.

In summary, there are various drug treatments targeting DNA repair pathways in breast cancer. Synthetic lethality and elevated replication stress aid in the effectiveness of DDR-targeted interventions by inhibiting molecules involved in DNA repair and blocking compensatory DNA repair pathways. Among these, PARP inhibitors for HRD-positive tumors are the most studied, with multiple clinical trials investigating monotherapy or combined regimens. Other anti-cancer strategies, such as RAD51 inhibitors, DNA-PKcs inhibitors, POLQ inhibitors, and berberine targeting the BER pathway [229] are still under preclinical investigation. Moreover, these DDR-related therapies also help to overcome the resistance of DNA-damaging chemotherapy and radiotherapy.

## DDR biomarkers and measurements

**Fig. 5:**
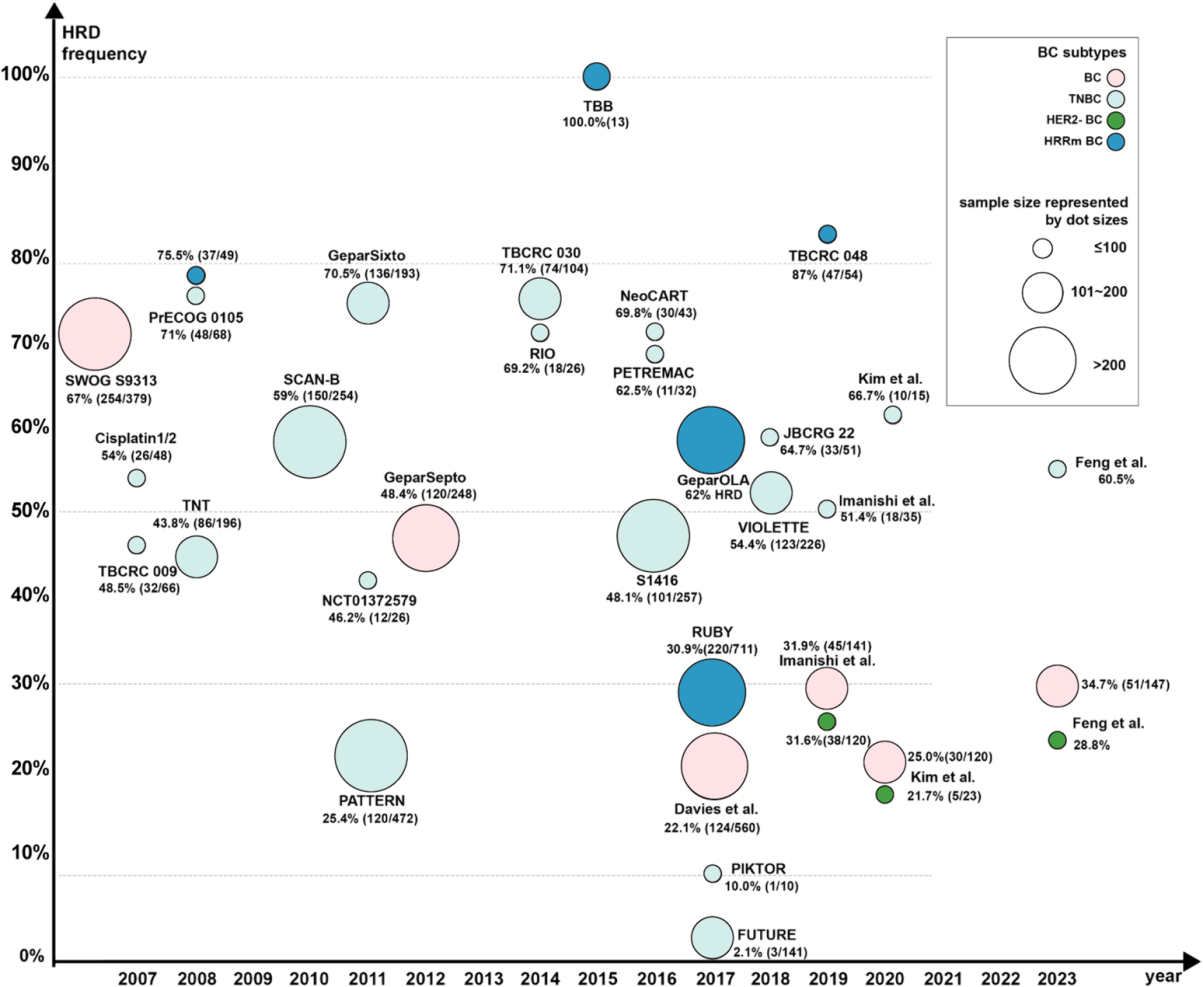
Summary of clinical trials with HRD stratification in breast cancer. Since 2007, the studies on HRD evaluation in breast cancer have been conducted systematically; more clinical trials with HRD stratification have been published in recent years. The x-axis represented the publication year of the study. The y-axis represented the percentage of breast cancer with HRD features in all breast cancer population. Each spot was a study with a size matched its population. Different colors showed different breast cancer population (pink, breast cancer (without subtype information); red, TNBC; orange, HER2-negative breast cancer; blue, breast cancer with HR-related genes mutations). HR-related genes refer to *BRCA1/2* and *BRCAness* gene (*RAD51, CDK12, PALB2, ATM, CHEK2*, etc.) mutations. The prevalence of HRD differs among different breast cancer subgroups. TNBC tends to demonstrate higher HRD proportion (all > 30%), thus its spots were concentrated on the upper part of the graph; vice versa for HER2-negative breast cancer in the lower part with lower HRD frequency. HRD, homogenous recombination deficiency; BC, breast cancer; TNBC, triple-negative breast cancer

Clinical trials of breast cancer with DDR features have proven their potential of benefiting from targeted anti-tumor therapies, while the combined therapy often involves more complicated toxicity issues. It is necessary to further characterize the tumor progression in PARP inhibitor therapy at the molecular level to obtain meaningful response molecular markers and screen patients who can benefit from various combination therapies. Most studies on DDR biomarkers in breast cancer concentrate on HRD measurements (summarized in Fig. 5 and Supplementary Table 6), as well as other less commonly used MMR-related evaluations and biomarkers in other DDR pathways.

### HRD biomarker and measurements

Since the discovery of the synthetic-lethal interaction between PARP inhibition and *BRCA1/2* deficiencies, multiple studies have demonstrated its clinical benefits in *BRCA1/2*-mutated tumors [230]. *BRCA1/2* mutation non-carriers with HRD features can also benefit from PARP inhibitors, [118, 121, 141] thus, a major challenge has been to identify HRD tumors beyond g*BRCA*m. HRD can be used as a biomarker for breast cancer stratification. Due to its stability, HRD will produce specific, quantifiable, and stable genomic changes, and HRD scores are highly consistent among different parts of the same tumor [231]. HRD-positive breast cancers share the characteristics of higher pathological grade, Ki-67 index and *TP53* mutation rate [36, 42, 43]. They also tend to be more ER or PR-negative and are often present with triple-negative breast cancer [42–44]. Meanwhile, HRD-positive breast cancer, and even in *BRCA* wild-type subgroups, can benefit significantly from treatment with PARP inhibitors and have different prognosis [118, 121].

The genomic changes produced by HRD serve as the theoretical basis for clinical detection methods. HRD can be evaluated by HR related genetic testing, genomic scar measurements, gene expression signatures of ‘*BRCAness*’, functional HR assays, and algorithm-assisted HRD evaluation. In clinically approved assays, HRD status is usually evaluated using a *BRCA1/2* pathogenic variant status and a genomic instability score (GIS) [232].

### HR-related genetic testing

HR-related genetic testing is the first developed clinical detection method for HRD. Among them, *BRCA1/2* mutations are the most definite HR-related genes, as well as the most commonly used biomarkers to select potential patients and predict the efficacy of PARP inhibitors [113, 119]. The FDA has approved olaparib monotherapy for patients with g*BRCA1/2* m in metastatic HER2-negative breast cancer [113] and talazoparib for patients with HER2-negative, g*BRCA1/2* m locally advanced or metastatic breast cancer [119]. HR pathway genes are widely distributed, thus in addition to *BRCA1/2*, *PALB2, BRIP1, RAD51B, NBN, MRE11, ATM, ATR, CHEK2,* and *WRN* are associated with HRD breast cancer [106]. Because individuals carrying these mutations are relatively rare and the scope of HR-related genes has not been clearly defined, further prospective studies are needed to confirm their reliability of HR measurements.

*BRCA1/2* germline and somatic mutations are equally potent in predicting the efficacy of PARP inhibitors [233]. The detection of g*BRCA1/2* m as PARP inhibitor indications is relatively simple and approved by international guidelines [234]. On this account, g*BRCA1/2* mutations remain mainstream to determine the status of HRD in breast cancer. Via non-invasive blood or saliva samples, g*BRCA1/2* m can be measured by Sanger sequencing, multiplex ligation-dependent probe amplification (MLPA), MPS (high-throughput allows for discovering variants) or point mutations in DNA sequencing panels [235]. However, the population of breast cancer patients eligible for genetic testing is relatively limited, mainly based on age at diagnosis and family history [236]. Second, solely detecting some germline pathogenic *BRCA1/2* mutations may ignore a variety of *BRCA1/2* variations due to incomplete penetrance, variant of uncertain significance, as well as the non-coding DNA regions [6]. Also, a substantial number of patients with *BRCA* mutations fail to respond to PARP inhibitors, while *BRCA* mutation non-carriers could still respond [113, 119]. *BRCA1* and *RAD51C* promoter methylation also causes HRD but often incompatible with *BRCA* mutations in tumor tissue. However, the methylation levels are conflicting in predicting the clinical efficacy of PARP inhibitors [237, 238]. Therefore, incorporating more HR-related genes, additional mutation patterns, and comprehensive panels could help to devoid false negative HRD results.

### Genomic scar measurements

Some HR-proficient (HRP) tumors still retain the genomic scars of their evolutionary HRD tumor development process, making measure genomic scars a better choice to detect instinct HRD [232]. When HRD exists, DSBs will rely on other repair mechanisms, such as NHEJ and micro-homologous end junctions. These mechanisms in HRD tumors have low fidelity, making it easy to cause large genomic aberrations and chromosomal instability referred to as genomic scars [239]. Commonly used biomarkers of genomic scars include loss of heterozygosity (LOH) (loss greater than 15 Mb and less than the entire chromosomal length), telomeric allelic imbalance (TAI) (an allelic unbalanced chromosome segment extending to one subtelomere but does not exceed the centromere and is larger than 11 Mb), and large-scale state transition (LST) (two adjacent regions at the break sites at least 10 Mb in length with the space between them less than 3 Mb) [240, 241]. Among the three genomic scar markers, LST has the best HRD evaluation performance in low coverage whole genome sequencing (WGS) and can significantly reduce testing costs. [242] The sensitivity and specificity of this HRD test in TNBC is high, and it also largely corresponds to inter-chromosomal translocations and *BRCA1* germline mutations [241, 243].

At present, there are two HRD detection products for genomic scars approved by FDA, Myriad myChoice® CDx and FoundationFocusTM CDx BRCA LOH. Both methods use the pathogenic mutation status of *BRCA1/2* commonly evaluated by next generation sequencing (NGS) [242] along with a GIS using SNPs to integrate HRD status [241, 244]. In Myriad myChoice® CDx, patients with *BRCA* mutations or HRD scores comprehensively calculating LOH, TAI, and LST over 42 are considered HRD-positive [176]. FoundationFocusTM CDx BRCA LOH considers HRD-positive as *BRCA* mutant carrier or *BRCA* mutant non-carrier but with high LOH score (≥ 16% LOH fragments in the entire genome) [231, 241, 244]. Genomic scar analysis has been used as the criterion for identifying HRD-positive individuals and enrolling them in some prospective breast cancer studies [121, 245, 246].

Genomic scar analyses are the most widely used method for HRD in breast cancer clinical trials. On the one hand, genomic scars have expanded the potential responsive populations. The phase 2 PrECOG 0105 study of neoadjuvant carboplatin, gemcitabine, and iniparib, 71% (48/68) TNBC had high HRD scores over the 32% of *BRCA1/2* mutations [247]. In the two cisplatin neoadjuvant therapy studies, 54% (26/48) of the patients with stage II-III TNBC had high HRD scores and 19% had *BRCA* mutations [247]. On the other hand, the HRD evaluation via genomic scar analyses helps to precisely stratify prognosis and treatment responses. In the two cisplatin neoadjuvant therapy studies, HRD was significantly associated with higher residual cancer burden and pCR (HRD vs. HRP, 42.0% vs. 31.0%) [247]. In another Japanese study, 141 patients with stage II-III breast cancer were treated with neoadjuvant paclitaxel followed by fluorouracil, epirubicin, and cyclophosphamide (P-FEC) using GIS score. 32% (54/141) breast cancers had an HRD status, and high HRD score was associated with insensitivity of P-FEC regimen for TNBC [42]. In the phase 2 GeparSixto trial on TNBC of additional benefit of carboplatin to anthracycline/taxane-based for neoadjuvant chemotherapy, HRD TNBC had higher pCR (63.5% vs. 33.9%) [140]. In the phase 2 GeparOLA trial on HER2-negative of additional benefit of for neoadjuvant combined therapy of paclitaxel and olaparib or carboplatin, up to 62% of patients with high HRD scores did not have *BRCA* mutations, and HRD TNBC also had higher pCR (olaparib arm, 56.0% vs. 52.0%; carboplatin arm, 59.3% vs. 20.0%) in comparison with HRP TNBC [141]. However, in the phase 2 trial TBCRC 030 of neoadjuvant cisplatin versus paclitaxel in stage I-III TNBC, there was no evidence of an interaction between HRD and pathologic response to preoperative chemotherapy [248]. In the prospective phase 3 TNT trial evaluating carboplatin and docetaxel in metastatic TNBC, TNBC with *BRCA1/2* m had higher ORRs (68.0% vs 33.3%) and improved PFS (6.8 vs 4.4 months); while HRD TNBC did not demonstrate statistically different ORR (38.2% vs 40.4%) and PFS compared to HRP TNBC. This unimproved response to platinum hindered the predictive role for Myriad HRD assay [249].

There are some shortcomings of the commonly used genomic scar analyses. Genomic scars only detect the irreversible and heritable end product of HRD, which is not so indicative of a tumor’s current tumor biology to guide treatment. The HRD threshold is also related to different tumor types and the HRD analysis was mainly developed from genetic data from the Caucasian population [36]. Whether it is suitable for Chinese needs to be proven in large sample size and prospective clinical trials. Also, the threshold score of commercial kits may be affected by co-mutations including *TP53* and *ATM* [250]. Therefore, more studies are needed to confirm the precise cutoff value and efficacy in breast cancer.

### Gene expression signatures of “*BRCAness*”

In the absence of *BRCA* mutations, HRD status may still provide hints about the benefits of platinum-based drugs and PARP inhibitor therapy. Therefore, detection methods that incorporate more genes and variation modes of HR-related genes (or *BRCAness* genes) are also being developed. Gene expression signature analyses based on mutation lineage can fully reflect the endogenous and exogenous mutations and HR repair processes of tumor cells. Among them, HRDetect relies on genome-wide WGS and predicts *BRCA* deficiency by additionally recognizing *BRCA* transcriptions and promoter hypermethylation. This signature achieves a sensitivity up to 86% in breast cancer superior to that of GIS [5, 176]. The RIO trial using HRDetect in circulating DNA found that most TNBC present HRD and can benefit from neoadjuvant rucaparib [38]. But HRDetect has not been applied in predicting therapy response.

Other DDR deficiency assays include the BRCAness 77-gene signature in the I-SPY 2 trial comparing the efficacy of carboplatin with PARP inhibitors versus standard chemotherapy [251], a 44-gene panel based on WGS which successfully predicted the response to preoperative and postoperative chemotherapy, as well as to explore the association between HRD and immune microenvironment with increased TILs [252], a 228-gene multi-scale exome-sequenced signature [253], which further proves that mutational and phenotypic HRD profiles persist regardless of HR-related gene defects. Single base substitution Signature 3 (SBS Signature3) has been proven to be highly correlated with *BRCA* mutations and *BRCA1* promoter methylation in breast cancer and other tumors [254]. The phase 2 TBCRC 048 trial used the BRCAness signature and proved that patients with germline *PALB2* mutations and somatic *BRCA* mutations beyond g*BRCA1/2* m are also responsive to olaparib [245]. In the phase 2 PETREMAC study, primary TNBC received neoadjuvant olaparib, and HRD was evaluated by targeted DNA sequencing (360-gene panel) [255]. HRD could predict response to olaparib, and most respondents had no *gBRCA1/2* mutations. Low RAD51 scores, high TILs, or high PD-L1 expression were associated with responses to olaparib [255].

However, these gene expression signatures analyses based on mutation lineage characteristics have restricted clinical use due to limited diagnostic accuracy, specificity, the absence of exact therapy- or assay- dependent cut-off values [5, 256]. And sampling from FFPE of the primary tumor makes it fail to reflect the evolution of tumors and the intrinsic accumulation of genomic scars.

### Functional HR assays

Functional detection of HRD is usually performed by evaluating mRNA expression of HR functional biomarkers. Among them, the absence of RAD51 foci revealing HRD is the most commonly used in functional HR assays [208]. Functional HR assays in FFPE tumor samples from g*BRCA1/2* m patients treated with PARP inhibitors predicted clinical efficacy and drug resistance [208]. In a broader context of DNA damage, novel genotoxicity assays including functional γH2AX foci assay and comet assay are particularly sensitive to DNA damage induced by radiation or genotoxicants [257].

In summary, the formation of RAD51 foci shows promise as a potential method for identifying patients who could benefit from treatment with PARP inhibitors. Genetic and functional assays are likely to become routine for selection of patients for DNA damage response inhibitor therapy in the clinic. However, while functional assays are currently only available for PARP inhibitors, their practical implementation in clinics is still in progress. Challenges such as limitations in FFPE sample selection, underdeveloped analysis systems, and lack of specificity also need to be addressed in order to fully optimize the use of these assays. Future efforts should focus on the development of functional assays predicting the efficacy of other DNA damage response-targeting therapies.

### Algorithms-assisted HRD evaluation

Insufficient performance on present HRD measurements may result from limited inclusion of HRD-related and clinicopathological features. The artificial intelligence (AI) assisted algorithms can improve computing power and make full use of genomic variations and clinicopathological information. This establishment of comprehensive algorithms-assisted HRD evaluation can effectively improve accuracy and generalization of traditional HRD score calculations to classify breast cancer with an AUC up to 0.98 [5, 240]. A new genome-wide mutational scar-based test, CHORD (Classifier of Homologous Recombination Deficiency) has also been developed to predict HRD without data published regarding its predictive value of response to PARP inhibitors [38]. The advantage of AI-assisted algorithms over traditional sequencing or protein measurements is to better represent the HRD status and genomic instability of the genome in a comprehensive manner. However, WGS-based algorithms require the acquisition and analyses of large and complex data.

In summary, HRD helps to predict the efficacy of chemotherapy (platinum drugs that cause cross-linking between DNA strands) and PARP inhibitors in breast cancer patients. Most importantly, HRD testing expands the potential populations beyond breast cancer with *BRCA1/2* mutation. *BRCA* genetic testing is first widely approved as a HRD diagnostic tool, and complex HRD measurements, including commonly used genomic scar assays are under investigation to determine the cut-off values or to personalize the DDR-related panels. However, techniques for determining HR status in patients often are currently underutilized and yield results that are simplified into binary HR deficient or proficient classifications in clinical practice. The complexity of HRR components results in no unified standard for which biomarkers to include, and there is no consensus measurement method for breast cancer. Third, most studies with HRD markers beyond g*BRCA*m have relatively small sample studies, and the practice evidence is not enough. More effective measurement methods, including NGS and AI-aided algorithms, could help incorporate versatile HR-related characteristics of breast cancer and achieve higher sensitivity and specificity.

### MMR/MSI biomarker and measurements

Routine measurements of MMR status in breast cancer are not typically performed (studies including MMR/MSI features are in Supplementary Table 7), unless there is evidence of LS-related cancer [258]. There are various methods available for measuring MMR in breast cancer, including polymerase chain reaction (PCR)-based genetic testing, immunohistochemical (IHC) staining of loss of MMR protein expression, and NGS methodology and algorithms with expanded panels. The choice of approach can be influenced by factors such as cost, accuracy, and ease of implementation.

### IHC

One classic method for measuring MMR protein expression is IHC staining, the first-line method based on the functionality of MMR in LS-related cancers [259, 260]. IHC shows loss of MMR proteins including MLH1, MSH2, MSH6, and PMS2. Deficiency of MMR means absent nuclear staining of IHC expression of at least one MMR protein, which provides an easy and economical approach. However, IHC antibodies may not always yield satisfactory results in breast cancer, and its application is sometimes limited by miscellaneous workflows. Furthermore, while normal IHC can identify cases with intact MMR, it may miss cases with deficient MMR beyond the four proteins typically tested, and PMS2 analysis may be infeasible due to multiple highly homologous pseudogenes [259].

### PCR

PCR is considered the golden standard for evaluating microsatellite (MSI) or MMR in breast cancer, with the NCI PCR pan-cancer panel being commonly used [261]. The NCI PCR pan-cancer panel includes two mononucleotides (BAT25 and BAT26) and three dinucleotide loci (D2S123, D5S346, D17S250). Alternative panels with more mononucleotide repeats obtain higher specificity and sensitivity (pentaplex BAT-25, BAT-26, NR-21, NR-24, NR-27; Promega, BAT-25, BAT-26, NR-21, NR-24, MONO-27) [262]. The MSI-high phenotype is characterized by a shift (usually downward) in at least two of the five microsatellite loci. Higher accuracy can be achieved by detecting more, like dozens or even hundreds of loci in breast cancer, including *TP53* and *D11D988* [262, 263]. MMR protein loss is more frequently detected than microsatellite instability in breast cancer [71, 263] and genomic signature profiling may outperform the prevailing protein-based methods for dMMR arising later in disease development or present only at subclonal levels [264]. Patients are first classified by MMR protein status via IHC with both tests available [265]. The inconsistency between MMR protein profiles and MSI genetic sequencing in some cases necessities the combined measurements to avoid false negatives and possibly maximize health economic cost-effectiveness [260].

#### NGS and other algorithms

High-throughput NGS-based panels that simultaneously investigate multiple genes have gained popularity in recent years and can provide information on TMB and other mutations with therapeutic significance such as *BRCA1/2* and *ESR1* [266]. It also shares high concordance with PCR and IHC [267]. However, their wide application is limited by high cost and technical complexity [234]. Targeted-specific panels avoid the costs of whole genome sequencing or whole exome sequencing and focus on the most clinically useful genes compared to large panels with higher rates of variants of undetermined significance [268, 269]. Moreover, NGS panels that are previously designed for LS may be biased in breast cancer and cause false negatives [268]. Breast cancer requires at least 50 genetic markers (much more than in other cancers) to achieve over 90% sensitivity and specificity [269], among which the most informative markers do not overlap with those in standard MSI clinical assays [63]. This suggests that diagnosing MSI for breast cancer demands systematic and genome-scale identification [269]. Furthermore, algorithms based on NGS techniques have emerged recently with accurate MSI calculation at numerous microsatellite loci, including MSIsensor [270], PreMSIm [271], and FoundationOne CDx [272] and so on. These platforms generally hold high accuracy of over 95% and high concordance with IHC and PCR [273, 274].

In summary, the optimal measuring approaches for determining MMR status in breast cancer remain a subject of debate, with various methods available that offer different levels of accuracy, ease of implementation, and cost-effectiveness. A combined approach using multiple methods may be necessary to avoid false negatives and maximize health economic cost-effectiveness. Emerging algorithms based on NGS techniques are showing promise, but further research is needed to optimize the accuracy of MSI detection in breast cancer.

### Other DDR-related biomarker and measurements

In spite of biomarkers of HR and MMR, other DDR mechanisms have no exclusive biomarkers and specific assessments on their activity yet. Key proteins, such as PARP, RAD51, and P53, are shared among different DDR mechanisms, and to some extent can be the biomarkers and indirectly evaluate other DDR mechanisms. Deeper and broader understanding on the complicated mechanisms of DDR could help to demonstrate new effective DNA repair components as potential DDR biomarkers, especially those targeting BER, NER, and NHEJ, which appear frequently in breast cancer.

### Challenges and perspectives

Response to DNA damage is a complex process involving various signaling networks and proteins that are differentially activated or deactivated in specific cancer types. On the one hand, genomic instability caused by defective DNA damage response could result in dysregulated cell cycle and cancer development. On the other hand, increased replication stress and DNA repair defects in tumors provide an opportunity for us to treat cancer, making cancer cells more susceptible to DDR inhibition than normal cells. Breast cancer is a complex and heterogeneous disease that involves multiple DNA repair pathways. This review investigates the significance of DNA repair pathways in breast cancer, with a specific focus on various pathways including BER, NER, HR, NHEJ, MMR, ICR or Fanconi anemia pathway. The study also examines the role of these pathways in breast cancer drug resistance and explores their potential as targets for cancer treatment, especially PARP inhibitors in HRD breast cancer.

The identification of DDR has created a promising opportunity for tailored treatment strategies in breast cancer patients through the utilization of DDR-targeted therapies and combination therapies. Furthermore, individualized treatment approaches focusing on specific DNA repair pathways tailored to the subtype or genetic makeup of the breast cancer are currently under development. The detection of germline or somatic mutations, particularly those related to *BRCA1/2* or *BRCAness*, plays a significant role in patient stratification. Other companion diagnostic tests that measure complex ‘genomic scars’ are also being used to select eligible patients and comprehensively evaluate the HRD status. As an alternative to genetic tests, functional assays measuring the formation of RAD51 foci are also capable for evaluating HR activity and predicting predict PARP inhibitor response. Advances in genomics and imaging technologies can also potentially improve patient stratification and identify biomarkers of treatment response. However, many challenges remain, including drug resistance, toxicity, and the need for more personalized treatments.

#### Strategies for PARP inhibitor resistance

**Fig. 6:**
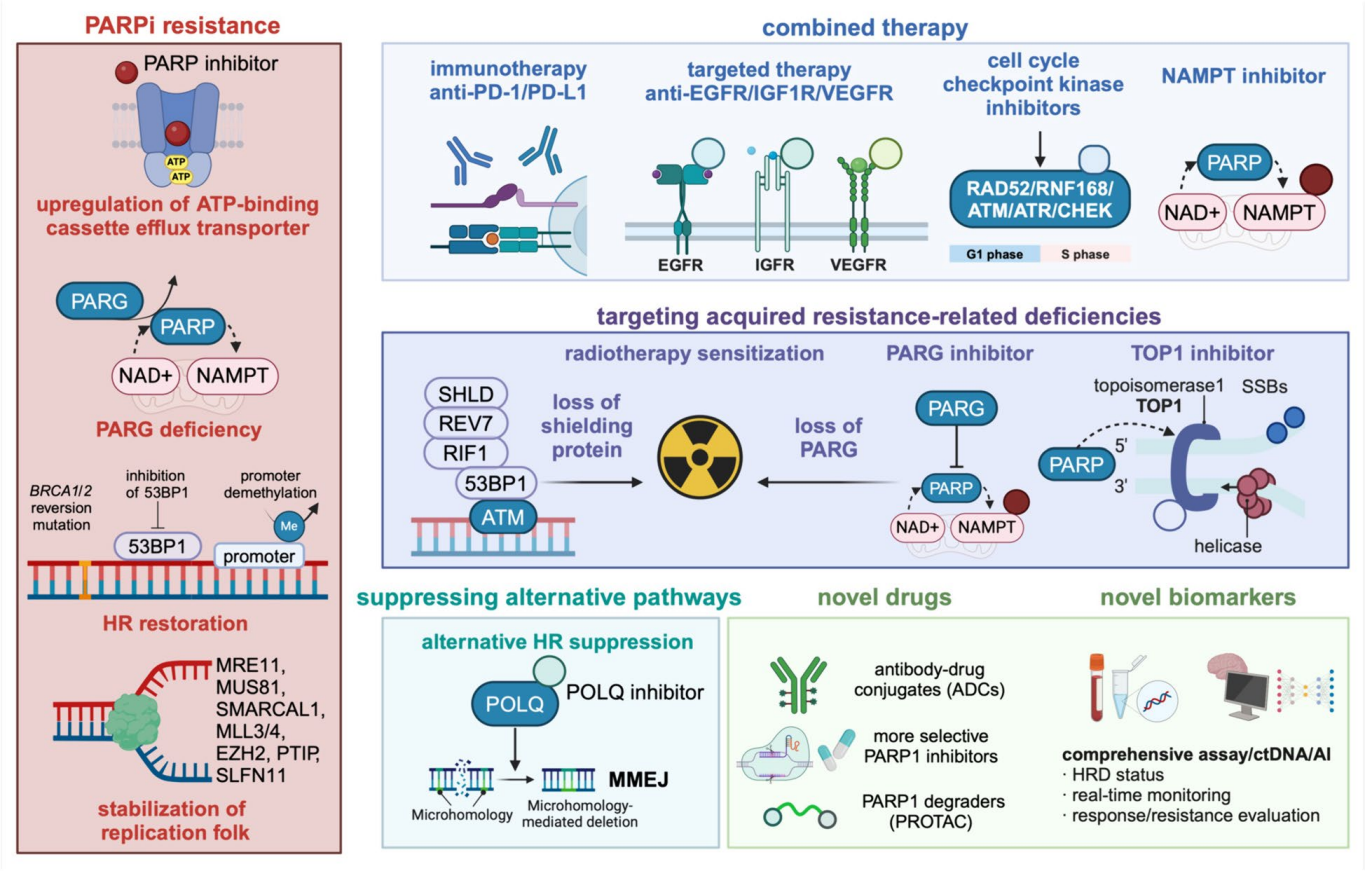
Mechanism of PARP inhibitor resistance and strategies to overcome. The efficacy of PARP inhibitors in patients with breast cancer is limited by the development of mechanisms of resistance in most patients. Many mechanisms of resistance have been characterized, including upregulation of drug efflux transporters, restoration of homologous recombination activity, and mitigation of the replication stress via replication fork stabilization. Novel strategies are under investigation to overcome resistance to PARP inhibitors, such as new drug combinations and targeting acquired deficiency. Ongoing research efforts are also uncovering novel biomarkers to identify additional patients who may benefit from these agents. HR, homologous recombination; ctDNA indicates circulating tumor DNA; PD-1, programmed cell death-1 protein; PD-L1, programmed cell death-1 ligand protein; PARP, poly (ADP-ribose) polymerase; PARG, poly (ADP-ribose) glycohydrolase; PROTAC, PROteolysis-TArgeting chimera

Despite the well-known benefits of PARP inhibitors for patients with *BRCA1/2*-associated breast cancer, resistance often occurs. Mechanisms of resistance to PARP inhibitors have been identified, but how to effectively target them remain largely unexplored [275]. Three primary strategies proposed to overcome resistance are combining with synergistic anti-tumor drugs, novel therapeutics to targeting acquired resistance-related deficiencies and suppressing mutant phenotypes (mechanisms of PARP inhibition resistance and strategies to overcome are summarized in Fig. 6).

### Combined therapy

Combination therapies include inhibiting alternative HR pathways, indirectly inhibiting HR, inhibiting cell cycle checkpoints, and immunotherapy. Suppressing the alternative HR path can be achieved by suppressing RNF168 or RAD52. BRCA1 can recruit PALB2-BRCA2 complex to ssDNA and complete HR. However, in *BRCA1*-deficient cells, PALB2 is recruited into ssDNA in an RNF168-dependent manner [276]. Therefore, inhibiting the function of RNF168 may be effective in *BRCA1*m tumors and can reverse PARP inhibitor resistance to some extent. For RAD52, the co-depletion of RAD52 and BRCA1/2 can produce synthetic lethality, suggesting its role an alternate pathway that enables RAD51 to obtain excised DNA ends in the absence of BRCA1/2 [277]. RAD52 may also play a role in replication fork reversal stagnation [278]. Therefore, RAD52 inhibitors may serve as one of the alternative approaches for targeted treatment of *BRCA1/2*-deficient tumors. Indirect HR inhibition can be achieved by targeting epigenetic regulators, for example, inhibition of histone deacetylase can lead to HR downregulation, thereby increasing the sensitivity of PARP inhibitors [279]. Moreover, targeting EGFR, IGF1R, VEGFR or PI3K-AKT pathways also damage HR [280]. Other potential combined therapies include NAMPT inhibitor that inhibits tumor growth by regulating ATP levels and NAD + metabolism [124].

The combination of immune checkpoint inhibitors and PARP inhibitors has a synergistic effect. HR-deficient tumors have high mutation loads, and lead to an increase in tumor-specific neoantigens and trigger a series of signal transduction, thus activating the immune response. In clinical studies, the combination of PARP inhibitors with anti-PD-1 antibodies is generally well tolerated and increases the rate of objective response compared to monotherapy [123, 154, 155]. Currently, a variety of cell cycle checkpoint kinase inhibitors are also under research and their anti-tumor effects in combination with PARP inhibitors are evaluated, including inhibition of CHEK1/2, ATM, ATR, and WEE1 in breast cancer [169, 180, 220].

### Targeting acquired resistance-related deficiencies

Tumor resistance to chemotherapy often accompanies acquired resistance defects, which can theoretically be targeted to improve subsequent treatment efficacy [124]. For example, loss of PARG and 53BP1-RIF1-REV7 shielding proteins can lead to resistance to PARP inhibitors but can increase sensitivity to radiation therapy [281]. PARP1 has repair functions for the topoisomerase 1 (TOP1) cutting site, so PARP inhibitor-resistant tumors are more sensitive to TOP1 inhibitors in subsequent use with improved treatment efficacy [282]. Given the synergistic effect of TOP1 and PARP inhibitors and their potential for synthetic lethality, the results of combining PARP inhibitors with TOP1 inhibitor-loaded antibody–drug conjugate (ADCs) (such as Sacituzumab or T-Dxd) are promising [275]. In addition, downregulation of PARG can lead to metabolic exhaustion of NAD + and increased capture of chromatin by PARP1, so these cells are sensitive to alkylating agents temozolomide as a potential strategy for responding to PARP inhibitor resistance [283].

### Suppressing alternative HR pathways

Directly suppressing the alternative HR pathways could also address PARP inhibitors resistance. Repair of DSBs in the absence of HR is achieved by upregulation of MMEJ, an error-prone repair pathway driven by the low-fidelity DNA polymerase θ POLQ [104]. Therefore, inhibiting POLQ could be effective in PARP inhibitor-resistant tumors benefited by the synthetic lethality between POLQ deletion and HR [138]. However, whether POLQ inhibitors should be used alone or in combination with PARP inhibitors, in all PARP inhibitor-resistant tumors or only for resistant tumors caused by specific mechanisms and can be applied to HRD tumors other than genomically unstable *BRCA1/2* m tumors, are needed to clarify before clinical use.

### Enhance selectivity to mitigate off-target toxicities

The first obstacle in DDR-related therapies is the drug toxicity and resistance. The mechanism of secondary resistance to PARP inhibitors has not been fully elucidated. In addition, PARP inhibitors have cross-resistance with platinum drugs, and patients who progress in front-line platinum therapy often do not respond well to PARP inhibitors [284]. Therefore, strategies to overcome drug resistance should be actively explored, especially PARP inhibitors combined with other DDR targeted drugs, immunotherapy, chemotherapy and other means should be further explored. In preclinical studies, cell line panels, PDXs, and organoids, could help to identify potential biomarkers for new DDR-targeted therapies [7, 285]. Prospective studies with large DDR deficient populations are needed to guide the use of DDR status in precise biomarker-targeted therapy. Also, identifying other molecular defects that induce synergistic effects is a sensible approach to defining predictive biomarkers.

Targeting DNA repair pathways can have significant side effects, including an increased risk of developing secondary cancers. Thus, it is important to enhance drug selectivity to mitigate off-target toxicities. On the one hand, substantial progress has been made towards tackling this challenge with the identification of specific genetic alterations that make tumors vulnerable to DDR-targeting treatments with selective toxicity, especially for *BRCA1/2* mutation and PARP inhibitors [31]. On the other hand, most regulators in DDR pathway are scaffold proteins important for signal transduction, but without any enzyme activity and complicates the design of small-molecule inhibitors for targeting [15]. In the field of drug design, the alternative solution could be the PROteolysis-TArgeting Chimeras (PROTAC). It selectively degrades proteins of interest through the cellular ubiquitination system [286]. CRISPR/Cas9-mediated gene editing could also be applied to inactivate key scaffold proteins selectively [287]. Future research should focus on developing more precisely-designed targeted strategies to mitigate these safety concerns of DNA repair inhibitors.

Furthermore, the unavoidable on-target toxicity necessitates the need to identify predictive biomarkers for both broadening current therapeutic indications and consecutively monitoring the response. A more efficient strategy could be included altered immune TME features as potential biomarkers, such as HRD DNA Damage Response Deficiency characterized by up-regulated immune process [288]. To fully realize the potential of HRD as a biomarker for breast cancer, new technologies for evaluating HRD in breast cancer are needed. Thus, NGS and algorithms in a more comprehensive panel offer promising options with selected sensitive loci beyond the *BRCAness* genes and a tailored cut-off value. In addition to these biopsy-dependent detections, non-invasive HRD liquid biopsy from cell-free DNA and circulating immune and tumor cells, can achieve comparable performance and enable continuous in breast cancer. While large-scale screening for HRD using genomic markers is economically challenging, AI provides a robust deep-learning method with routinely stained tissue pathological slides and helps to identify HRD-related morphological phenotypes accurately [289, 290]. Not only do deep-learning models help to stratify HRD status, but also potentially predict prognosis and guide therapeutic decisions [291, 292].

### Combination with more cancer hallmarks and therapies

Another consequence of the genomic instability in DDR-mutated tumors is the rewiring of their genetic make-up to escape drug toxicity and develop acquired resistance to therapy. The design of drug combinations, with optimized timing and dosing regimens, that reduce toxicity and limit or suppress resistance could tackle these problems [138]. Studies on the interaction of DDR-related therapies and other treatments (chemotherapy, radiotherapy, immunotherapy, new targeted and combined therapies) have facilitated the synergistic therapeutic effects in drug-resistant breast cancer patients. More prospective studies with large samples in the existing combined therapeutic regimens, and in the newly developed combined treatments await to be conducted. For example, CDKs block DNA end resection, mediate phosphorylation of the MRN complex and CtIP, and contribute to PARP inhibition resistance [293]. Its inhibitors might overcome the PARP inhibitor resistance. There has been a multi-center phase 1/2 clinical trial (NCT05252390) on the combined CDK4/6 inhibitor (NUV-868) with olaparib in advanced breast cancer which aims to reveal the superior therapeutic effect than PARP inhibitors alone.

A better understanding of the combination of DDR-related mutations and metabolisms could lead to the design of more precise drugs to combat cancer resistance. In addition to the current DDR-related therapies targeting a limited number of pathways, future research should aim to identify more potential molecules in more DNA repair pathways and understand their role in breast cancer pathogenesis. This deep exploration on DDR mechanisms through CRISPR/Cas9 gene editing and single-cell sequencing, as well as comprehensive genetic sequencing to select the targeted therapy, are hopefully to provide novel and more precise anti-tumor strategies. Different novel strategies could take advantages of synthetic lethality, TME or metabolism regulation. And future research should focus on identifying predictive biomarkers.

## Conclusions

In conclusion, DNA damage response plays a crucial role in the pathogenesis, advancement, and treatment responses of breast cancer. PARP inhibitors have shown promising results in treating breast tumors with HRD status and beyond, and are progressing towards clinical implementation. While targeting these pathways has demonstrated the potential in breast cancer management, drug toxicity and resistance must be overcome. Moreover, future research efforts will be directed towards identifying the optimal combination of DDR inhibitors, determining the most effective regimen to minimize adverse reactions. Additionally, investigating DDR mechanisms dependent on effective DDR biomarkers in each tumor to identify specific dysfunctions will help personalize treatment. Improving patient stratification by tumor subtypes or genetic profiles is crucial for developing tailored treatment strategies.

## Supplementary Information

Supplementary Material 1.

## Data Availability

No datasets were generated or analysed during the current study.

## Abbreviations

a-NHEJ: alternative nonhomologous end joining
AP: apyrimidine
ATM: ataxia–telangiectasia mutated
ATR: ataxia-telangiectasia mutated rad-3-related
ATRIP: ATR-interacting protein
BC: breast cancer
BER: base excision repair
CDKs: cyclin-dependent kinases
cfDNA: cell-free DNA
cGAS: cyclic GMPeAMP synthase
CK: casein kinase
CKIs: CDK inhibitors
CIP2A: cellular inhibitor of PP2A
CtIP: CtBP-interacting protein
CR: complete response
CRC: colorectal cancer
DDR: DNA damage response
dMMR: deficient DNA mismatch repair
DNA-PKcs: DNA-dependent protein kinases
dNTPs: Deoxyribonucleoside triphosphates
dRP: deoxyribose phosphate
DSBs: DNA double-strand breaks
EXO1: exonuclease 1
FFPE: formalin-fixed paraffin-embedded
HR: homologous recombination
GG-NER: global genome NER
HRD: homologous recombination deficiency
ICB: immune checkpoint blockade
IFN: interferon
IHC: immunohistochemistry
IRF3: interferon regulatory factor 3
LSTs: large-scale transitions
MDC1: mediator of DNA damage checkpoint protein 1
MMR: mismatch repair
MRE11: meiotic recombination 11 homolog 1
MRN: MRE11-RAD50-NBS1
MSI: microsatellite instability
MSI-H: high microsatellite instability
NER: nucleotide excision repair
NGS: next-generation sequencing
NK: natural kill
NK-κB: transcription factor nuclear factor κB
OS: overall survival
PD-1: programmed cell death-1 protein
PD-L1: programmed cell death-1 ligand protein
PARP: poly (ADP-ribose) polymerase
PARG: poly (ADP-ribose) glycohydrolase
PCR: polymerase chain reaction
PFS: progression-free survival
PR: partial response
RCT: randomized clinical trials
ROS: reactive oxygen species
RNR: ribonucleotide reductase
RPA: replication protein A
SSB: single strand breaks
ssDNA: single-strand DNA
STING: stimulator of interferon genes
TAI: telomeric allelic imbalance
TC-NER: transcription-coupled NER
TGF-β: transforming growth factor (TGF)-β
TILs: tumor infiltrating lymphocytes
TMB: tumor mutation burden
TNBC: triple-negative breast cancer
TOP1: topoisomerase protein 1
TOPBP1: topoisomerase 2-binding protein 1
Treg: regulatory T cells
UV: ultraviolet
WGS: whole-genome sequencing
WES: whole-exome sequencing
XRCC1: X-ray repair cross-complementing protein 1
