# Supplementary material for: DNA damage response in breast cancer and its significant role in guiding novel precise therapies
Source: Biomark Res. 2024 Sep 27;12:111. doi: 10.1186/s40364-024-00653-2 (PMC11437670; doi:10.1186/s40364-024-00653-2)
Supplement: Supplementary file 1 — Supplementary Material 1. [file 40364_2024_653_MOESM1_ESM.pdf]

Additional files: DNA damage response in breast cancer and its significant role in guiding novel precise therapies

Jiayi Li[1,2†], Ziqi Jia[1†], Lin Dong[3†], Heng Cao[1], Yansong Huang[1,2], Hengyi Xu[2,4], Zhixuan Xie[2], Yiwen Jiang[2], Xiang Wang[1*], Jiaqi Liu[1,4*]

Supplementary Table 1. Clinical trials of monotherapies targeting DNA damage repair pathways in breast cancer 2

Supplementary Table 2. Clinical trials of therapies targeting DNA damage repair pathways combined with (neo)adjuvant chemotherapy in breast cancer........ 6

Supplementary Table 3. Clinical trials of therapies targeting DNA damage repair pathways combined with immunotherapy in breast cancer 9

Supplementary Table 4. Clinical trials of therapies targeting DNA damage repair pathways (PARPi and ATRi) combined with radiotherapies in breast cancer 12

Supplementary Table 5. Clinical trials of therapies targeting DNA damage repair pathways (PARPi) combined with targeted therapy in breast cancer 13

Supplementary Table 6. Clinical trials in breast cancer measuring or stratified by HRD assessment 15

Supplementary Table 7. Studies on MSI-H/dMMR or related to mismatch repair in BC in recent 20 years........ 30

Supplementary Table 1. Clinical trials of monotherapies targeting DNA damage repair pathways in breast cancer

Target	Initiation year	Country	Phase	Clinical trials	Name	Conditions	Drugs	BC sample (n)
PAPR	2010	USA & Spain	I	NCT04053673 (recruiting)		HR deficient BC	RBN-2397 (PARP7 inhibitor)	130 (total)
	2010	multi	I	NCT01078662 (active, not recruiting)		g*BRCA1/*2m BC	olaparib	298
	2011	USA & UK	I	NCT01286987 (completed) [1]		advanced g*BRCA1/2*m BC	talazoparib	12
	2019	USA	I	NCT03955640 (recruiting)		BC with chest wall recurrences	olaparib + hyperthermia treatment	3 (estimated)
	2019	USA	I	NCT04041128 (recruiting)		BC	olaparib	14
	2019	USA	I	NCT03911453 (active, not recruiting)		stage I-III TNBC	rucaparib	20
	2021	Czechia & Poland	I	NCT05002868 (active, not recruiting)		locally advanced or metastatic BC	RP12146	23
	2023	North America	I	NCT05564377 (recruiting)	ComboMATCH	locally advanced or metastatic BC through ComboMATCH screening	olaparib	2900 (total)
	2012	UK	I/II	NCT04644068 (recruiting)		TNBC	E7449 (+ temozolomide / carboplatin and paclitaxel)	41
	2014	USA	I/II	NCT01989546 (completed) [2]		advanced *BRCA*m BC	talazoparib	9
	2017	Japan	I/II	NCT03343054 (active, not		pretreated advanced or metastatic g*BRCA1/2*m	talazoparib	19

Target	Initiation year	Country	Phase	Clinical trials	Name	Conditions	Drugs	BC sample (n)
				recruiting)		BC		
	2017	China	I/II	NCT03805399 (umbrella, recruiting) [3]	FUTURE	refractory TNBC (basal-like immune-suppressed)	PARP inhibitor	4
	2020	USA	I/II	NCT04503265 (active, not recruiting)	ATLAS-101	advanced or metastatic BC	AMXI－5001	122 (estimated)
	2020	USA & Australia	I/II	NCT04672460 (completed)		g*BRCA1/2*m BC or somatic *BRCA1/2* mutations	talazoparib	73 (total)
	2007	UK	II	NCT00494234 (completed) [4]	ICEBERG 1	advanced *BRCA*m BC	olaparib	54
	2008	Canada	II	NCT00679783 (completed) [5]		TNBC	olaparib	26
	2014	UK	II	EudraCT 2014-003319-12 (completed) [6]	RIO	TNBC	rucaparib	43
	2015	USA	II	NCT02401347 (completed) [7]	TBB	pretreated advanced HER2- BC with HRR-related gene mutations except *BRCA1/2*	talazoparib	13
	2016	Norway	II	NCT02624973 (active, not recruiting) [8]	PETREMAC	TNBC	olaparib	32
	2016	France	II	NCT02505048 (completed) [9]	RUBY	*BRCA*wt TNBC, pretreated advanced	rucaparib	41

Target	Initiation year	Country	Phase	Clinical trials	Name	Conditions	Drugs	BC sample (n)
	2018	USA	II	NCT03344965 (active, not recruiting) [10]	TBCRC 048	g*BRCA*wt HER2- BC, HRD BC with *BRCA*ness profile or *BRCA* somatic mutation metastatic BC with (s)*BRCA1/2* mutations or g/s mutations in HR-related genes other than *BRCA1/2*	olaparib	54
	2018	USA	II	NCT03367689 (terminated)	NOBROLA	*BRCA*wt HRD metastatic breast cancer	olaparib	7
	2018	Denmark	II	NCT03562832 (active, not recruiting)		metastatic BC selected by drug response prediction	2X-121	30 (estimated)
	2019	Belgium	II	NCT03967938 (recruiting)	1-2018 BSMO	advanced tumors with HRR gene mutations except *BRCA1/2*	olaparib	540 (estimated)
	2021	USA	II	NCT03990896 (recruiting)		somatic *BRCA* mutant metastatic BC	talazoparib	30 (estimated)
	2020	USA	II	NCT04171700 (terminated)	LOBESTAR	BC with deleterious mutations in HRR genes except *BRCA1/2*	rucaparib	83 (total)
	2021	USA	II	NCT04550494 (recruiting)		stage III-IV BC with DDR-related gene mutations	talazoparib	36 (estimated)
	2022	France	II	NCT05232006 (not yet recruiting)		advanced metastatic BC with germline	niraparib	12 (estimated)

Target	Initiation year	Country	Phase	Clinical trials	Name	Conditions	Drugs	BC sample (n)
						PALB2 mutations		
	2013	multi	III	NCT01945775 (completed) [11]	EMBRACA	advanced and/or metastatic *BRCA*m BC	talazoparib	431
	2014	USA & Europe	III	NCT01905592 (terminated)	BRAVO	HER2- g*BRCA1/2*m BC	niraparib	216
	2014	multi	III	NCT02000622 (active, not recruiting) [12]	OlympiAD	metastatic g*BRCA1/2*m HER2- BC	olaparib	302
	2018	multi	III	NCT03286842 (completed) [13]	LUCY	germline or somatic *BRCA*m HER2- BC	olaparib	253; 3
	2017	USA	III	NCT03329001 (active, not recruiting)		locally advanced or metastatic BC	niraparib	83 (total)
ATR	2012	USA & UK	I	NCT02157792 (completed) [14]		advanced BC	VX-970/M6620	1
	2023	USA	I/II	NCT05898399 (recruiting)		advanced or metastatic HER2- *BRCA*m BC	ART6043	250 (estimated)
PARG	2023	USA	I	NCT05787587 (recruiting)	IDEAYA	advanced g*BRCA1/2*m BC	IDE-161	68 (estimated)

BC, breast cancer; TNBC, triple negative breast cancer; HER2, Human Epidermal Growth Factor Receptor 2; BRCAm, *BRCA1/2* mutationgBRCA1/2m, germline *BRCA1/2* mutation; g*BRCA*wt, germline *BRCA1/2* wildtype; PARP, poly ADP-ribose polymerase; po, orally; qd, once daily; bid, twice daily; iv, intravenously.

Supplementary Table 2. Clinical trials of therapies targeting DNA damage repair pathways combined with (neo)adjuvant chemotherapy in breast cancer

Target	Initiation Year	Country	Phase	Clinical trials	Name	Conditions	Drugs	BC sample (n)
PARP	2007	USA	I	NCT00526617 (completed)		advanced BC	veliparib + temozolomide	41 (total)
	2008	USA	I	NCT01445418 (completed)		*BRCA1/2*m BC; sporadic TNBC	AZD2281 + carboplatin	103
	2010	USA	I	NCT01145430 (completed)		ER-/HER2-/PR-/male recurrent BC; stage IV BC; TNBC	veliparib + pegylated liposomal doxorubicin	45 (total)
	2011	USA	I	NCT01237067 (completed)		recurrent or refractory BC	olaparib + carboplatin	77 (total)
	2013	USA & Europe	I	NCT02033551 (completed)		advanced BC	veliparib (+ carboplatin/paclitaxel + fluorouracil, leucovorin and irinotecan)	47 (total)
	2017	Europe	I	NCT00516724 (active, not recruiting) [15]		BC	olaparib + carboplatin/paclitaxel	12
	2018	USA	I	NCT03641755 (active, not recruiting)		*BRCA1/2m* BC	olaparib + sapacitabine	10
	2012	UK	I/II	NCT04644068 (recruiting)		TNBC	E7449 (+ temozolomide / carboplatin and paclitaxel)	41
	2020	multi	I/II	NCT04644068 (recruiting) [16]	PETRA	advanced BC	AZD5305 (+ other chemotherapies)	840 (total)
	2022	multi	I/II	NCT05417594 (recruiting)		advanced BC	AZD9574 (+ other chemotherapies)	490 (total)
Target	Initiation Year	Country	Phase	Clinical trials	Name	Conditions	Drugs	BC sample (n)

Target	Initiation Year	Country	Phase	Clinical trials	Name	Conditions	Drugs	BC sample (n)
	2022	multi	I/II	NCT05252390 (recruiting)	NUV-868-01	advanced BC	NUV-868 (CDK4/6i) (+ olaparib + enzalutamide)	657 (total)
	2008	USA	II	NCT00813956 (completed) [17]	PrECOG 0105	TNBC, HER2- BC, *BRCA*m BC	iniparib + irinotecan + carboplatin	80
	2009	USA	II	NCT01009788 (active, not recruiting)		metastatic BC with(out) g*BRCA1/2*m	veliparib + temozolomide	41
	2010	USA	II	NCT01074970 (completed)		*BRCA*m TNBC	rucaparib + (cisplatin)	135
	2010	USA	II	NCT01173497 (completed) [18]	TBCRC 018	TNBC brain metastasis	iniparib + irinotecan	34
	2011	North America	II	NCT01306032 (completed)		TNBC	ABT-888 + cyclophosphamide	124
	2016	USA	II	NCT02595905 (active, not recruiting) [19]	S1416	metastatic or recurrent TNBC or g*BRCA1/2*m BC	veliparib + cisplatin	320
	2024	Germany	II	NCT06201234 (not yet recruiting)		advanced or metastatic hormone receptor+/HER2- BC	niraparib + elacestrant (selective estrogen receptor degrader)	176 (estimated)
	2014	multi	III	NCT02163694 (completed) [20]	BROCADE3	pretreated g*BRCA1/2*m advanced HER2- BC	veliparib + carboplatin or paclitaxel	513
	2014	multi	III	NCT02032277 (completed) [21]	BrighTNess	stage II-III TNBC	paclitaxel vs. paclitaxel + carboplatin vs. paclitaxel + carboplatin + veliparib	634

	2016	UK	III	NCT03150576 (recruiting) [22]	PARTNER	g*BRCA*wt TNBC	neoadjuvant olaparib + paclitaxel/carboplatin + anthracycline	559
	2017	Europe	III	NCT02810743 (active, not recruiting)	SUBITO	stage III, HER2- *BRCA1*-like BC	doxorubicin + cyclophosphamide-carboplatin/paclitaxel-olaparib; ddAC-mini CTC	174 (estimated)
NHEJ	2017	USA	II	NCT03193853 (completed) [23]	PIKTOR	TNBC	TAK-228 (TORC1/2 inhibitor) and TAK-117 (PI3Kα inhibitor)	10
ATR	2012	USA & UK	I	NCT02157792 (completed) [24]		advanced BC	VX-970/M6620 + carboplatin	1
CHEK	2011	USA & France	I	NCT01359696 (completed) [25]		refractory TNBC	GDC-0425 + gemcitabine	5

BC, breast cancer; TNBC, triple negative breast cancer; HR, hormone receptor; HER2, Human Epidermal Growth Factor Receptor 2; BRCAm, *BRCA1/2* mutationgBRCA1/2m, germline *BRCA1/2* mutation; g*BRCA*wt, germline *BRCA1/2* wildtype; PARP, poly ADP-ribose polymerase; po, orally; qd, once daily; bid, twice daily; q3w, every 3 weeks; iv, intravenously.

Supplementary Table 3. Clinical trials of therapies targeting DNA damage repair pathways combined with immunotherapy in breast cancer

	Initiation Year	Country	Phase	Clinical trials	Name	Conditions	Drugs	BC sample (n)
PARP	2015	USA	I	NCT02484404 (recruiting) [26]		TNBC	olaparib + durvalumab + cediranib (VEGFRi)	1
	2017	Europe	I	NCT03101280 (completed)		TNBC	rucaparib + atezolizumab	29
	2017	USA	I	NCT03061188 (unknown status)		recurrent /refractory unresectable stage IV BC	veliparib + nivolumab	15 (total)
	2017	USA	I	NCT03307785 (active, not recruiting) [27]	IOLite	advanced BC	niraparib, TSR-022 (anti-TIM3), bevacizumab, platinum based-chemotherapy + TSR-042 (anti-PD-1)	8
	2018	USA	I	NCT03544125 (completed)		metastatic TNBC	olaparib + durvalumab	3
	2018	Singapore	I	NCT03772561 (unknown status)		advanced or metastatic BC	olaparib + durvalumab + AZD5363	40 (estimated)
	2019	USA	I	NCT03842228 (active, not recruiting)		advanced BC with germline or somatic DDR mutations: *BARD1, BRCA1/2, BRIP1, FANCA, NBN, PALB2, RAD51, RAD51B/C/D*; *PTEN* mutations, or hotspot mutations in *PIK3CA*	olaparib, durvalumab, and copanlisib (PI3K inhibitor)	108 (estimated)
Target	Initiation Year	Country	Phase	Clinical trials	Name	Conditions	Drugs	BC sample (n)
	2021	USA	I	NCT04673448 (recruiting)		unresectable or metastatic *BRCA*m BC	niraparib + TSR-042 (dostarlimab)	18 (estimated)

2016	multi	I/II	NCT02734004 (active, not recruiting) [28]	MEDIOLA	metastatic *BRCA*m BC	olaparib + durvalumab	34
2016	USA	I/II	NCT02657889 (completed) [29]	TOPACIO/ KEYNOTE -162	advanced/metastatic TNBC with(out) *BRCA*m or PD-L1 expression	niraparib + pembrolizumab	55
2017	multi	I/II	NCT03330405 (terminated) [30]	JAVELIN PARP Medley	TNBC; hormone receptor+, HER2-, DDR+ BC (*BRCA/ATM*m)	avelumab + talazoparib	22; 23; 1
2018	South Korea	I/II	NCT03594396 (unknown status)		stage II-III TNBC or low ER+ BC	olaparib + durvalumab	54
2022	multi	I/II	NCT05252390 (recruiting)	NUV-868-0 1	advanced BC	NUV-868 (CDK4/6i) (+ olaparib + enzalutamide)	657 (total)
2023	China	I/II	NCT06078670 (not yet recruiting)		advanced TNBC	CVL218 + toripalimab + paclitaxel	96 (total)
2010	USA	II	NCT01042379 (recruiting) [31]	I-SPY2	non-pretreated stage II-III BC	olaparib (+ durvalumab)	950
2017	USA	II	NCT03025035 (recruiting)		advanced BC g*BRCA1/2*m or HRD	olaparib + pembrolizumab	18 (estimated)
2017	USA	II	NCT02849496 (active, not recruiting)		HRD advanced or metastatic HER2- BC	olaparib (+ atezolizumab)	20 (estimated)
2018	USA	II	NCT03801369 (recruiting)		metastatic TNBC with(out) g*BRCA1/2*m	olaparib + durvalumab, selumetinib/capivasertib, or ceralasertib monotherapy	132 (estimated)
2018	multi	II	NCT03167619 (completed)	DORA	platinum-pretreated advanced TNBC	olaparib (+ durvalumab)	45
2018	USA &	II	NCT03565991	JAVELIN	locally advanced or	talazoparib + avelumab	57

		Europe		(terminated) [32]	BRCA/ATM	metastatic BC with *BRCA/ATM*m		
	2019	multi	II	NCT0419113 (active, not recruiting) [33]	KEYLYNK-009	unresectable locally recurrent or metastatic TNBC	olaparib + pembrolizumab	462
	2019	France	II	NCT04053322 (recruiting)	DOLAF	ER+ HER2- locally advanced or metastatic BC with HRR genes alterations or MSI	olaparib + durvalumab + fulvestrant	173 (estimated）
	2019	Spain	II	NCT03931551 (terminated)	OPHELIA	HER2+ BC g*BRCA1/2*m or HRD	olaparib + trastuzumab	5
	2020	China	II	NCT04508803 (recruiting)		metastatic g*BRCA1/2*m BC	HX008 (PD-1i) + niraparib (+ trastuzumab/ pyrrolitinib)	37 (estimated)
	2020	USA	II	NCT04584255 (recruiting)		breast cancer with *BRCA1/2* and *PALB2* mutations	niraparib + dostarlimab	62 (estimated)
	2021	USA	II	NCT04837209 (recruiting)	NADiR	metastatic, PD-L1- or immunotherapy-refractory TNBC	niraparib + radiotherapy + dostarlimab	32 (estimated)
	2023	China	II	NCT05759546 (recruiting)		hormone receptor+/HER2- advanced BC	fluzoparib + dalpiciclib (CDK4/6i) + fulvestrant/AI (endocrinetherapy)	200 (estimated)
PARP + ATR	2019	UK	II	NCT03740893 (recruiting)	PHOENIX	neoadjuvant chemotherapy resistant residual TNBC	(neo)adjuvant olaparib + durvalumab + **AZD6738**	81 (estimated)
WT1	2019	USA	I/II	NCT03761914 (active, not recruiting)		TNBC	galinpepimut-S + pembrolizumab	15 (estimated)

BC, breast cancer; TNBC, triple negative breast cancer; HER2, Human Epidermal Growth Factor Receptor 2; ER, estrogen receptor; *BRCA*m, *BRCA1/2* mutation; g*BRCA1/2*m, germline *BRCA1/2* mutation; g*BRCA*wt, germline *BRCA1/2* wildtype; PARP, poly ADP-ribose polymerase; po, orally; qd, once daily; bid, twice daily; q3w, every 3 weeks; iv, intravenously.

Supplementary Table 4. Clinical trials of therapies targeting DNA damage repair pathways (PARPi and ATRi) combined with radiotherapies in breast cancer

Target	Initiation Year	Country	Phase	Clinical trials	Name	Conditions	Drugs	Breast cancer sample (n)
PARP	2017	France	I	NCT03109080 (completed) [34]	RadioPARP	inflammatory, loco-regionally advanced, or metastatic TNBC; operated TNBC with residual disease.	olaparib + radiotherapy	24
	2019	USA	I	NCT03945721 (recruiting)	UNITY	TNBC	niraparib + radiotherapy	20 (estimated)
	2019	North America	II	NCT03598257 (recruiting)		inflammatory BC	olaparib (+ radiotherapy)	300 (estimated)
	2021	USA	II	NCT04837209 (recruiting)	NADiR	metastatic, PD-L1- or immunotherapy-refractory TNBC	niraparib + radiotherapy + dostarlimab	32 (estimated)
ATR	2020	USA	I	NCT04052555 (active, not recruiting)		HER2- BC	berzosertib + radiotherapy	42 (estimated)

BC, breast cancer; TNBC, triple negative breast cancer; HER2, Human Epidermal Growth Factor Receptor 2; PD-L1, programmed cell death protein ligand 1. PARP, poly ADP-ribose polymerase; po, orally; qd, once daily; bid, twice daily; q3w, every 3 weeks; iv, intravenously.

Supplementary Table 5. Clinical trials of therapies targeting DNA damage repair pathways (PARPi) combined with targeted therapy in breast cancer

Initiation Year	Country	Phase	Clinical trials	Conditions	Drugs (PARPi)	BC sample (n)
2017	USA	I	NCT03075462 (completed)	TNBC	fluzoparib + apatinib (VEGFRi)	22
2017	USA	I	NCT02898207 (completed)	recurrent TNBC	olaparib + onalespib (Hsp90 inhibitor)	28
2017	USA	I	NCT03057145 (completed)	advanced BC	**olaparib + LY2606368 (CHEK1i)**	29 (total)
2019	USA	I	NCT03742245 (recruiting)	relapsed/refractory and/or metastatic BC	olaparib + vorinostat (HDACi)	28
2021	USA	I	NCT04703920 (recruiting)	metastatic BC	talazoparib + belinostat (HDACi)	25 (estimated)
2024	USA	I	NCT06130254 (recruiting)	*KRAS* G12C mutated BC	olaparib + adagrasib (*KRAS* G12Ci)	52 (estimated)
2012	USA	I/II	NCT01623349 (completed)	recurrent TNBC	olaparib + BKM120 or BYL719 (PI3Ki)	34; 17
2014	USA	I/II	NCT02208375 (active, not recruiting)	TNBC	olaparib + vistusertib (mTORC1/2i) / capivasertib (AKTi)	159
2019	USA	I/II	NCT03368729 (recruiting)	metastatic HER2+ BC	niraparib + trastuzumab	40 (estimated)
2010	North America	I/II	NCT01116648 (active, not recruiting)	recurrent TNBC	olaparib + cediranib maleate (VEGFRi)	180 (estimated)
2019	USA	I/II	NCT04039230 (active, not recruiting)	metastatic BC	talazoparib + antibody-drug conjugate sacituzumab goviteca	75 (estimated)

Initiation Year	Country	Phase	Clinical trials		Conditions	Drugs (PARPi)	BC sample (n)
2021	USA & UK	I/II	NCT04991480 (active, not recruiting)		advanced or metastatic BC	ART4215 (Polθ inhibitor) (+ olaparib/talazoparib)	390 (estimated)
2023	USA	I/II	NCT05898399 (recruiting)		advanced or metastatic HER2- *BRCA1/2*m BC	ART6043 (Polθ inhibitor) (+ olaparib or talazoparib)	250 (estimated)
2023	USA	I/II	NCT06065059 (recruiting)		*BRCA1/2*m or HRD BC	TNG348 (+ olaparib)	140 (estimated)
2016	North America	II	NCT02498613 (active, not recruiting)		unresectable or metastatic TNBC; stage III-IV BC	olaparib + cediranib (VEGFRi)	122 (total)
2018	multi	II	NCT03330847 (active, not recruiting)	VIOLETTE	TNBC with(out) HR-related gene mutations	**olaparib (+ ceralasertib (ATRi) or adavosertib (WEE1i))**	273
2020	USA	II	NCT04090567 (recruiting)		advanced or metastatic g*BRCA1/2*m BC	olaparib + cediranib (VEGFRi)	60 (estimated)
2020	multi	II	NCT04586335 (terminated)		advanced BC	olaparib + CYH33 (PI3Kαi)	24

BC, breast cancer; TNBC, triple negative breast cancer; HER2, Human Epidermal Growth Factor Receptor 2; BRCAm, *BRCA1/2* mutation; gBRCA1/2m, germline *BRCA1/2* mutation; gBRCAwt, germline *BRCA1/2* wildtype; PARP, poly ADP-ribose polymerase; HRR, homogenous recombination repair; po, orally; qd, once daily; bid, twice daily; q3w, every 3 weeks; iv, intravenously.

Supplementary Table 6. Clinical trials in breast cancer measuring or stratified by HRD assessment

clinical settings	initiation year	country	phase	clinical trials	name	BC conditions	interventions	biomarkers	measurement	sample	frequency/sample size	prognosis & therapeutic response
**HRD as inclusion criteria**	2015	USA	II	NCT02401347 (completed) [7]	TBB	pretreated advanced HER2- BC HRR-related gene mutations except *BRCA1/2*	talazoparib	genome scar	MyChoice CDx assay: HRD score TAI+LOH +LST ≥ 42	FFPE	100.0% (13)	ORR: 31% (4/13), CBR: 54% (7/13); All patients with germline mutated *PALB2* had treatment-associated tumor regression, ctDNA HRD scores predicted treatment outcomes and increased g*PALB2*m tumors
								‘*BRCAness*’ gene mutation signature	NGS	plasma ctDNA		
	2016	France	II	NCT02505048 (completed) [9]	RUBY	HRD BC except g*BRCA1/2*m	rucaparib	genome scar	genomic LOH score ≥ 18%	FFPE	high LOH: 87.5% (35/40); HRDetect: 85% (34/40); BC by LOH: 30.9% (220/711)	CBR: 13.5%; 2 LOH-high patients without somatic *BRCA1/2* mutation presented complete and durable response (12 and 28.5 months), HRDetect associated with rucaparib response; 220/711 metastatic breast cancer, high LOH without g*BRCA1/2*m benefit from PARP inhibitors; LOH alone not fully represent HRD
								‘*BRCAness*’ gene mutation signature	HRDetect			

clinical settings	initiation year	country	phase	clinical trials	name	BC conditions	interventions	biomarkers	measurement	sample	frequency/sample size	prognosis & therapeutic response
	2016	Germany	II	NCT02789332 (completed) [35]	GeparOLA	HER2- HRD BC	paclitaxel +olaparib vs. paclitaxel / carboplatin →epirubicin/ cyclophosphamide	genome scar	MyChoice CDx assay: HRD score TAI+LOH +LST ≥ 42	FFPE	62% HRD not *BRCA1/2*m	HRD TNBC higher pCR (olaparib arm: 56.0% vs. 52.0%; carboplatin arm: 59.3% vs. 20.0%) vs. HRP
								‘*BRCAness*’ gene mutation signature	s/g*BRCA1/2* mutations			
	2017	Japan	II	UMIN000023162 (completed) [36]	JBCRG 22	TNBC	HRD: neoadjuvant paclitaxel/eribulin + carboplatin followed by anthracycline; HRP: neoadjuvant eribulin + cyclophosphamide/capecitabine	genome scar	MyChoice CDx assay: HRD score TAI+LOH +LST ≥ 42	FFPE	HRD 64.7% (33/51); *BRCA1/2*m 21.8% (12/55)	*BRCA1/2*m or HRD not statistically associated with any immune cell density; high HRD or g *BRCA1/2*m suggest higher pCR
	2017	USA	I	NCT03061188 (unknown status)		recurrent or refractory unresectable stage IV BC	veliparib + nivolumab	‘*BRCAness*’ gene mutation signature	genomic profiling	tumor	15	NA
	2018	USA	II	NCT03344965 (active, not recruiting) [37]	TBCRC 048	metastatic BC with (s)*BRCA1/2*m or g/s HRR mutations except *BRCA1/2*	olaparib	‘*BRCAness*’ gene mutation signature	HRR 20-gene panel (*ATM, ATR, CDK12, FANCA, PALB2, RAD50*, etc.)	tumor or blood ctDNA	87% (47/54) HRRm except *BRCA1/2*	87% *PALB2, sBRCA1/2, ATM*, or CHEK2; ORR: g*PALB2*m 82%, s *BRCA1/2* 50%; mPFS: g*PALB2*m 13.3 months; s*BRCA1/2* 6.3 months; No responses in *ATM* or

clinical settings	initiation year	country	phase	clinical trials	name	BC conditions	interventions	biomarkers	measurement	sample	frequency/sample size	prognosis & therapeutic response
												CHEK2 mutations alone.
	2018	USA	II	NCT03367689 (terminated)	NOBROLA	*BRCA1/2*wt HRD BC	olaparib	'*BRCAness*' gene mutation signature	Foundation One assay	FFPE	7	NA
	2018	multi	II	NCT03330847 (active, not recruiting) [38]	VIOLETTE	TNBC with(out) HRR-related gene mutations	olaparib (+ ceralasertib (ATRi) or adavosertib (WEE1i))	'*BRCAness*' gene mutation signature	HRR gene panel	FFPE	54.4% (123/226)	HRD not predictive of DDR combined response
	2019	USA	I	NCT03842228 (active, not recruiting)		g/sDDRm BC (*BRCA1/2,FANCA,PALB2,RAD51,PTEN etc.*; *PTEN, PIK3CA* mutations,	copanlisib + olaparib (+ durvalumab)	'*BRCAness*' gene mutation signature	molecular profiling	pretreatment biopsy	108	NA
	2017	China	I/II	NCT03805399 (umbrella, recruiting) [39]	FUTURE	TNBC	PARPi	HRR/'*BRCAness*' gene mutation signature	FUSCC 500+ NGS gene panel testing	blood	g*BRCA1/2*m: 2.1% (3/141)	NA
	2017	Europe	III	NCT02810743 (active, not recruiting)	SUBITO	stage III, HER2- *BRCA1*-like BC	doxorubicin + cyclophosphamide-carboplatin/paclitaxel-olaparib; ddAC-mini-CTC	HRR/'*BRCAness*' gene mutation signature	*BRCA1*-like MLPA assay test	tumor	174	NA
	2019	France	II	NCT04053322 (recruiting)	DOLAF	ER+ HER2- locally advanced/metastatic BC with HRR genes alterations or	olaparib + durvalumab + fulvestrant	HRR/'*BRCAness*' gene mutation signature	HRR 19-gene panel (*ATM, ATR, CDK12, FANCA, PALB2, RAD50*, etc.)	tumor or blood	173	NA

clinical settings	initiation year	country	phase	clinical trials	name	BC conditions	interventions	biomarkers	measurement	sample	frequency/sample size	prognosis & therapeutic response
						MSI						
	2019	Spain	II	NCT03931551 (terminated)	OPHELIA	HER2+ BC g*BRCA1/2*m or HRD	olaparib + trastuzumab	HRR/'*BRCAness*' gene mutation signature	HRDetect	FFPE	5	NA
**post-hoc**	2007	USA	II	NCT00483223 (completed) [40]	TBCRC 009	TNBC	cisplatin or carboplatin	genome scar	HRD-LST/HRD-LOH assay	tumor	g*BRCA1/2*wt TNBC: 48.5% (32/66)	54.5% RR in g*BRCA1/2*m; BRCA-like genomic instability signature (HRD-LOH/HRD-LST score) predict response; HRD predictive of platinum response
	2007	USA	II	NCT00580333 (completed) [41]	Cisplatin1/2	stage II/III TNBC	neoadjuvant cisplatin (+bevacizumab)	genome scar<br>genome scar	HRD-TAI assay<br>HRD score	tumor<br>FFPE	high HRD-TAI: 57.3% (51/89)<br>high HRD score ≥10: 54% (26/48)	HRD represent better cisplatin response (RCB 0/I and pCR), especially in *BRCA1/2* nonmutated tumors
	2008	USA	II	NCT00813956 (completed) [17]	PrECOG 0105	TNBC	iniparib + irinotecan + carboplatin	genome scar	HRD-LOH assay	pretreatment core biopsy	high HRD-LOH≥10: TNBC 76.9% (50/65); g*BRCA1/2*wt 75.5%	higher HRD-LOH (without g*BRCA1/2*m) represent better PARP inhibitor response (pCR 42% vs 10%)

clinical settings	initiation year	country	phase	clinical trials	name	BC conditions	interventions	biomarkers	measurement	sample	frequency/sample size	prognosis & therapeutic response
								genome scar	HRD score	FFPE	(37/49) high HRD score≥42: TNBC 71% (48/68)	
	2008	UK	III	NCT00532727 (unknown status) [42]	TNT	TNBC	carboplatin + mechanistically distinct docetaxel	genome scar	MyChoice CDx assay: HRD score TAI+LOH +LST ≥ 42	pretreatment biopsy; blood	43.8% (86/196)	*BRCA1/2*m TNBC higher ORRs (68.0% vs 33.3%) and improved PFS (6.8 vs 4.4 months); but HRD TNBC not demonstrate statistically better ORR (38.2% vs 40.4%) and PFS
								BRCA1 methylation	disulfate sequencing and *BRCA1* mRNA expression			
								HRR/'*BRCAness*' gene mutation signature	g*BRCA1/2* mutations MLPA			
	2011	Germany	II/III	NCT01426880 (completed) [43]	GeparSixto	TNBC	carboplatin + anthracycline/taxane-based neoadjuvant chemotherapy	genome scar	MyChoice CDx assay: HRD score TAI+LOH +LST ≥ 42	FFPE	HRD 70.5% (136/193), 60.3% (82/136) without *BRCA1/2*m	HRD represent better pCR; HRD: adding carboplatin increase pCR 33.9%to 63.5%
								HRR/'*BRCAness*' gene mutation	s/g*BRCA1/2* mutations			

clinical settings	initiation year	country	phase	clinical trials	name	BC conditions	interventions	biomarkers	measurement	sample	frequency/sample size	prognosis & therapeutic response
								signature				
	2011	USA	II	NCT01372579 (unknown status) [44]		TNBC	neoadjuvant carboplatin + eribulin	genome scar	MyChoice CDx assay: HRD score TAI+LOH +LST ≥ 42	FFPE	46.2% (12/26)	HRD predictive of neoadjuvant carboplatin + eribulin response
								HRR/'*BRCAness*' gene mutation signature	s/g*BRCA1/2* mutations			
	2011	China	III	NCT01216111 (recruited) [45]	PATTERN	TNBC	paclitaxel + carboplatin vs. anthracyclines + docetaxel	HRR/'*BRCAness*' gene mutation signature	HRR 12-gene panel (*BRCA, ATM, ATR, FANCA, PALB2, RAD50*, etc.)	blood	25.4% (120/472) (66 *BRCA1/2*m, 54 non-*BRCA1/2*m)	HRRm better DFS with paclitaxel + carboplatin
	2012	Germany	III	NCT01583426 (completed) [46]	GeparSepto	BC	neoadjuvant nab-paclitaxel/paclitaxel	HRR/'*BRCAness*' gene mutation signature	mutational signatures S3 WES	pretreatment core FFPE	48.4% (120/248)	HRD predictive of better pCR
	2012	USA	I/II	NCT01623349 (completed) [47]		recurrent TNBC	olaparib + BKM120 or BYL719 (PI3Ki)	genome scar	HRD score: TAI+LOH +LST	FFPE	NA	HRD predictive of platinum and PARPi response; not HR deficiency did not correlate with immune activity
	2014	USA	II	NCT0198244	TBCRC 030	TNBC	neoadjuvant cisplatin +	genome scar	MyChoice	FFPE	71.1%	HRD not predictive of

clinical settings	initiation year	country	phase	clinical trials	name	BC conditions	interventions	biomarkers	measurement	sample	frequency/sample size	prognosis & therapeutic response
				8 (completed) [48]			paclitaxel		CDx assay: HRD score TAI+LOH +LST ≥ 33		(74/104)	pCR
	2014	UK	II	EudraCT 2014-003319-12 (completed) [26]	RIO	TNBC	rucaparib	HRR/'*BRCAness*' gene mutation signature	HRDetect	FFPE	69.2% (18/26)	HRD as accurate indicator of PARP inhibitor response with HRDetect; HRDetect more specific than HRD scores
								BRCA1 and *RAD51C* methylation	PCR	tumor		
								functional HRD assay	RAD51 IHC	FFPE		
	2016	Norway	II	NCT02624973 (active, not recruiting) [8]	PETREMAC	TNBC	olaparib	HRR/'*BRCAness*' gene mutation signature	360-gene panel; MLPA	pre-and post-biopsy	HR mutation: TNBC 34.4% (11/32), g*BRCA1/2*wt 22.2% (6/27); HR mutation and/or *BRCA1* methylation: TNBC 62.5% (11/32), *BRCAness* signature positive:	OR: olaparib (18/32, 56.3%); HRD (HR mutation and/or *BRCA1* methylation, even without g*BRCA1/2*m): 16/18, 88.9% vs. 4/14 non-responders; low RAD51 scores, high TIL, high PD-L1 correlated to olaparib response
								BRCA1 promoter methylation	methylation-specific quantitative PCR			
								functional HRD assay	RAD51 scores			

clinical settings	initiation year	country	phase	clinical trials	name	BC conditions	interventions	biomarkers	measurement	sample	frequency/sample size	prognosis & therapeutic response
											56.3%	
	2016	China	II	NCT03154749 (completed) [49]	NeoCART	TNBC	neoadjuvant carboplatin-based chemotherapy	genome scar	HRD score: allele-specific copy number and TAI, LOH, LST (high≥38)	pretreatment biopsy	HRD 69.8% (30/43); *BRCA1/2*m 18.6% (8/43)	no significant association between *BRCA1/2*m and pCR; higher HRD scores have docetaxel + carboplatin pCR
								‘*BRCAness*’ gene mutation signature	s/g*BRCA1/2* mutations			
	2016	USA	II	NCT02595905 (active, not recruiting) [19]	S1416	g*BRCA1/2*wt TNBC	veliparib + cisplatin	‘*BRCAness*’ gene mutation signature	40-gene panel BROCA-HR (except *BRCA1/2*)	FFPE	48.1% (101/257)	improved PFS in g*BRCA1/2*wt HRD TNBC with veliparib vs placebo adding to cisplatin (mPFS 5.9 vs 4.2 months)
								BRCA1 promoter methylation	PCR			
								genome scar	MyChoice CDx assay: HRD score TAI+LOH +LST ≥ 42			
	2017	Europe	I	NCT03101280 (completed)		TNBC	rucaparib + atezolizumab	genome scar	BRCA-like molecular signature	FFPE	29	NA
	2017	USA	II	NCT03193853 (completed)	PIKTOR	TNBC	TAK-228 (TORC1/2 inhibitor) and TAK-117	HRR/‘*BRCAness*’ gene	SBS signature 3	pre-and post-	10.0% (1/10)	patients could gain HRD feature after TORC1/2

clinical settings	initiation year	country	phase	clinical trials	name	BC conditions	interventions	biomarkers	measurement	sample	frequency/sample size	prognosis & therapeutic response
				[23]			(PI3Kα inhibitor)	mutation signature		biopsy		inhibitor and PI3Kα inhibitor treatment
	2017	USA	I	NCT03057145 (completed)		BC	olaparib + LY2606368 (CHEK1i)	functional DDR assay	γH2AX and comet assay	cells from biopsy tissues	29	NA
	2019	USA	I	NCT03955640 (recruiting) [50]		BC with chest wall recurrences	olaparib + hyperthermia treatment	functional HRD assay	RAD51 foci	biopsy	3	NA
								functional DDR assay	γH2AX and comet assay	cells from biopsy tissues		
**not interventional**	2006	USA	NA	NCT00896727 (completed) [51]	SWOG S9313	stage I-III BC	NA	genome scar	MyChoice CDx assay: HRD score TAI+LOH +LST $\geq$ 42	FFPE	67% (254/379) HRD (27% *BRCA1/2*m, 40% only HRD score >42)	HRD-positive status was associated with a better DFS and non-significant trend towards better OS
								BRCA1 promoter methylation	PCR			
	2010	Sweden	NA	NCT02306096 (recruiting) [52]	SCAN-B	TNBC	NA	*BRCA1* promoter hypermethylation	PCR	FFPE; frozen tumors	high HRDetect: 59% (150/254); hypermethylation: 21.3% (50/235)	high HRDetect has better outcomes on adjuvant chemotherapy regardless of epigenetic/genomic underlying mechanism
								HRR/'*BRCAness*' gene mutation	HRDetect			

clinical settings	initiation year	country	phase	clinical trials	name	BC conditions	interventions	biomarkers	measurement	sample	frequency/sample size	prognosis & therapeutic response
								signature				
	2014	USA	NA	NA [53]		BC	NA	HRR/'*BRCAness*' gene mutation signature	NGS	FFPE; frozen tumors	215	TNBC has highest HRD scores; HRD score correlated with *BRCA1/2* deficiency regardless of breast cancer subtypes
								genome scar	HRD-Mean score or optimal HRD model score of TAI, LOH, LST (SNP copy number)			
								BRCA1 promoter methylation	PCR; NGS			
	2017	Europe	NA	NA [54]		BC	NA	HRR/'*BRCAness*' gene mutation signature	HRDetect	FFPE	22.1% (124/560)	
	2019	Japan	NA	NA[55]		BC	neoadjuvant paclitaxel followed by fluorouracil, epirubicin, cyclophosphamide	*BRCA1* promoter hypermethylation	methylation-specific real-time PCR	FFPE	BC: 31.9% (45/141); TNBC: 60.5%; luminal A (5.3%), luminal B (HER2-)	HRD associated with high histological grade, Ki-67, PR-. HRD TNBC associated with low pCR
								genome scar	MyChoice CDx assay: HRD score TAI+LOH			

clinical settings	initiation year	country	phase	clinical trials	name	BC conditions	interventions	biomarkers	measurement	sample	frequency/sample size	prognosis & therapeutic response
									+LST ≥ 42		(28.8%), luminal B (HER2+) (31.6%)	
	2020	Japan	NA	NA[56]			neoadjuvant therapy	genome scar	MyChoice CDx assay: HRD score TAI+LOH +LST ≥ 42 (WES instead of SNP)	FFPE	BC: 25.0% (30/120); TNBC 66.7% (10/15); luminal 16.4% (10/61), luminal-HER 2 21.7% (5/23); HER2 23.8% (5/21)	HRD TNBC associated with low pCR; luminal HRD associated with favorable response to neoadjuvant paclitaxel, 5-fluorouracil/epirubicin/ cyclophosphamide.
	2017	Europe	I	NCT03101280 (completed)		TNBC	rucaparib + atezolizumab	genome scar	BRCA-like molecular signature	FFPE	29	NA

BC, breast cancer; TNBC, triple negative breast cancer; ER, estrogen receptor HER2, Human Epidermal Growth Factor Receptor 2; HRR, homogenous recombination repair; HRD, homogenous recombination deficiency; HRP, homogenous recombination proficiency; DDR, DNA damage repair; TAI: telomeric allelic imbalance; LOH: loss of heterozygosity; LST: large-scale state transition; germline *BRCA1/2* mutation; g*BRCA1/2*wt, germline *BRCA1/2* wildtype; HRRm, homogenous recombination repair-related gene mutations; PARP, poly ADP-ribose polymerase. PCR, polymerase chain reaction; IHC, immunohistochemistry; NGS, next-generation sequencing; ctDNA: circulating tumor DNA; FFPE, formalin fixed paraffin-embedded; ORR, overall response rate; (m)PFS: (median) progression free survival; CBR: clinical benefit rate; pCR, pathological complete response; NA: not applicable.

References

1. de Bono J, Ramanathan RK, Mina L, Chugh R, Glaspy J, Rafii S, et al. Phase I, Dose-Escalation, Two-Part Trial of the PARP Inhibitor Talazoparib in Patients with Advanced Germline BRCA1/2 Mutations and Selected Sporadic Cancers. Cancer Discov. 2017;7(6):620-9.
2. Mittra A, Coyne G, Zlott J, Kummar S, Meehan R, Rubinstein L, et al. Pharmacodynamic effects of the PARP inhibitor talazoparib (MDV3800, BMN 673) in patients with BRCA-mutated advanced solid tumors. Cancer Chemother Pharmacol. 2024;93(3):177-89.
3. Jiang Y-Z, Liu Y, Xiao Y, Hu X, Jiang L, Zuo W-J, et al. Molecular subtyping and genomic profiling expand precision medicine in refractory metastatic triple-negative breast cancer: the FUTURE trial. Cell Research. 2021;31(2):178-86.
4. Tutt A, Robson M, Garber JE, Domchek SM, Audeh MW, Weitzel JN, et al. Oral poly(ADP-ribose) polymerase inhibitor olaparib in patients with BRCA1 or BRCA2 mutations and advanced breast cancer: a proof-of-concept trial. Lancet. 2010;376(9737):235-44.
5. Gelmon KA, Tischkowitz M, Mackay H, Swenerton K, Robidoux A, Tonkin K, et al. Olaparib in patients with recurrent high-grade serous or poorly differentiated ovarian carcinoma or triple-negative breast cancer: a phase 2, multicentre, open-label, non-randomised study. Lancet Oncol. 2011;12(9):852-61.
6. Chopra N, Tovey H, Pearson A, Cutts R, Toms C, Proszek P, et al. Homologous recombination DNA repair deficiency and PARP inhibition activity in primary triple negative breast cancer. Nat Commun. 2020;11(1):2662.
7. Gruber JJ, Afghahi A, Timms K, DeWees A, Gross W, Aushev VN, et al. A phase II study of talazoparib monotherapy in patients with wild-type BRCA1 and BRCA2 with a mutation in other homologous recombination genes. Nat Cancer. 2022;3(10):1181-91.
8. Eikesdal HP, Yndestad S, Elzawahry A, Llop-Guevara A, Gilje B, Blix ES, et al. Olaparib monotherapy as primary treatment in unselected triple negative breast cancer. Ann Oncol. 2021;32(2):240-9.
9. Patsouris A, Diop K, Tredan O, Nenciu D, Goncalves A, Arnedos M, et al. Rucaparib in patients presenting a metastatic breast cancer with homologous recombination deficiency, without germline BRCA1/2 mutation. Eur J Cancer. 2021;159:283-95.
10. Tung NM, Robson ME, Ventz S, Santa-Maria CA, Nanda R, Marcom PK, et al. TBCRC 048: Phase II Study of Olaparib for Metastatic Breast Cancer and Mutations in Homologous Recombination-Related Genes. J Clin Oncol. 2020;38(36):4274-82.
11. Litton JK, Hurvitz SA, Mina LA, Rugo HS, Lee KH, Goncalves A, et al. Talazoparib versus chemotherapy in patients with germline BRCA1/2-mutated HER2-negative advanced breast cancer: final overall survival results from the EMBRACA trial. Ann Oncol. 2020;31(11):1526-35.
12. Robson ME, Tung N, Conte P, Im SA, Senkus E, Xu B, et al. OlympiAD final overall survival and tolerability results: Olaparib versus chemotherapy treatment of physician's choice in patients with a germline BRCA mutation and HER2-negative metastatic breast cancer. Ann Oncol. 2019;30(4):558-66.
13. Gelmon KA, Fasching PA, Couch FJ, Balmana J, Delaloge S, Labidi-Galy I, et al. Clinical effectiveness of olaparib monotherapy in germline BRCA-mutated, HER2-negative metastatic breast cancer in a real-world setting: phase IIIb LUCY interim analysis. Eur J Cancer. 2021;152:68-77.
14. Middleton MR, Dean E, Evans TRJ, Shapiro GI, Pollard J, Hendriks BS, et al. Phase 1 study of the ATR inhibitor berzosertib (formerly M6620, VX-970) combined with gemcitabine +/- cisplatin in patients with advanced solid tumours. Br J Cancer. 2021;125(4):510-9.
15. van der Noll R, Jager A, Ang JE, Marchetti S, Mergui-Roelvink MWJ, de Bono JS, et al. Phase I study of intermittent olaparib capsule or tablet dosing in combination with carboplatin

and paclitaxel (part 2). Invest New Drugs. 2020;38(4):1096-107.
16. Illuzzi G, Staniszewska AD, Gill SJ, Pike A, McWilliams L, Critchlow SE, et al. Preclinical Characterization of AZD5305, A Next-Generation, Highly Selective PARP1 Inhibitor and Trapper. Clin Cancer Res. 2022;28(21):4724-36.
17. Telli ML, Jensen KC, Vinayak S, Kurian AW, Lipson JA, Flaherty PJ, et al. Phase II Study of Gemcitabine, Carboplatin, and Iniparib As Neoadjuvant Therapy for Triple-Negative and BRCA1/2 Mutation-Associated Breast Cancer With Assessment of a Tumor-Based Measure of Genomic Instability: PrECOG 0105. J Clin Oncol. 2015;33(17):1895-901.
18. Anders C, Deal AM, Abramson V, Liu MC, Storniolo AM, Carpenter JT, et al. TBCRC 018: phase II study of iniparib in combination with irinotecan to treat progressive triple negative breast cancer brain metastases. Breast Cancer Res Treat. 2014;146(3):557-66.
19. Rodler E, Sharma P, Barlow WE, Gralow JR, Puhalla SL, Anders CK, et al. Cisplatin with veliparib or placebo in metastatic triple-negative breast cancer and BRCA mutation-associated breast cancer (S1416): a randomised, double-blind, placebo-controlled, phase 2 trial. Lancet Oncol. 2023;24(2):162-74.
20. Dieras V, Han HS, Kaufman B, Wildiers H, Friedlander M, Ayoub JP, et al. Veliparib with carboplatin and paclitaxel in BRCA-mutated advanced breast cancer (BROCADE3): a randomised, double-blind, placebo-controlled, phase 3 trial. Lancet Oncol. 2020;21(10):1269-82.
21. Loibl S, O'Shaughnessy J, Untch M, Sikov WM, Rugo HS, McKee MD, et al. Addition of the PARP inhibitor veliparib plus carboplatin or carboplatin alone to standard neoadjuvant chemotherapy in triple-negative breast cancer (BrighTNess): a randomised, phase 3 trial. Lancet Oncol. 2018;19(4):497-509.
22. Abraham JE, Pinilla K, Dayimu A, Grybowicz L, Demiris N, Harvey C, et al. The PARTNER trial of neoadjuvant olaparib in triple-negative breast cancer. Nature. 2024.
23. Lang JD, Nguyen TVV, Levin MK, Blas PE, Williams HL, Rodriguez ESR, et al. Pilot clinical trial and phenotypic analysis in chemotherapy-pretreated, metastatic triple-negative breast cancer patients treated with oral TAK-228 and TAK-117 (PIKTOR) to increase DNA damage repair deficiency followed by cisplatin and nab paclitaxel. Biomark Res. 2023;11(1):73.
24. Plummer ER, Dean EJ, Evans TRJ, Greystoke A, Middleton MR. Phase I trial of first-in-class ATR inhibitor VX-970 in combination with gemcitabine (Gem) in advanced solid tumors (NCT02157792). Journal of Clinical Oncology. 2016;34(15_suppl):2513-.
25. Infante JR, Hollebecque A, Postel-Vinay S, Bauer TM, Blackwood EM, Evangelista M, et al. Phase I Study of GDC-0425, a Checkpoint Kinase 1 Inhibitor, in Combination with Gemcitabine in Patients with Refractory Solid Tumors. Clin Cancer Res. 2017;23(10):2423-32.
26. Zimmer AS, Nichols E, Cimino-Mathews A, Peer C, Cao L, Lee MJ, et al. A phase I study of the PD-L1 inhibitor, durvalumab, in combination with a PARP inhibitor, olaparib, and a VEGFR1-3 inhibitor, cediranib, in recurrent women's cancers with biomarker analyses. J Immunother Cancer. 2019;7(1):197.
27. Yap TA, Bessudo A, Hamilton E, Sachdev J, Patel MR, Rodon J, et al. IOLite: phase 1b trial of doublet/triplet combinations of dostarlimab with niraparib, carboplatin-paclitaxel, with or without bevacizumab in patients with advanced cancer. J Immunother Cancer. 2022;10(3).
28. Domchek SM, Postel-Vinay S, Im S-A, Park YH, Delord J-P, Italiano A, et al. Olaparib and durvalumab in patients with germline BRCA-mutated metastatic breast cancer (MEDIOLA): an open-label, multicentre, phase 1/2, basket study. Lancet Oncology. 2020;21(9):1155-64.
29. Vinayak S, Tolaney SM, Schwartzberg L, Mita M, McCann G, Tan AR, et al. Open-label Clinical Trial of Niraparib Combined With Pembrolizumab for Treatment of Advanced or Metastatic Triple-Negative Breast Cancer. JAMA Oncol. 2019;5(8):1132-40.
30. Yap TA, Bardia A, Dvorkin M, Galsky MD, Beck JT, Wise DR, et al. Avelumab Plus Talazoparib in Patients With Advanced Solid Tumors: The JAVELIN PARP Medley Nonrandomized Controlled Trial. JAMA Oncol. 2023;9(1):40-50.

31. Nanda R, Liu MC, Yau C, Shatsky R, Pusztai L, Wallace A, et al. Effect of Pembrolizumab Plus Neoadjuvant Chemotherapy on Pathologic Complete Response in Women With Early-Stage Breast Cancer: An Analysis of the Ongoing Phase 2 Adaptively Randomized I-SPY2 Trial. JAMA Oncol. 2020;6(5):676-84.
32. Schram AM, Colombo N, Arrowsmith E, Narayan V, Yonemori K, Scambia G, et al. Avelumab Plus Talazoparib in Patients With BRCA1/2- or ATM-Altered Advanced Solid Tumors: Results From JAVELIN BRCA/ATM, an Open-Label, Multicenter, Phase 2b, Tumor-Agnostic Trial. JAMA Oncol. 2023;9(1):29-39.
33. Cussac AL, Rugo HS, Robson ME, Im SA, Dalenc F, Ruiz EY, et al. 198P Pembrolizumab plus olaparib vs pembrolizumab plus chemotherapy after induction with pembrolizumab plus chemotherapy for locally recurrent inoperable or metastatic TNBC: Patient-reported outcomes from KEYLYNK-009. ESMO Open. 2024;9.
34. Loap P, Loirat D, Berger F, Ricci F, Vincent-Salomon A, Ezzili C, et al. Combination of Olaparib and Radiation Therapy for Triple Negative Breast Cancer: Preliminary Results of the RADIOPARP Phase 1 Trial. Int J Radiat Oncol Biol Phys. 2021;109(2):436-40.
35. Fasching PA, Link T, Hauke J, Seither F, Jackisch C, Klare P, et al. Neoadjuvant paclitaxel/olaparib in comparison to paclitaxel/carboplatinum in patients with HER2-negative breast cancer and homologous recombination deficiency (GeparOLA study). Ann Oncol. 2021;32(1):49-57.
36. Ueno T, Kitano S, Masuda N, Ikarashi D, Yamashita M, Chiba T, et al. Immune microenvironment, homologous recombination deficiency, and therapeutic response to neoadjuvant chemotherapy in triple-negative breast cancer: Japan Breast Cancer Research Group (JBCRG)22 TR. BMC Med. 2022;20(1):136.
37. . !!! INVALID CITATION !!! [120].
38. Tutt A, Nowecki Z, Szoszkiewicz R, Im SA, Arkenau HT, Armstrong A, et al. 161O VIOLETTE: Randomised phase II study of olaparib (ola) + ceralasertib (cer) or adavosertib (ada) vs ola alone in patients (pts) with metastatic triple-negative breast cancer (mTNBC). Annals of Oncology. 2022;33:S194-S5.
39. Liu Y, Zhu XZ, Xiao Y, Wu SY, Zuo WJ, Yu Q, et al. Subtyping-based platform guides precision medicine for heavily pretreated metastatic triple-negative breast cancer: The FUTURE phase II umbrella clinical trial. Cell Res. 2023;33(5):389-402.
40. Isakoff SJ, Mayer EL, He L, Traina TA, Carey LA, Krag KJ, et al. TBCRC009: A Multicenter Phase II Clinical Trial of Platinum Monotherapy With Biomarker Assessment in Metastatic Triple-Negative Breast Cancer. J Clin Oncol. 2015;33(17):1902-9.
41. Telli ML, Timms KM, Reid J, Hennessy B, Mills GB, Jensen KC, et al. Homologous Recombination Deficiency (HRD) Score Predicts Response to Platinum-Containing Neoadjuvant Chemotherapy in Patients with Triple-Negative Breast Cancer. Clin Cancer Res. 2016;22(15):3764-73.
42. Tutt A, Tovey H, Cheang MCU, Kernaghan S, Kilburn L, Gazinska P, et al. Carboplatin in BRCA1/2-mutated and triple-negative breast cancer BRCAness subgroups: the TNT Trial. Nat Med. 2018;24(5):628-37.
43. Loibl S, Weber KE, Timms KM, Elkin EP, Hahnen E, Fasching PA, et al. Survival analysis of carboplatin added to an anthracycline/taxane-based neoadjuvant chemotherapy and HRD score as predictor of response-final results from GeparSixto. Ann Oncol. 2018;29(12):2341-7.
44. Kaklamani VG, Jeruss JS, Hughes E, Siziopikou K, Timms KM, Gutin A, et al. Phase II neoadjuvant clinical trial of carboplatin and eribulin in women with triple negative early-stage breast cancer (NCT01372579). Breast Cancer Res Treat. 2015;151(3):629-38.
45. Yu KD, Ye FG, He M, Fan L, Ma D, Mo M, et al. Effect of Adjuvant Paclitaxel and Carboplatin on Survival in Women With Triple-Negative Breast Cancer: A Phase 3 Randomized Clinical Trial. JAMA Oncol. 2020;6(9):1390-6.
46. Denkert C, Untch M, Benz S, Schneeweiss A, Weber KE, Schmatloch S, et al. Reconstructing tumor history in breast cancer: signatures of mutational processes and response to

neoadjuvant chemotherapy(small star, filled). Ann Oncol. 2021;32(4):500-11.
47. Przybytkowski E, Davis T, Hosny A, Eismann J, Matulonis UA, Wulf GM, et al. An immune-centric exploration of BRCA1 and BRCA2 germline mutation related breast and ovarian cancers. BMC Cancer. 2020;20(1):197.
48. Mayer EL, Abramson V, Jankowitz R, Falkson C, Marcom PK, Traina T, et al. TBCRC 030: a phase II study of preoperative cisplatin versus paclitaxel in triple-negative breast cancer: evaluating the homologous recombination deficiency (HRD) biomarker. Ann Oncol. 2020;31(11):1518-25.
49. Zhang L, Wu Z, Li J, Zhu D, Yang L, Shao Y, et al. Impact of Homologous Recombination Deficiency on Outcomes in Patients With Triple-Negative Breast Cancer Treated With Carboplatin-Based Neoadjuvant Chemotherapy: Secondary Analysis of the NeoCART Randomized Clinical Trial. JCO Precis Oncol. 2023;7:e2200337.
50. Mani C, Jonnalagadda S, Lingareddy J, Awasthi S, Gmeiner WH, Palle K. Prexasertib treatment induces homologous recombination deficiency and synergizes with olaparib in triple-negative breast cancer cells. Breast Cancer Res. 2019;21(1):104.
51. Sharma P, Barlow WE, Godwin AK, Pathak H, Isakova K, Williams D, et al. Impact of homologous recombination deficiency biomarkers on outcomes in patients with triple-negative breast cancer treated with adjuvant doxorubicin and cyclophosphamide (SWOG S9313). Ann Oncol. 2018;29(3):654-60.
52. Staaf J, Glodzik D, Bosch A, Vallon-Christersson J, Reutersward C, Hakkinen J, et al. Whole-genome sequencing of triple-negative breast cancers in a population-based clinical study. Nat Med. 2019;25(10):1526-33.
53. Timms KM, Abkevich V, Hughes E, Neff C, Reid J, Morris B, et al. Association of BRCA1/2 defects with genomic scores predictive of DNA damage repair deficiency among breast cancer subtypes. Breast Cancer Res. 2014;16(6):475.
54. Davies H, Glodzik D, Morganella S, Yates LR, Staaf J, Zou X, et al. HRDetect is a predictor of BRCA1 and BRCA2 deficiency based on mutational signatures. Nat Med. 2017;23(4):517-25.
55. Imanishi S, Naoi Y, Shimazu K, Shimoda M, Kagara N, Tanei T, et al. Clinicopathological analysis of homologous recombination-deficient breast cancers with special reference to response to neoadjuvant paclitaxel followed by FEC. Breast Cancer Res Treat. 2019;174(3):627-37.
56. Kim SJ, Sota Y, Naoi Y, Honma K, Kagara N, Miyake T, et al. Determining homologous recombination deficiency scores with whole exome sequencing and their association with responses to neoadjuvant chemotherapy in breast cancer. Transl Oncol. 2021;14(2):100986.

Supplementary Table 7. Studies on MSI-H/dMMR or related to mismatch repair in BC in recent 20 years.

Authors	Year	Country	median diagnosis age (yrs)	BC population	measurements	MSI-H frequency	dMMR frequency	percentage of each MMR protein and genes	BC subtype	histological stage	Ki-67 high
Muller et al.[1]	2002	USA	ND	LS-related BC	PCR	0% (0/27)		ND	ND		
Murata et al.[2]	2002	USA	ND	BC	IHC, PCR	20% (6/30)	16.7% (5/30)	60% reduced hMLH1 expression, 80% reduced hMSH2 expression	ND		
Adem et al.[3]	2003	USA	ND	hereditary / sporadic BC	IHC, PCR	0% (0/40) BC, 0% (0/30), hereditary BC		ND	ND		
de Leeuw et al.[4]	2003	The Netherlands	43.9	LS-related BC	PCR	63.6% (7/11)		ND	ND		
Kuligina et al.[5]	2007	Russia	ND	primary/secondary /contralateral BC	PCR	0% (0/52) primary, 10% (6/60) contralateral		ND	ND		
Blokhuis et al.[6]	2008	South Africa	46	BC with(out) *hMLH1* c.C1528T mutation	IHC, PCR	5.8% (4/69)		ND	ND		
Shanley et al.[7]	2009	UK	65.5	LS-related BC	IHC	80% (4/5)		75% (3) h*MLH1*, 25% (1) h*MSH2* mutation	ND	II or III	ND
Walsh et al.[8]	2010	Australia	57.5	BC from CRC families	IHC, PCR	17% (18/107)		83.3% loss of MSH2&MSH6 proteins, 27.7% MLH1&PMS2, 5.6% MSH6; 27.8% *MLH1*, 90.9% *MSH2*, 5.6% *MSH6* mutation	42% ER+, 33% PR+, 100% HER2-	60% III	ND
Jensen et al.[9]	2010	Denmark	50	LS-related BC	IHC, PCR	10% (2/20)	43.8% (7/16)	57.1% loss of MSH2$MSH6 proteins, 14.3% MLH1&PMS2, 14.3%	ND		

Authors	Year	Country	median diagnosis age (yrs)	BC population	measurements	MSI-H frequency	dMMR frequency	percentage of each MMR protein and genes	BC subtype	histological stage	Ki-67 high
								MSH6, 14.3% MSH2; 50% *MSH2*, 50% *MSH6* mutation			
Buerki et al.[10]	2012	Switzerland	56.5	LS-related BC with *MLH1/MSH2* germline mutations	IHC, PCR	85.7% (6/7)		50% *MLH1*, 50% *MSH2* mutation	ND		
Kamat et al.[11]	2012	Sweden	ND	BC receiving chemotherapy	IHC, PCR	18.6% (23/123)		39.1% Tp53-Alu, 31.0% Mfd41; Mfd28 25.3%; 4.6% Bat-26, 0% Bat-40; treatment-dependent loss of protein 29.3% hMLH1, 25.2% P53 and 18.7% hMSH2	ND	54% III, 30% II, 11% I, 5% VI	ND
Lotsari et al.[12]	2012	Finland	56	LS-related BC	IHC, PCR	34.8% (8/23)	65% (13/20)	47.8% *MLH1,* 21.7% *MSH2,* 30.4% *MSH6* mutation carriers; 40% MLH, 15% MSH2, 25% MSH6 protein loss	ER+ 90.9%, PR 68.2%, HER2+ 15%	ND	
Grandval et al.[13]	2012	France	53.5	LS-related BC	IHC, PCR	0% (0/14)		ND	ND		
Wen et al.[14]	2012	USA	57.5	TNBC or non-TNBC	IHC, PCR	0.5% (1/226)	1.8% (4/226)	75% lost MLH1&PMS2, 25% lost MSH2&MSH6 protein (no MSI-H/dMMR in non-TNBC)	TNBC	III	ND

Authors	Year	Country	median diagnosis age (yrs)	BC population	measurements	MSI-H frequency	dMMR frequency	percentage of each MMR protein and genes	BC subtype	histological stage	Ki-67 high
Crucianelli et al.[15]	2014	Italy	67	BC in MLH1 negative tumors	IHC, PCR	1 MSI-H + loss of MLH1 protein BC			ND		
Grindedal et al.[16]	2014	Norway	ND	BC with *PMS2* founder mutation c.989-1G > T	IHC, PCR	33.3% (1/3)	100% (3/3)	ND	ND		
Hirotsu et al.[17]	2015	Japan	ND	BC and hereditary breast and ovarian cancer	PCR, NGS, Sanger sequencing	1.9% (3/144)		ND	ND		
Hamm et al.[18]	2016	USA	ND	Inflammatory BC	NGS	25% *PMS2*, 16.7% *MSH2*, 16.7% *MSH6*, 8.3% *MLH1* mutation			85.7% HER2+	ND	
Hause et al.[19]	2016	/	ND	BC*	NGS (mSINGS)	ND		ND	ND		
Scott et al.[20]	2016	Australia	ND	Early-onset BC (<40 yrs at diagnosis)	PCR	0% (0/35)		ND	ND		
Bonneville et al.[21]	2017	/	ND	BC*	WES (MANTIS)	1.5% (16/1044)		ND	ND		
Cortes-Ciriano et al.[22]	2017	/	ND	BC*	WES	1.7% (16/922)		ND	ND		
Davies et al.[23]	2017	UK	ND	BC	IHC, WGS, WES	18.2% germline mutation (1 *MLH1*, 1 *PSM2*); 27.3% somatic mutation (2 *MLH1*, 1 *MSH2*)			45.5% ER+, 90.9% HER2+	ND	
Espenschied et al.[24]	2017	USA	52.9	*MMR*m BC	IHC, PCR, NGS	23.5% (124/528) had BC; 47.6% *PMS2*, 30.2% *MSH6*, 11.1% *MSH2/EPCAM*, 11.1% *MLH1* mutation			ND		
Halpern et al.[25]	2017	Israel	61.7	MSI-H BC	IHC, PCR	100% (11/11)		45.5% germline MMR mutation	55% ER+, 100% HER2-. 22% TNBC	44.5% III, 44.5% I, 11% II	ND
Le et al. [26]	2017	USA	ND	BC	IHC, PCR	<1%		ND	ND		
Mills et al.[27]	2018	USA	ND	TNBC & HER2+ BC	IHC	0.04%		33.3% loss of MSH2&MSH6 protein, 66.7% MLH1&MSH2;	1 TNBC, 2 ER+ metastases	1 I; 1 II; 1 III	ND

Authors	Year	Country	median diagnosis age (yrs)	BC population	measurements	MSI-H frequency	dMMR frequency	percentage of each MMR protein and genes	BC subtype	histological stage	Ki-67 high
Fusco et al.[28]	2018	Italy	65	BC	IHC, PCR	0.2% (1/444)	17% (75/444)	2.9% demonstrated any mutation in at least one of 4 MMR genes (963 in TCGA) 55%, 73%, 32%, 28% MLH1, MSH2, MSH6, PMS2 protein loss in 75 dMMR	28% Luminal A, 59% Luminal B, 1% HER2-, 12% TNBC	8% I; 43% II; 49% III	57%
Goodman et al.[29]	2018	/	ND	BC*	NGS	0% (0/5838)		ND	ND		
Kanaya et al.[30]	2018	Japan	63	LS-related BC	IHC, PCR	66.7% (2/3)		66.7% had loss of MLH1&PMS2 protein; 66.7% *MLH1*, 66.7% *MSH2* mutation	ER+ 83.3%, PR+ 83.3%, HER2+ 16.7%	33% I, 50% IIA; 7% IIB	
Nguyen et al.[31]	2018	Belgium	ND	BC diagnosed during pregnancy	WGS	ND	37.1% BCP associated with signature 20 attributable to dMMR (loss of MSH2)		ND		
Roberts et al.[32]	2018	USA	50.2	LS-related BC with (likely) pathogenic germline variants	NGS	ND		33.1% *MSH6*, 29.3% *PMS2*, 22.2% *MSH2*, 15.4% *MLH1* mutation	ND		
Saita et al.[33]	2018	Japan	66	LS-related BC	IHC	ND		lo+G28:H29ss of MLH1&PMS2 protein	ND		
Vanderwalde et al.[34]	2018	/	ND	BC*	IHC, PCR, NGS	0.6% (6/1024)		ND	ND		
Barroso-Sousa et al.[35]	2019	/	ND	hypermutated BC*	WES, NGS	signature 6, 15, and 20			ND		
Beca et al.[36]	2019	UK	42	acinic cell carcinomas of the	WES (MSIsensor)	33.3% (1/3)		MSIsensor score 10.93% (threshold	ER-HER2-	I	ND

Authors	Year	Country	median diagnosis age (yrs)	BC population	measurements	MSI-H frequency	dMMR frequency	percentage of each MMR protein and genes	BC subtype	histological stage	Ki-67 high
				breast				3.5%) with a pathogenic *MLH1* germline mutation (c.790+2dupT)			
Cheng et al.[37]	2019	USA	64.5%⩾50	BC	IHC	25 loss of single MMR biomarker (35.5% PMS2, 32.3% MLH1, 9.7% MSH6, 3.2% MSH2); 6 paired losses (12.9% MLH1&PMS2, 6.5% MSH2&MSH6)			Luminal A, 38.7% Luminal B, 6.5% HER2E, 12.9% TNBC	80.6% III, 19.4% I&II	55.6% Ki-67⩾ 14%
Gupta et al.[38]	2019	USA	ND	BC	NGS (MSIsensor)	0% (0/34)		ND	ND		
Hou et al.[39]	2019	USA	45.5	TNBC & HER2+ BC	IHC	ND	4.4% (13/298)	61.5% loss of MLH1&PMS2, 15.4% PMS2 loss, 23.1% MSH6; 1 TNBC complete loss of MLH1&PMS2 on whole tissue, the other 12 partial loss	53.8% TNBC, 46.2% HER2+ BC	84.6% III	ND
Jang et al.[40]	2019	/	ND	BC*; Luminal B+TNBC metastatic LN	single-cell RNA sequencing	ND	ND	ND	ND		
Latham et al.[41]	2019	USA	ND	BC	IHC, PCR	0% (0/2371)		42.9% *MSH6*, 42.9% *PMS2*, 14.2% *MLH1* mutation but all MSS	ND		
Lee et al.[42]	2019	South Korea	ND	HER2+ luminal B BC	PCR; IHC& MLH1 Sanger sequencing	0% (0/7)		germline mutations of *MLH1 V384D* in 13.8% (13/94), with a significantly high *TP53* mutations rate	ND		

Authors	Year	Country	median diagnosis age (yrs)	BC population	measurements	MSI-H frequency	dMMR frequency	percentage of each MMR protein and genes	BC subtype	histological stage	Ki-67 high
Lu et al.[43]	2019	USA	ND	BC	WES	0.4% (29/7601) *MSH6* mutation			20% ER+PR+HER2-, 3.1% ER-PR-HER+, 10.8% TNBC	ND	
Staaf et al.[44]	2019	USA	> 60	TNBC	IHC	ND		loss of MLH1&PMS2 expression	TNBC	II or III	100%
Trabucco et al.[45]	2019	/	ND	BC*	NGS	0.3% (24/7084)		ND	ND		
Vranic et al.[46]	2019	Italy	ND	cancers metastatic to the breast	NGS	0% (0/14)		ND	ND		
Zang et al.[47]	2019	China	ND	BC	WES	0.7% (1/151)	6.7% (10/151)	ND	HER2- BC exhibited 8.2% dMMR than 5.9% of HER2+	ND	
Falco et al.[48]	2020	Italy	ND	BC	NGS (F1CDX assay)	0% (0/7)		ND	ND		
Horimoto et al.[49]	2020	Japan	71	high density of tumor-infiltrating lymphocytes (TILs) 63 TNBC; 38 Medullary carcinomas (MedCa)	IHC, PCR	0% (0/101)	2.0% (2/101)	1 TIL-high TNBC case complete loss of MLH1&PMS2, 1 MedCa loss of PMS2 expression	1 TNBC 1 luminal HER2-	1 I; 1 IIA	ND
Huang et al.[50]	2020	China	ND	TNBC	IHC, PCR	0% (0/22)		1 *MSH3* mutation	ND		
Kurata et al.[51]	2020	Japan	77	TNBC	IHC	0.9% (2/228)		BAT-26, NR21, BAT-25 +	TNBC	III	100%
Long et al.[52]	2020	/	ND	BC*	NGS, WGS	5.5% (53/962)		ND	ND		
Lopez et al.[53]	2020	Italy	ND	BC	IHC	ND		55.6%, 64.2%, 49.4%, 18.5% loss of MLH1, MSH2, MSH6, PMS2 protein	27.2% luminal A, 60.5% luminal B, 14.8% HER2, 11.1% TNBC	More II/III than I	fewer than pMMR

Authors	Year	Country	median diagnosis age (yrs)	BC population	measurements	MSI-H frequency	dMMR frequency	percentage of each MMR protein and genes	BC subtype	histological stage	Ki-67 high
Nikitin et al.[54]	2020	Russia	48.4	hereditary /sporadic BC	NGS	4.5% (32/711) LS-related pathologic mutations		31.3% *MLH1*, 15.6% *MSH2*, 59.4% *MSH6*, 43.8% *PMS2,* 9.4% *EPCAM* mutations	TNBC 22%; HER2+ 34%; ER+PR+HER2-25%	ND	
Porkka et al.[55]	2020	Finland	53	LS-related BC	IHC, PCR, NGS	30% (6/20)	65% (11/20)	66.7% *MLH1*, 33.3% *MSH2* mutation; 63.6% MLH1, 18.2% MSH2, 18.2% MSH6 protein loss	83.3% ER+; 83.3% HER2-	ND	
Sheehan et al. [56]	2020	USA	46.7	LS-related BC	ND	27%, 3%, 4%, 9% PMS2, MLH1, MSH2, MSH6 LS patients had BC			ND		
Sivapiragasam et al.[57]	2020	USA	ND	metastatic BC	NGS	0.2% (7/3831); ER+HER2-:0.2% (2/1237); ER-HER2+: 0.1% (2/1953); TNBC: 0.4% (4/641)			71.4% ER-, 71.4% HER2-	ND	
Castaneda et al.[58]	2021	Peru	ND	male BC	IHC	15.4% (4/26) MMR protein paired loss			ND		
Chen et al.[59]	2021	China	ND	BC	NGS	0% (0/36)		ND	ND		
Ferrer-Avargues et al.[60]	2021	Spain	55	LS-related BC	NGS, Sanger sequencing	all the 2 had *MLH1* pathogenic mutation			ND		
Özcan et al.[61]	2021	Denmark	ND	TNBC	IHC	ND		8.3% MLH1, 11.1% MSH2, 8.3% MSH6, 5.6% PMS2	TNBC	ND	
Parvathareddy, et al.[62]	2021	Saudi Arabia	ND	BC, TNBC	IHC	ND		ND	BC	ND	
Ren et al.[63]	2021	China	68	TNBC	IHC, PCR, NGS	0% (0/440)	0.2% (1/440)	loss of MSH2 alone and MSI-L; a possible *EPCAM* deletion	TNBC	ND	35%
Schwartz et al. [64]	2021	USA	49.8	LS-related BC	IHC, PCR, NGS	36.4% (4/11)	41.7% (5/12)	60% MSH6, 40% MLH1, 40% PMS2,	80% ER+, 60% PR+, 80% HER2-	60% II, 40% III	ND

Authors	Year	Country	median diagnosis age (yrs)	BC population	measurements	MSI-H frequency	dMMR frequency	percentage of each MMR protein and genes	BC subtype	histological stage	Ki-67 high
Talbot et al.[65]	2021	Ireland	51	LS-related BC	ND	22.0% (9/41) carrying MSI-related mutations		20% MSH2 protein loss in dMMR BC; 45% *MSH6*, 27% *MLH1*, 18% *PMS2*, 9% *MSH2* mutation in BC pathological 22.2% *MLH1,* 44.4% *MSH2,* 22.2% *MSH6,* 0 *PSM2,* 11.1% *EPCAM* mutation	ND		
Venetis et al.[66]	2021	Italy	ND	BC	ND	2% (35/1807) MMRm ; 19% (363/1904) MMR mRNA mutations		0.6%&7% MLH1; 0.4%&4% MSH2; 0.8%&8% MSH6; 0.3%&6% PSM2	ND		
Wu et al.[67]	2021	China	ND	TNBC	IHC, PCR	0% (0/74)		ND	TNBC	ND	
Klouch et al.[68]	2022	USA	ND	BC	IHC, PCR (drop-off droplet digital PCR), NGS	1.8% (6/380)		66.7% loss of MLH1&PMS2 expression	ND		
Vidula et al.[69]	2022	USA	61	MBC	NGS (cfDNA)	42/6718 (0.63%)		ND	36% TNBC and 64% hormone receptor+/HER2-	ND	

ND, no data; LS, lynch syndrome; MSI-H, microsatellite instability high; dMMR, mismatch repair deficiency; BC*, breast cancer from public dataset (TCGA, etc.); MBC, metastatic breast cancer; TNBC, triple negative breast cancer; ER, estrogen receptor; PR, progestogen receptor; HER2, Human Epidermal Growth Factor Receptor 2; IHC, immunohistochemistry; PCR, polymerase chain reaction; NGS, next generation sequencing; TILs, tumor-infiltrating lymphocytes; cfDNA: cell-free DNA.

References

1. Muller A, Edmonston TB, Corao DA, Rose DG, Palazzo JP, Becker H, et al. Exclusion of breast cancer as an integral tumor of hereditary nonpolyposis colorectal cancer. Cancer Research.

2002;62(4):1014-9.
2. Murata H, Khattar NH, Gu L, Li GM. Roles of mismatch repair proteins hMSH2 and hMLH1 in the development of sporadic breast cancer. Cancer Lett. 2005;223(1):143-50.
3. Adem C, Soderberg CL, Cunningham JM, Reynolds C, Sebo TJ, Thibodeau SN, et al. Microsatellite instability in hereditary and sporadic breast cancers. Int J Cancer. 2003;107(4):580-2.
4. de Leeuw WJF, van Puijenbroek M, Tollenaar RAEM, Cornelisse CJ, Vasen HFA, Morreau H. Correspondence re: A. Muller et al., Exclusion of breast cancer as an integral tumor of hereditary nonpolyposis colorectal cancer. Cancer Res., 62 : 1014-1019, 2002. Cancer Research. 2003;63(5):1148-9.
5. Kuligina E, Grigoriev MY, Suspitsin EN, Buslov KG, Zaitseva OA, Yatsuk OS, et al. Microsatellite instability analysis of bilateral breast tumors suggests treatment-related origin of some contralateral malignancies. J Cancer Res Clin Oncol. 2007;133(1):57-64.
6. Blokhuis MM, Goldberg PA, Pietersen GE, Algar U, Vorster AA, Govender D, et al. The extracolonic cancer spectrum in females with the common 'South African' hMLH1 c.C1528T mutation. Fam Cancer. 2008;7(3):191-8.
7. Shanley S, Fung C, Milliken J, Leary J, Barnetson R, Schnitzler M, et al. Breast cancer immunohistochemistry can be useful in triage of some HNPCC families. Familial Cancer. 2009;8(3):251-5.
8. Walsh MD, Buchanan DD, Cummings MC, Pearson SA, Arnold ST, Clendenning M, et al. Lynch syndrome-associated breast cancers: clinicopathologic characteristics of a case series from the colon cancer family registry. Clin Cancer Res. 2010;16(7):2214-24.
9. Jensen UB, Sunde L, Timshel S, Halvarsson B, Nissen A, Bernstein I, et al. Mismatch repair defective breast cancer in the hereditary nonpolyposis colorectal cancer syndrome. Breast Cancer Res Treat. 2010;120(3):777-82.
10. Buerki N, Gautier L, Kovac M, Marra G, Buser M, Mueller H, et al. Evidence for breast cancer as an integral part of Lynch syndrome. Genes Chromosomes Cancer. 2012;51(1):83-91.
11. Kamat N, Khidhir MA, Jaloudi M, Hussain S, Alashari MM, Al Qawasmeh KH, et al. High incidence of microsatellite instability and loss of heterozygosity in three loci in breast cancer patients receiving chemotherapy: a prospective study. BMC Cancer. 2012;12:373.
12. Lotsari JE, Gylling A, Abdel-Rahman WM, Nieminen TT, Aittomaki K, Friman M, et al. Breast carcinoma and Lynch syndrome: molecular analysis of tumors arising in mutation carriers, non-carriers, and sporadic cases. Breast Cancer Res. 2012;14(3):R90.
13. Grandval P, Barouk-Simonet E, Bronner M, Buisine MP, Moretta J, Tinat J, et al. Is the controversy on breast cancer as part of the Lynch-related tumor spectrum still open? Fam Cancer. 2012;11(4):681-3.
14. Wen YH, Brogi E, Zeng Z, Akram M, Catalano J, Paty PB, et al. DNA mismatch repair deficiency in breast carcinoma: a pilot study of triple-negative and non-triple-negative tumors. Am J Surg Pathol. 2012;36(11):1700-8.
15. Crucianelli F, Tricarico R, Turchetti D, Gorelli G, Gensini F, Sestini R, et al. MLH1 constitutional and somatic methylation in patients with MLH1 negative tumors fulfilling the revised Bethesda criteria. Epigenetics. 2014;9(10):1431-8.
16. Grindedal EM, Aarset H, Bjornevoll I, Royset E, Maehle L, Stormorken A, et al. The Norwegian PMS2 founder mutation c.989-1G > T shows high penetrance of microsatellite instable cancers with normal immunohistochemistry. Hered Cancer Clin Pract. 2014;12(1):12.
17. Hirotsu Y, Nakagomi H, Sakamoto I, Amemiya K, Oyama T, Mochizuki H, et al. Multigene panel analysis identified germline mutations of DNA repair genes in breast and ovarian cancer. Mol Genet Genomic Med. 2015;3(5):459-66.
18. Hamm CA, Moran D, Rao K, Trusk PB, Pry K, Sausen M, et al. Genomic and Immunological Tumor Profiling Identifies Targetable Pathways and Extensive CD8+/PDL1+ Immune Infiltration in Inflammatory Breast Cancer Tumors. Mol Cancer Ther. 2016;15(7):1746-56.

19. Hause RJ, Pritchard CC, Shendure J, Salipante SJ. Classification and characterization of microsatellite instability across 18 cancer types. Nat Med. 2016;22(11):1342-50.
20. Scott CM, Joo JE, O'Callaghan N, Buchanan DD, Clendenning M, Giles GG, et al. Methylation of Breast Cancer Predisposition Genes in Early-Onset Breast Cancer: Australian Breast Cancer Family Registry. PLoS One. 2016;11(11):e0165436.
21. Bonneville R, Krook MA, Kautto EA, Miya J, Wing MR, Chen HZ, et al. Landscape of Microsatellite Instability Across 39 Cancer Types. JCO Precis Oncol. 2017;2017.
22. Cortes-Ciriano I, Lee S, Park WY, Kim TM, Park PJ. A molecular portrait of microsatellite instability across multiple cancers. Nat Commun. 2017;8:15180.
23. Davies H, Morganella S, Purdie CA, Jang SJ, Borgen E, Russnes H, et al. Whole-Genome Sequencing Reveals Breast Cancers with Mismatch Repair Deficiency. Cancer Res. 2017;77(18):4755-62.
24. Espenschied CR, LaDuca H, Li S, McFarland R, Gau CL, Hampel H. Multigene Panel Testing Provides a New Perspective on Lynch Syndrome. J Clin Oncol. 2017;35(22):2568-75.
25. Halpern N, Goldberg Y, Kadouri L, Duvdevani M, Hamburger T, Peretz T, et al. Clinical course and outcome of patients with high-level microsatellite instability cancers in a real-life setting: a retrospective analysis. Onco Targets Ther. 2017;10:1889-96.
26. Le DT, Durham JN, Smith KN, Wang H, Bartlett BR, Aulakh LK, et al. Mismatch repair deficiency predicts response of solid tumors to PD-1 blockade. Science. 2017;357(6349):409-13.
27. Mills AM, Dill EA, Moskaluk CA, Dziegielewski J, Bullock TN, Dillon PM. The Relationship Between Mismatch Repair Deficiency and PD-L1 Expression in Breast Carcinoma. Am J Surg Pathol. 2018;42(2):183-91.
28. Fusco N, Lopez G, Corti C, Pesenti C, Colapietro P, Ercoli G, et al. Mismatch Repair Protein Loss as a Prognostic and Predictive Biomarker in Breast Cancers Regardless of Microsatellite Instability. JNCI Cancer Spectr. 2018;2(4):pky056.
29. Goodman AM, Piccioni D, Kato S, Boichard A, Wang HY, Frampton G, et al. Prevalence of PDL1 Amplification and Preliminary Response to Immune Checkpoint Blockade in Solid Tumors. JAMA Oncol. 2018;4(9):1237-44.
30. Kanaya N, Tanakaya K, Yamasaki R, Arata T, Shigeyasu K, Aoki H, et al. Clinicopathological features of breast cancer in Japanese female patients with Lynch syndrome. Breast Cancer. 2019;26(3):359-64.
31. Nguyen B, Venet D, Azim HA, Jr., Brown D, Desmedt C, Lambertini M, et al. Breast cancer diagnosed during pregnancy is associated with enrichment of non-silent mutations, mismatch repair deficiency signature and mucin mutations. NPJ Breast Cancer. 2018;4:23.
32. Roberts ME, Jackson SA, Susswein LR, Zeinomar N, Ma X, Marshall ML, et al. MSH6 and PMS2 germ-line pathogenic variants implicated in Lynch syndrome are associated with breast cancer. Genet Med. 2018;20(10):1167-74.
33. Saita C, Yamaguchi T, Horiguchi SI, Yamada R, Takao M, Iijima T, et al. Tumor development in Japanese patients with Lynch syndrome. PLoS One. 2018;13(4):e0195572.
34. Vanderwalde A, Spetzler D, Xiao N, Gatalica Z, Marshall J. Microsatellite instability status determined by next-generation sequencing and compared with PD-L1 and tumor mutational burden in 11,348 patients. Cancer Med. 2018;7(3):746-56.
35. Barroso-Sousa R, Trippa L, Lange P, Andrews C, McArthur HL, Haley BB, et al. Nimbus: A phase II study of nivolumab plus ipilimumab in metastatic hypermutated HER2-negative breast cancer. Journal of Clinical Oncology. 2019;37(15).
36. Beca F, Lee SSK, Pareja F, Da Cruz Paula A, Selenica P, Ferrando L, et al. Whole-exome sequencing and RNA sequencing analyses of acinic cell carcinomas of the breast. Histopathology. 2019;75(6):931-7.
37. Cheng AS, Leung SCY, Gao D, Burugu S, Anurag M, Ellis MJ, et al. Mismatch repair protein loss in breast cancer: clinicopathological associations in a large British Columbia cohort. Breast Cancer Res Treat. 2020;179(1):3-10.

38. Gupta S, Vanderbilt CM, Cotzia P, Arias-Stella JA, 3rd, Chang JC, Zehir A, et al. Next-Generation Sequencing-Based Assessment of JAK2, PD-L1, and PD-L2 Copy Number Alterations at 9p24.1 in Breast Cancer: Potential Implications for Clinical Management. J Mol Diagn. 2019;21(2):307-17.
39. Hou Y, Nitta H, Parwani AV, Li Z. PD-L1 and CD8 are associated with deficient mismatch repair status in triple-negative and HER2-positive breast cancers. Hum Pathol. 2019;86:108-14.
40. Jang BS, Han W, Kim IA. Tumor mutation burden, immune checkpoint crosstalk and radiosensitivity in single-cell RNA sequencing data of breast cancer. Radiother Oncol. 2020;142:202-9.
41. Latham A, Srinivasan P, Kemel Y, Shia J, Bandlamudi C, Mandelker D, et al. Microsatellite Instability Is Associated With the Presence of Lynch Syndrome Pan-Cancer. Journal of Clinical Oncology. 2019;37(4):286-+.
42. Lee SE, Lee HS, Kim KY, Park JH, Roh H, Park HY, et al. High prevalence of the MLH1 V384D germline mutation in patients with HER2-positive luminal B breast cancer. Sci Rep. 2019;9(1):10966.
43. Lu HM, Li S, Black MH, Lee S, Hoiness R, Wu S, et al. Association of Breast and Ovarian Cancers With Predisposition Genes Identified by Large-Scale Sequencing. JAMA Oncol. 2019;5(1):51-7.
44. Staaf J, Glodzik D, Bosch A, Vallon-Christersson J, Reutersward C, Hakkinen J, et al. Whole-genome sequencing of triple-negative breast cancers in a population-based clinical study. Nat Med. 2019;25(10):1526-33.
45. Trabucco SE, Gowen K, Maund SL, Sanford E, Fabrizio DA, Hall MJ, et al. A Novel Next-Generation Sequencing Approach to Detecting Microsatellite Instability and Pan-Tumor Characterization of 1000 Microsatellite Instability-High Cases in 67,000 Patient Samples. J Mol Diagn. 2019;21(6):1053-66.
46. Vranic S, Palazzo J, Swensen J, Xiu J, Florento E, Gatalica Z. Theranostic molecular profiling of pleomorphic ductal carcinoma of the breast. Breast J. 2019;25(1):175-6.
47. Zeng Z, Vo A, Li X, Shidfar A, Saldana P, Blanco L, et al. Somatic genetic aberrations in benign breast disease and the risk of subsequent breast cancer. NPJ Breast Cancer. 2020;6:24.
48. De Falco V, Poliero L, Vitello PP, Ciardiello D, Vitale P, Zanaletti N, et al. Feasibility of next-generation sequencing in clinical practice: results of a pilot study in the Department of Precision Medicine at the University of Campania 'Luigi Vanvitelli'. ESMO Open. 2020;5(2).
49. Horimoto Y, Thinzar Hlaing M, Saeki H, Kitano S, Nakai K, Sasaki R, et al. Microsatellite instability and mismatch repair protein expressions in lymphocyte-predominant breast cancer. Cancer Sci. 2020;111(7):2647-54.
50. Huang X, Shao D, Wu H, Zhu C, Guo D, Zhou Y, et al. Genomic Profiling Comparison of Germline BRCA and Non-BRCA Carriers Reveals CCNE1 Amplification as a Risk Factor for Non-BRCA Carriers in Patients With Triple-Negative Breast Cancer. Front Oncol. 2020;10:583314.
51. Kurata K, Kubo M, Kai M, Mori H, Kawaji H, Kaneshiro K, et al. Microsatellite instability in Japanese female patients with triple-negative breast cancer. Breast Cancer. 2020;27(3):490-8.
52. Long DR, Waalkes A, Panicker VP, Hause RJ, Salipante SJ. Identifying Optimal Loci for the Molecular Diagnosis of Microsatellite Instability. Clin Chem. 2020;66(10):1310-8.
53. Brovkina OI, Shigapova L, Chudakova DA, Gordiev MG, Enikeev RF, Druzhkov MO, et al. The Ethnic-Specific Spectrum of Germline Nucleotide Variants in DNA Damage Response and Repair Genes in Hereditary Breast and Ovarian Cancer Patients of Tatar Descent. Front Oncol. 2018;8:421.
54. Nikitin AG, Chudakova DA, Enikeev RF, Sakaeva D, Druzhkov M, Shigapova LH, et al. Lynch Syndrome Germline Mutations in Breast Cancer: Next Generation Sequencing Case-Control Study of 1,263 Participants. Front Oncol. 2020;10:666.
55. Porkka NK, Olkinuora A, Kuopio T, Ahtiainen M, Eldfors S, Almusa H, et al. Does breast carcinoma belong to the Lynch syndrome tumor spectrum? - Somatic mutational profiles vs. ovarian and colorectal carcinomas. Oncotarget. 2020;11(14):1244-56.

56. Sheehan M, Heald B, Yanda C, Kelly ED, Grobmyer S, Eng C, et al. Investigating the Link between Lynch Syndrome and Breast Cancer. Eur J Breast Health. 2020;16(2):106-9.
57. Sivapiragasam A, Ashok Kumar P, Sokol ES, Albacker LA, Killian JK, Ramkissoon SH, et al. Predictive Biomarkers for Immune Checkpoint Inhibitors in Metastatic Breast Cancer. Cancer Med. 2021;10(1):53-61.
58. Castaneda CA, Castillo M, Bernabe LA, Sanchez J, Torres E, Suarez N, et al. A biomarker study in Peruvian males with breast cancer. World J Clin Oncol. 2021;12(10):926-34.
59. Chen A, Zhang S, Xiong L, Xi S, Tao R, Chen C, et al. Investigation of an Alternative Marker for Hypermutability Evaluation in Different Tumors. Genes (Basel). 2021;12(2).
60. Ferrer-Avargues R, Castillejo MI, Damaso E, Diez-Obrero V, Garrigos N, Molina T, et al. Co-occurrence of germline pathogenic variants for different hereditary cancer syndromes in patients with Lynch syndrome. Cancer Commun (Lond). 2021;41(3):218-28.
61. Ozcan D, Lade-Keller J, Tramm T. Can evaluation of mismatch repair defect and TILs increase the number of triple-negative breast cancer patients eligible for immunotherapy? Pathol Res Pract. 2021;226:153606.
62. Parvathareddy SK, Siraj AK, Ahmed SO, Ghazwani LO, Aldughaither SM, Al-Dayel F, et al. PD-L1 Protein Expression in Middle Eastern Breast Cancer Predicts Favorable Outcome in Triple-Negative Breast Cancer. Cells. 2021;10(2).
63. Ren XY, Song Y, Wang J, Chen LY, Pang JY, Zhou LR, et al. Mismatch Repair Deficiency and Microsatellite Instability in Triple-Negative Breast Cancer: A Retrospective Study of 440 Patients. Front Oncol. 2021;11:570623.
64. Schwartz CJ, da Silva EM, Marra A, Gazzo AM, Selenica P, Rai VK, et al. Morphological and genomic characteristics of breast cancers occurring in individuals with Lynch Syndrome. Clin Cancer Res. 2021.
65. Talbot A, O'Donovan E, Berkley E, Nolan C, Clarke R, Gallagher D. The contribution of Lynch syndrome to early onset malignancy in Ireland. BMC Cancer. 2021;21(1):617.
66. Venetis K, Fusco N, Sajjadi E. Commentary: Mismatch Repair Deficiency and Microsatellite Instability in Triple-Negative Breast Cancer: A Retrospective Study of 440 Patients. Front Oncol. 2021;11:735476.
67. Wu S, Shi X, Wang J, Wang X, Liu Y, Luo Y, et al. Triple-Negative Breast Cancer: Intact Mismatch Repair and Partial Co-Expression of PD-L1 and LAG-3. Front Immunol. 2021;12:561793.
68. Klouch KZ, Stern MH, Trabelsi-Grati O, Kiavue N, Cabel L, Silveira AB, et al. Microsatellite instability detection in breast cancer using drop-off droplet digital PCR. Oncogene. 2022.
69. Vidula N, Lipman A, Kato S, Weipert C, Hesler K, Azzi G, et al. Detection of microsatellite instability high (MSI-H) status by targeted plasma-based genotyping in metastatic breast cancer. NPJ Breast Cancer. 2022;8(1):117.
